# Magnetic Cellulose Nanocrystal Composites: Synthesis, Properties, Applications, and Opportunities

**DOI:** 10.3390/nano16110645

**Published:** 2026-05-22

**Authors:** Mohammad Jahid Hasan, Kishore Chand, Esteban E. Ureña-Benavides, Erick S. Vasquez-Guardado

**Affiliations:** 1 Department of Biomedical Engineering and Chemical Engineering, The University of Texas at San Antonio, San Antonio, TX 78249, USAesteban.urena-benavides@utsa.edu (E.E.U.-B.); 2 Department of Chemical and Materials Engineering, University of Dayton, 300 College Park Ave., Dayton, OH 45469, USA; chandk1@udayton.edu; 3 Hanley Sustainability Institute, University of Dayton, 300 College Park, Dayton, OH 45469, USA

**Keywords:** magnetic cellulose nanocrystals, cellulose nanocomposites, magnetic nanoparticles

## Abstract

Cellulose nanocrystals (CNCs) are abundant, renewable, biodegradable, non-toxic, and cost-effective nanomaterials with exceptional properties, making them highly appealing for nanocomposite material fabrication. Recognized for their sustainability, CNCs are emerging as promising substrates for the fabrication of functional, stimuli-responsive nanomaterials. This review highlights nanocomposites comprising magnetic nanoparticles with various forms of cellulose-based materials, with a primary focus on magnetic cellulose nanocrystal (MCNC) composites, yielding materials capable of controlled, on-demand responses to external magnetic fields. The magnetic properties of these nanocomposites can be precisely tuned by adjusting the magnetic nanoparticle content on CNC surfaces. At the nanoscale, magnetic CNCs exhibit remarkable properties, including facile and rapid magnetic separation, which holds great potential for numerous applications. This review examines the latest synthesis and modification methods for CNCs functionalized with various magnetic nanoparticles, as well as their applications in the biological, packaging, environmental, and biomedical fields.

## 1. Introduction

Cellulose, the world’s most abundant biomacromolecule, is a linear polysaccharide composed of β-D-glucopyranose units linked by (1→4)-glycosidic bonds. Its hierarchical structure includes both crystalline and amorphous domains. The crystalline regions can be isolated to produce cellulose nanocrystals (CNCs), which are rigid, rod-like nanoparticles. CNCs can be derived from renewable sources, such as wood, cotton, algae, and bacteria, offering a sustainable platform for advanced materials [1,2,3,4]. Native CNCs are hydrophilic due to abundant surface hydroxyl groups, which enables diverse chemical modifications such as carboxylation, sulfonation, phosphorylation, and grafting with polymeric or inorganic moieties. These strategies enhance compatibility with various matrices, improve interfacial adhesion, and enable controlled self-assembly, dispersion, and site-specific reactivity. They also facilitate the integration of CNCs into nanocomposites, hydrogels, and functional coatings, while introducing functionalities for catalysis, adsorption, drug delivery, and molecular recognition [5,6,7,8].

Magnetic nanoparticles (MNPs), particularly iron oxide nanostructures such as magnetite (Fe_3_O_4_) and maghemite (γ-Fe_2_O_3_), have attracted considerable research attention due to their superparamagnetic behavior, high surface-to-volume ratio, and biocompatibility [9]. These nanomaterials are synthesized using various methods, including co-precipitation, hydrothermal synthesis, thermal decomposition, and green or bio-template approaches, which allow for tunable size, magnetic properties, and surface chemistry [10,11,12,13,14,15]. In addition to classical iron oxide MNPs, other ferrite systems such as MnFe_2_O_4_ and CoFe_2_O_4_, rare earth ferrites, and hybrid MNPs are broadening the functional scope of magnetic nanotechnologies. MNPs have facilitated significant advances in environmental remediation, catalysis, targeted drug delivery, hyperthermia, biosensing, and magnetic separation [16].

The integration of CNCs with inorganic magnetic nanoparticles produces magnetic cellulose nanocrystal (MCNC). These nanocomposites overcome the limitations of individual components by providing a biocompatible, renewable, and tunable framework alongside magnetic responsiveness, separability, and actuation. As a result, MCNCs are promising for applications in smart responsive systems, environmentally friendly magnetic separation, biomedical applications, and sustainable devices [17,18]. MCNCs are typically synthesized via methods such as co-precipitation, hydrothermal and sol–gel processes, microemulsion, and self-assembly techniques [19,20]. These approaches enable control over nanoparticle dispersion and interfacial interactions. MCNCs exhibit anisotropic mechanical strength, colloidal stability, magnetic responsiveness, and functional versatility [17,18,20].

This review presents a comprehensive and critical overview of magnetic cellulose nanocrystal (MCNC), covering synthesis methodologies, structural and physicochemical properties, emerging applications, and future perspectives. The discussion systematically addresses (i) the fundamental properties, sources, synthesis, and surface modification of CNCs, (ii) the synthesis and characteristics of magnetic nanoparticles, (iii) the synthesis and characteristics of magnetic cellulose-based nanocomposites, with an emphasis on MCNC nanocomposites, (iv) state-of-the-art applications in environmental, catalytic, agricultural, biological, biomedical, optical, and structural materials, and (v) key challenges and opportunities related to scale-up, green processing, and the development of next-generation MCNC nanocomposites.

## 2. Synthesis of Cellulose Nanocrystals (CNC)

Cellulose is the most abundant biopolymer on Earth, with an estimated annual production of approximately 7.5 × 10^10^ tons. It serves as the primary structural component of plant cell walls due to its fibrous morphology, high crystallinity, mechanical strength, and insolubility in water. Cellulose can be obtained from a wide range of sources, including wood, cotton, bamboo, agricultural residues, algae, fungi, tunicates, and bacteria, resulting in variability in molecular weight, degree of polymerization, and microfibril organization. The spatial arrangement of hydroxyl groups at the C2, C3, and C6 enables extensive intra- and intermolecular hydrogen bonding, giving rise to hierarchical structures spanning from individual polymer chains to microfibrils and macroscopic fibers. First identified by Anselme Payen in 1838, cellulose remains a fundamental biological material and is widely used in textiles, paper, and advanced materials [3,21].

**Figure 1:**
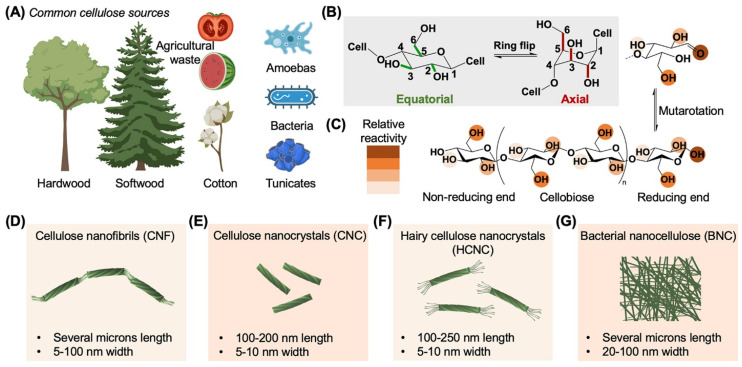
(**A**) Common sources of cellulose; (**B**) anhydroglucose ring conformation, highlighting the equatorial and axial orientations of hydroxyl groups in cellulose. (**C**) functional groups associated with each carbon atom of the anhydroglucose unit, including the aldehyde group at the reducing end and the primary and secondary hydroxyl groups. (**D**–**G**) Principal nanocellulose types: cellulose nanofibrils (CNF), cellulose nanocrystals (CNC), hairy cellulose nanocellulose (HCNC), and bacterial nanocellulose (BNC), shown with their characteristic dimensions. Reproduced from Mica L. Pitcher with permission from John Wiley and Sons, 2023, licensed under CC-BY-NC-ND [5].

Cellulose exists in multiple forms that differ in hierarchical organization, crystallinity, source, and nanoscale morphology. The primary classifications include native cellulose and nanocellulose. Native cellulose, known as cellulose I, is present in natural sources and exhibits a wide range of chain lengths, with wood cellulose containing approximately 10,000 glucose units per chain and cotton up to 15,000. The degree of polymerization and microfibril dimensions are strongly dependent on the biological source [22]. Nanocelluloses, produced through mechanical, enzymatic, or chemical treatments, are broadly classified into cellulose nanofibrils (CNF), cellulose nanocrystals (CNC), hairy cellulose nanocrystals (HCNC), and bacterial nanocellulose (BNC), as illustrated in Figure 1. CNFs are long, flexible fibrils with widths ranging from 5 to several hundred nanometers and contain both crystalline and amorphous domains. They are typically produced by mechanical fibrillation, often combined with chemical or enzymatic pretreatments to facilitate fibril separation [4]. CNCs, also known as nanowhiskers, are rigid, rod-shaped nanoparticles typically 100–200 nm in length and 5–10 nm in width, obtained by acid hydrolysis of cellulosic fibers, which selectively removes amorphous regions and yields materials with crystallinity indices typically exceeding 70% [18,22]. HCNCs represent a recently developed class characterized by crystalline cores with disordered cellulose chains (“hairs”) extending from their ends, commonly produced through selective chemical modification such as periodate oxidation [5]. BNC is synthesized extracellularly by bacteria such as *Komagataeibacter* spp. or *Acetobacter xylinus*, forming an ultra-pure, highly crystalline, three-dimensional network of entangled nanofibrils. This diversity of cellulose types nanostructures enables applications in nanocomposites, optoelectronics, filtration, packaging, and biomedical materials, while ongoing advances in chemical functionalization and nanoscale engineering continue to expand their performance and functionality [3,18,22].

Cellulose nanocrystals (CNCs) are renewable, high-performance nanomaterials derived from the hierarchical organization of cellulose found in diverse natural sources. In recent years, the range of feedstocks for CNC production has expanded beyond traditional wood and cotton to include agricultural residues, algae, fungi, tunicates, and bacterial cellulose. The biological origin and extraction method significantly influence the structural, chemical, and surface properties of the resulting CNCs, leading to variations in dimensions, crystallinity, surface chemistry, and functional performance [22,23].

### 2.1. Sources of Cellulose Nanocrystals

Wood remains the predominant industrial source of CNCs due to its abundance, high cellulose content, and well-established processing infrastructure. CNCs derived from hardwood and softwood typically exhibit high crystallinity and moderate aspect ratios, with lengths of 100–200 nm and diameters of 3–5 nm. Reported yields range from 12% to 30%, depending on wood species and extraction conditions [24]. Cotton, composed of nearly pure cellulose fibers, enables higher yields (10–40%) and produces CNCs with larger dimensions (100–400 nm in length), high crystallinity, and excellent dispersion stability, making them particularly attractive for biomedical and optoelectronic applications [22,25].

Agricultural residues—including sugarcane bagasse, rice straw, wheat straw, banana rachis, and papaya stems—have emerged as sustainable and cost-effective feedstocks that support circular bioeconomy initiatives. CNCs obtained from these sources exhibit aspect ratios and crystallinity comparable to those derived from wood and cotton, with properties tunable through pretreatment and hydrolysis conditions. For example, CNCs isolated from sugarcane bagasse typically range from 100–400 nm in length, with reported yields of 22–32%, highlighting their potential for resource-efficient nanomaterial production [26,27,28].

Non-traditional sources such as algae, fungi, and tunicates offer unique structural and functional advantages. Marine algae provide cellulose with distinct surface chemistry and tunable CNC morphology, while fungal systems offer integrated cellulase production that can facilitate nanocellulose extraction. Tunicates, marine invertebrates containing highly ordered cellulose, produce CNCs with exceptionally high aspect ratios, widths below 10 nm, lengths exceeding 1 μm, and high crystallinity. These features make tunicate-derived CNCs particularly attractive for high-performance composites and advanced biofunctional materials [29].

Bacterial cellulose, synthesized extracellularly by microorganisms such as *Komagataeibacter xylinus*, provides an ultra-pure cellulose source free of lignin and hemicellulose. CNCs derived from bacterial cellulose exhibit uniform dimensions, typically 100–200 nm in length and 3–5 nm in diameter, along with high crystallinity and excellent mechanical properties. In addition, bacterial production enables precise control over growth conditions, offering advantages in reproducibility, scalability, and material consistency for biomedical and advanced material applications [30,31,32].

The physicochemical properties of CNCs—including yield, crystallinity, aspect ratio, and surface chemistry—are strongly influenced by both the cellulose source and processing conditions. Parameters such as acid type, hydrolysis severity, and post-treatment purification govern the extent of amorphous region removal and surface functionalization, thereby affecting colloidal stability and reactivity. CNCs derived from wood, cotton, and bacterial cellulose can achieve crystallinity values approaching 90%, while variations in aspect ratio and surface accessibility reflect intrinsic differences in native microfibril structure [33,34].

The expanding diversity of cellulose feedstocks, coupled with advances in green processing and tailored pretreatment strategies, enables the rational selection of raw materials to achieve desired CNC properties. Continued research aimed at elucidating source-structure-processing-property relationships will further enhance the performance and applicability of CNCs in sustainable nanotechnology, advanced composites, and biofunctional materials [23,33,35,36,37,38].

### 2.2. Pretreatment of Cellulose for CNC Production

Pretreatment is a critical step in the efficient production of cellulose nanocrystals (CNCs), as it directly influences cellulose purity, extraction yield, morphology, and surface chemistry. Recent studies highlight that chemical and physical pretreatment strategies must be tailored to the specific cellulose source to optimize CNC properties and processing efficiency. Chemical pretreatments commonly include alkaline extraction and bleaching. Alkali treatment using sodium hydroxide, potassium hydroxide, or ammonia removes hemicellulose and lignin, disrupts intermolecular linkages, and promotes fiber swelling, thereby improving cellulose accessibility and apparent crystallinity. However, excessive alkali concentrations or prolonged exposure can lead to cellulose degradation. Multi-stage NaOH treatment has been shown to increase cellulose content from approximately 27% to 57%, while subsequent bleaching with oxidizing agents such as sodium chlorite or hydrogen peroxide removes residual lignin, achieving cellulose purities exceeding 87% and lignin removal efficiencies approaching 90%. In addition, alkaline pretreatment can significantly enhance CNC yield, for example, increasing recovery from approximately 14% to over 32% in agricultural residues [39,40,41].

Physical pretreatments such as milling, grinding, and high-pressure homogenization reduce fiber size, disrupt cell wall structure, and expose cellulose microfibrils, thereby facilitating subsequent hydrolysis. Ball milling, particularly when combined with alkaline pretreatment, enhances lignin removal and fiber swelling in agricultural residues such as cotton stalk and corn stover, improving hydrolysis efficiency. These processes can also influence CNC crystallinity and morphology; for instance, NaOH-treated cotton stalk retains a higher proportion of cellulose I crystalline structure, whereas corn stover may exhibit increased amorphization under similar conditions. Mechanical pretreatments increase accessible surface area and can produce CNCs with tunable dimensions and favorable thermal stability, depending on the source material and processing parameters [42,43].

The type, intensity, and sequence of pretreatment steps vary considerably with cellulose source. Woody biomass and agricultural residues typically require rigorous alkaline and bleaching treatments due to their high lignin and hemicellulose content, whereas cotton and bacterial cellulose, characterized by high intrinsic cellulose purity, require milder pretreatment conditions. Emerging approaches, including steam explosion, ionic liquid processing, and machine-learning-assisted process optimization, are being explored to improve CNC yield, purity, and energy efficiency while reducing environmental impact. Collectively, these advances enable more efficient and customizable CNC production from diverse feedstocks, supporting the scalable development of sustainable nanomaterials [44].

### 2.3. Extraction Methods of Cellulose Nanocrystals

The extraction of cellulose nanocrystals (CNCs) involves the selective removal of amorphous regions from purified cellulose fibers, with acid hydrolysis being the most widely employed method. Sulfuric acid hydrolysis, typically performed at concentrations of 50–65%, temperatures between 45 and 81 °C, and reaction times of 30–90 min, preferentially hydrolyzes amorphous domains while preserving crystalline regions. This process yields CNCs with high crystallinity, tunable aspect ratios, and negatively charged surface sulfate ester groups on the surface, which enhance electrostatic repulsion and improve colloidal stability in aqueous suspensions [40,45,46,47].

Hydrolysis conditions strongly influence CNC morphology and physicochemical properties. Increasing acid concentration, temperature, or reaction promotes more extensive removal of amorphous cellulose, generally resulting in shorter CNCs with higher surface charge density. However, excessively harsh conditions can reduce crystallinity, decrease aspect ratio, and lower overall yield due to partial degradation of crystalline domains. Optimized acid-to-cellulose ratios (typically 15:1 to 85:1, *w*/*w*) and reaction parameters, e.g., hydrolysis at 81 °C for 60 min, have been shown to produce stable CNC suspensions with high surface charge and uniform dimensions. Statistical optimization tools, including response surface methodology, are increasingly used to systematically evaluate process variables and maximize yield while minimizing degradation and aggregation [45,47,48].

Alternative mineral acids, such as hydrochloric, phosphoric, and nitric acids, provide additional control over CNC surface chemistry and properties. Hydrochloric acid hydrolysis produces highly crystalline CNCs with minimal surface functionalization, resulting in lower surface charge and reduced colloidal stability compared to sulfuric acid-derived CNCs. In contrast, phosphoric and certain organic acids can introduce functional groups, such as phosphate or carboxyl moieties, enabling enhanced surface reactivity. However, these methods often require higher temperatures or longer reaction times to achieve comparable hydrolysis efficiency [49].

Enzymatic hydrolysis is a milder, more environmentally friendly alternative, employing cellulase enzymes to selectively degrade amorphous cellulose under controlled conditions. While this approach preserves cellulose integrity and reduces chemical waste, it typically requires longer processing times and produces CNCs with larger dimensions and lower crystallinity compared to acid hydrolysis. TEMPO (2,2,6,6-Tetramethylpiperidine-1-oxyl)-mediated oxidation offers another versatile approach by selectively oxidizing primary hydroxyl groups at the C6 position to form carboxyl-functionalized CNCs with high surface charge and excellent aqueous dispersibility. Careful control of oxidation conditions is necessary to prevent excessive degradation and maintain crystallinity and surface integrity [17,50,51].

Precise control of hydrolysis parameters, including temperature, acid concentration, reaction time, and cellulose-to-acid ratio, is essential to balance CNC yield, crystallinity, aspect ratio, and surface functionality. As demand for sustainable nanomaterials continues to grow, advances in green extraction techniques, process optimization, and data-driven design are enabling more efficient, scalable, and tunable CNC production for advanced material applications [45,47].

### 2.4. Post-Extraction Processing and Treatment of CNC

Post-extraction processing is essential for producing stable, high-purity CNC suspensions and powders. Typically, repeated washing and centrifugation remove residual acids, salts, and degraded materials until the suspension reaches neutral pH, minimizing contaminants that may affect suspension stability or surface functionalization [37,40,52,53]. Dialysis is commonly used to eliminate small-molecule impurities and sulfate esters, producing stable suspensions with consistent zeta potentials. For large-scale production, membrane ultrafiltration and continuous-flow purification methods are being explored to reduce water and energy consumption while maintaining purification efficiency [54,55].

Following purification, sonication disperses CNCs into homogeneous suspensions, improving transparency, rheology, and film-forming ability. Multi-frequency ultrasonication can further enhance dispersion and provide partial control over CNC dimensions for optical and composite applications [56].

Drying converts CNC suspensions into storable powders. Freeze-drying preserves nanostructure and minimizes aggregation, yielding porous powders that redisperse easily, whereas spray-drying produces dense, free-flowing powders but may increase aggregation. Emerging methods, including ultrasonic or hybrid freeze-spray drying, aim to improve redispersibility and thermal stability while reducing energy consumption [56,57].

### 2.5. Source-Dependent CNC Characteristics

The source of cellulose and processing conditions strongly influence CNC dimensions, crystallinity, thermal stability, and dispersibility. CNCs derived from wood, cotton, and agricultural residues exhibit variations in yield and nanoscale properties that can be further tuned through extraction and post-treatment methods [24].

Wood-derived CNCs typically exhibit high crystallinity (70–90%) and rod-like structures, measuring 100–200 nm in length and 3–5 nm in diameter. Yields generally range from 12% to 30%, depending on species and hydrolysis conditions. These CNCs show good thermal stability (300–350 °C) and are widely used as reinforcing agents in polymer composites, packaging materials, and optoelectronic systems [58].

Cotton-derived CNCs possess high crystallinity (up to 85%) and larger aspect ratios, with lengths of 100–400 nm. Because cotton fibers consist of nearly pure cellulose I, mild pretreatments can yield 10–40% well-dispersed CNCs, making them attractive for biomedical and rheological applications [22,25,59,60,61,62].

Agricultural residues such as sugarcane bagasse, rice straw, and corn husk are increasingly used as sustainable CNC sources. These materials produce CNCs with dimensions comparable to those from wood and cotton (100–400 nm), although crystallinity and yields depend on purification and hydrolysis conditions. For example, sugarcane bagasse can yield 22–32% CNCs with crystallinity around 70–75%, suitable for biodegradable composites and filtration materials [6,24].

Post-processing steps such as desulfation, surface modification, and controlled drying can further tailor CNC thermal stability and dispersibility. CNCs produced via sulfuric acid hydrolysis possess surface sulfate groups that enhance aqueous dispersibility but reduce thermal stability; subsequent desulfation or functionalization can partially restore higher decomposition temperatures [54].

Overall, cellulose source and processing parameters determine CNC yield, morphology, crystallinity, and surface chemistry [63,64]. Common sources of cellulose nanocrystals (CNCs), along with their synthesis methods, typical sizes, and crystallinity, are summarized in Table 1. A detailed report on each source, synthesis method, and properties is provided in the Supporting Information (Table S1).

### 2.6. Sustainability and Scalability Considerations

Sustainability and scalability are central to CNC development, with increasing emphasis on resource efficiency and economic feasibility. Recent research has focused on renewable feedstocks such as agricultural residues, wood and cotton waste, and non-traditional sources including algae and fungi. These materials are abundant and support circular bioeconomy models by valorizing waste streams and reducing pressure on forest resources [38,46]. Agricultural byproducts such as papaya stems, coconut husks, and crop residues can produce CNCs with properties comparable to those derived from wood or cotton, enabling applications in water purification, packaging, biomedical materials, and optoelectronics [6,23].

Despite these advances, large-scale CNC production remains challenging. Conventional sulfuric acid hydrolysis is effective but requires large volumes of strong acids and generates acidic waste streams. Current efforts focus on improving process sustainability through acid recycling, reduced reagent and water consumption, ultrafiltration integration, and continuous reactor systems. Alternative approaches, including enzymatic, TEMPO-mediated, and organosolv treatments offer improved environmental compatibility but may involve longer reaction times, higher costs, or reduced yields [54,57].

Eco-design strategies and life cycle assessments (LCA) frameworks are increasingly used to evaluate CNC production pathways, showing opportunities to reduce energy use, emissions, and water consumption. Emerging tools such as machine learning and process intensification are also being applied to optimize feedstock selection, pretreatment conditions, and extraction parameters, improving efficiency and sustainability [103,104].

Overall, sustainable CNC production requires integrated approaches across the value chain, including responsible feedstock sourcing, closed-loop processing, and byproducts valorization. The recovery of lignin and hemicellulose for bioenergy or chemicals, along with modular processing units located near biomass sources, can further reduce transport emissions and enable decentralized manufacturing. These developments are positioning CNCs as scalable and environmentally responsible nanomaterials for advanced materials, environmental remediation, and energy applications [105,106,107].

### 2.7. Recent Advancements in CNC Synthesis and Surface Modification

Recent advances in cellulose nanocrystals (CNCs) research and development include machine-learning-guided synthesis, advanced surface functionalization, and expanding biomedical applications, significantly enhancing their versatility as functional nanomaterials. Machine learning models are increasingly used to predict CNC yield, aspect ratio, crystallinity, and dispersibility from experimental and sensor data. By integrating feedback from parameters such as titration, particle size, and zeta potential, these tools enable dynamic optimization of hydrolysis conditions (acid concentration, temperature, and reaction time), supporting scalable and resource-efficient CNC production with quality-by-design approaches [35,108].

Surface modification remains central to tailoring CNC properties. Chemical strategies such as TEMPO-mediated or periodate oxidation, esterification, etherification, and silylation introduce functional groups including carboxyl, aldehyde, hydroxyl, and silane moieties [65,109,110,111]. CNCs can also be functionalized through polymer grafting, thiol or amine coupling, and non-covalent adsorption, improving dispersibility, interfacial compatibility with polymer matrices, and surface charge. In addition, the incorporation of metallic nanoparticles, fluorescent labels, or magnetic species enables multifunctional CNCs for applications in composites, sensing, antimicrobial materials, and smart packaging [112,113].

Biomedical applications are rapidly expanding, with CNCs increasingly used in hydrogels for tissue engineering, wound healing, and implant coatings due to their biocompatibility, mechanical strength, and tunable architecture. Surface-functionalized CNCs have demonstrated capabilities in controlled drug delivery, antimicrobial activity, and biosensing [17,113]. Recent advances in high-throughput functionalization allow precise incorporation of bioactive or stimuli-responsive groups, enabling tailored interactions for regenerative medicine and environmental diagnostics [7,17,114].

Hybrid CNC-based nanocomposites are also emerging, combining CNCs with polymers, metals, or other nanomaterials through self-assembly, interfacial polymerization, or click chemistry. These systems enhance mechanical properties, barrier performance, and charge transport while enabling photonic, optoelectronic applications [7,20]. Integration with digital and additive manufacturing, and machine learning-guided processing further enable rapid prototyping of CNC-based hydrogels and composites for customized biomedical devices, filtration systems, and smart packaging [115,116,117,118].

**Figure 2:**
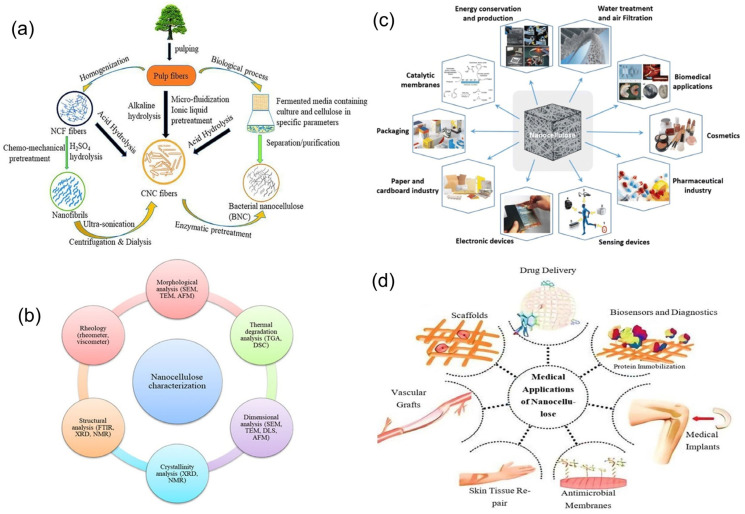
Schematic overview of cellulose nanocrystals (CNCs): (**a**) major sources and principal isolation methods; (**b**) characterization techniques used; (**c**) broad spectrum of advanced applications; (**d**) biomedical applications. Figure reproduced from Adib Bin Rashid and H.P.S. Abdul Khalil, Polymers, with copyright permission from MDPI 2023, and from Prabhpreet Kaur with copyright permission from Frontier Nanotechnology 2021 [1,4,121].

Overall, advances in predictive synthesis, surface functionalization, and multifunctional composite design are expanding CNC applications across biomedical, environmental, and industrial fields, reinforcing their potential as sustainable and highly versatile nanomaterials (Figure 2) [36,119,120].

## 3. Synthesis of Magnetic Nanoparticles (MNPs): An Overview

Magnetic nanoparticles (MNPs) are key nanomaterials in modern nanotechnology because of their unique magnetic properties and responsiveness to external magnetic fields. Typically ranging from 1–100 nm in size, MNPs exhibit behaviors distinct from bulk materials due to their high surface-to-volume ratio and quantum-size effects. These features enable applications in biomedicine, environmental remediation, catalysis, and advanced electronics [16]. In particular, iron oxide nanoparticles, such as magnetite (Fe_3_O_4_) and maghemite (γ-Fe_2_O_3_), are widely used due to their chemical stability, superparamagnetic behavior, and biocompatibility.

**Figure 3:**
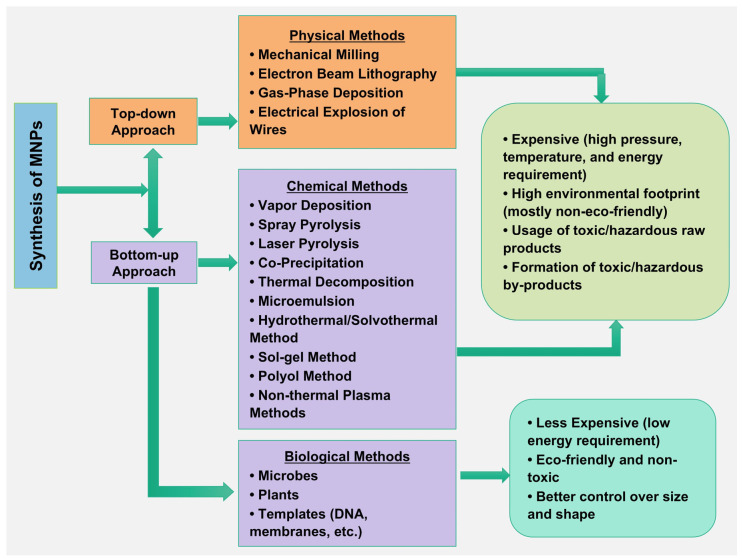
Synthesis method of magnetic nanomaterials. Figure reprinted with permission from Sourabh Shukla, Elsevier, 2021, licensed under CC-BY-4.0 [9].

MNPs are commonly synthesized using top-down and bottom-up approaches, which encompass physical, chemical, and biological synthesis routes (Section S1; Figure 3) [10,11,12,13,14,122,123,124,125,126,127,128,129,130,131,132,133,134,135,136,137,138,139,140,141,142,143,144,145,146,147,148,149,150,151,152,153,154]. Physical methods such as ball milling and laser ablation yield high-purity nanoparticles but offer poor size control and scalability. In contrast, chemical methods—including co-precipitation, hydrothermal synthesis, sol–gel processes, and thermal decomposition provide better control over size, crystallinity, and morphology. Among these, co-precipitation and hydrothermal synthesis are the most widely used because of their simplicity, scalability, and reproducibility.

For MCNC preparation, co-precipitation is particularly advantageous as it enables in situ deposition of MNPs onto CNCs, ensuring uniform dispersion and strong interfacial interactions, whereas thermal decomposition requires post-synthesis modification despite yielding highly uniform particles.

In recent years, biological or green synthesis methods have gained attention as environmentally friendly alternatives to conventional routes to produce MNPs. These approaches employ plant extracts, microorganisms, or biomolecules to reduce metal ions and form nanoparticles under mild conditions. While these approaches improve biocompatibility and reduce hazardous reagents, they often suffer from limited size control and reproducibility. Consequently, hybrid approaches combining chemical synthesis with green stabilization are increasingly employed to balance control, scalability, and sustainability. Overall, each synthesis method has strengths and limitations, and these are shown in Table 2.

The synthesis route strongly influences nanoparticle size, surface chemistry, and magnetic behavior, which are critical parameters for integration into functional nanocomposites. In particular, controlled synthesis of iron oxide MNPs with tunable surfaces enables their incorporation into cellulose nanocrystal (CNC) matrices, producing magnetic cellulose-based nanocomposites with enhanced functionality. These hybrid materials combine the mechanical strength, high surface area, and sustainability of CNCs with the magnetic responsiveness of MNPs, enabling applications in water purification, biomedical systems, catalysis, and responsive materials. The following sections describe the principal synthesis strategies for MNPs and their implications for the development of magnetic CNC-based nanocomposites.

## 4. Synthesis of Magnetic Cellulose Nanocrystal (MCNC) Nanocomposites

Magnetic cellulose nanocrystal (MCNC) nanocomposites combine the nanoscale structure, mechanical strength, and biocompatibility of CNCs with the responsive properties of MNPs. Their synthesis generally involves three key stages: (i) preparation of CNCs, (ii) synthesis of magnetic nanoparticles, and (iii) integration of both components into nanocomposite architectures. During in situ synthesis, Fe_3_O_4_ nanoparticles are nucleated and deposited directly onto the CNC surface in a single reaction step, effectively combining nanoparticle formation and composite assembly, sometimes followed by surface functionalization to improve stability or functionality.

**Figure 4:**
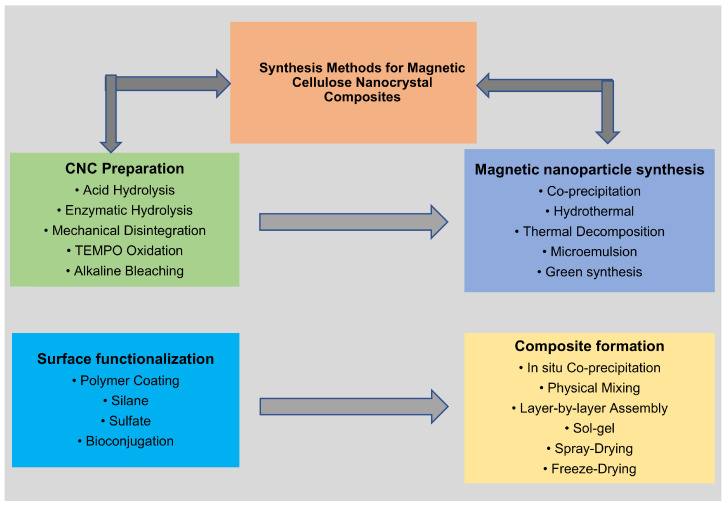
Schematic overview of common synthesis methods for magnetic cellulose nanocrystal (MCNC) nanocomposites, including representative procedures for CNC preparation, magnetic nanoparticle synthesis, composite formation strategies, and optional surface functionalization.

As illustrated in Figure 4, various chemical, physical, and biological approaches have been reported for each stage. CNCs are commonly obtained through acid hydrolysis, enzymatic treatment, or mechanical processing, while magnetic nanoparticles are typically synthesized by co-precipitation, hydrothermal, or green synthesis methods. Composite structures can then be formed through in situ growth, physical blending, or layer-by-layer assembly, depending on the targeted application [19].

The following subsections discuss these synthesis strategies in detail, highlighting the versatility of methods available for fabricating MCNC nanocomposites for biomedical, environmental, and engineering applications.

### 4.1. Co-Precipitation

Co-precipitation is one of the most widely used and scalable methods for synthesizing magnetic cellulose nanocrystal (MCNC) nanocomposites due to its simplicity and high efficiency in integrating iron oxide nanoparticles with cellulose matrices. In this approach, CNCs or cellulose derivatives are dispersed in an aqueous solution containing iron salts, typically Fe(II) and Fe(III) precursors, followed by the addition of a base such as NaOH or NH_4_OH [174,175,176]. During this process, Fe_3_O_4_ nanoparticles nucleate and grow directly on the CNC surfaces through interactions with abundant hydroxyl groups, forming stable magnetic composites. Reaction parameters—including temperature, pH, precursor ratios, and CNC type—strongly influence nanoparticle size, distribution, and the resulting magnetic properties.

**Figure 5:**
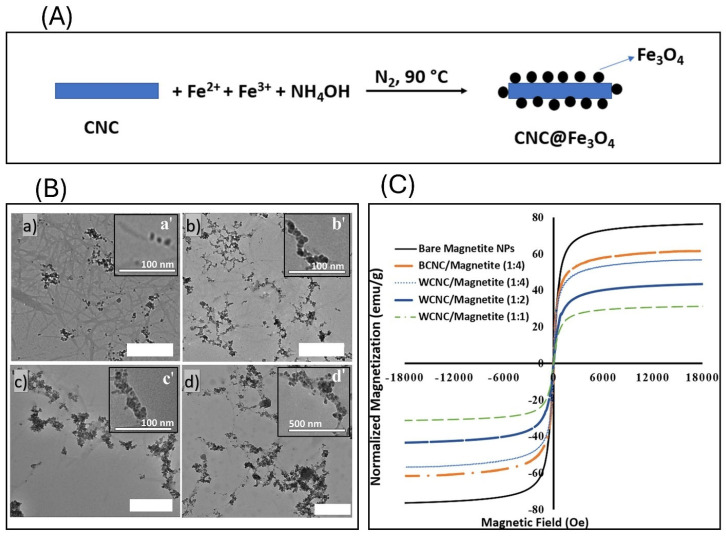
(**A**) Reaction scheme of magnetic cellulose nanocrystals; (**B**) TEM images of (**a**) wood-derived cellulose nanocrystals WCNC/Fe_3_O_4_ of 1:1, (**b**) WCNC/Fe_3_O_4_ of 1:2, (**c**) WCNC/Fe_3_O_4_ of 1:4, and (**d**) BCNC/Fe_3_O_4_ of 1:4; Thick scale bars on a, b, c, and d represent 500 nm; Insets a′, b′, c′, and d′ shows higher magnification images; (**C**) VSM magnetization curves of bare magnetite NPs and magnetic CNCs. Reproduced with permission from Mohammad J. Hasan et al., Cellulose (2021), Springer Nature [174].

For example, Hasan et al. (2021) synthesized magnetic CNCs via in situ co-precipitation using wood-pulp-derived CNCs and bacterial CNCs (BCNC). Iron(II) and iron(III) chlorides were added to CNC suspensions under nitrogen at 90 °C, followed by NH_4_OH addition to initiate magnetite formation [174]. The resulting nanocomposites exhibited improved nanoparticle coating and increased saturation magnetization with higher Fe_3_O_4_ loading (Figure 5) [174].

Recent studies have expanded co-precipitation strategies for magnetic cellulose nanocomposites. For example, ex situ co-precipitation within bacterial cellulose membranes has produced uniformly dispersed magnetite nanoparticles with crystallite sizes of ~6.9 nm and magnetization values near 50 emu g^−1^. Reverse co-precipitation using methylcellulose has also been reported to enhance light absorption and enable tunable magnetite loading. These approaches offer simplicity, low cost, and strong nanoparticle–cellulose interactions, making them attractive for applications such as electromagnetic shielding, drug delivery, and heavy-metal adsorption.

Despite its widespread use, co-precipitation presents several critical limitations. Rapid precipitation often leads to broad particle size distributions and nanoparticle agglomeration, reducing uniformity and reproducibility. In addition, highly crystalline CNC structures can hinder ion diffusion, limiting magnetite loading and interfacial integration. These factors collectively restrict scalability and consistent performance, particularly for applications requiring precise magnetic properties. Therefore, careful control of reaction conditions and cellulose substrate properties is essential to improve reproducibility, scalability, and overall performance [177,178,179].

### 4.2. Thermal Decomposition

Thermal decomposition is an effective strategy for synthesizing MCNC nanocomposites because it provides precise control over nanoparticle size, crystallinity, and morphology—properties critical for advanced functional performance. In this method, organometallic precursors such as iron(III) acetylacetonate or iron oleate are decomposed in high-boiling solvents (e.g., octyl ether or benzyl ether, 200–320 °C) in the presence of surfactants such as oleic acid or oleylamine. When CNCs are pre-dispersed in the reaction system, they act as heterogeneous nucleation sites that promote controlled nanoparticle growth and surface anchoring [180].

In this regard, Demisse et al. demonstrated that gas atmosphere plays a critical role in controlling nanoparticle structure during thermal decomposition. Maintaining an inert nitrogen flow below 50 mL min^−1^ favored the formation of magnetite (Fe_3_O_4_) cores with thin maghemite shells, producing highly crystalline nanoparticles with saturation magnetization values approaching bulk magnetite (~75 emu g^−1^). Controlled oxygen exposure further enabled well-defined core–shell structures with reduced defects and improved magnetic performance [180].

Extending these findings, recent studies have incorporated CNCs into thermally synthesized iron oxide systems to enhance interfacial interactions and composite stability. CNC-reinforced polymer composites, such as those based on polyhydroxyurethanes, have shown improved thermal stability, higher Young’s modulus, and increased char yield due to the reinforcing effect of cellulose [181,182]. These results collectively highlight the potential of thermally decomposed MCNC systems for advanced biomedical, electronic, and structural applications.

Despite its advantages, thermal decomposition presents several important limitations. The process typically requires high temperatures, expensive precursors, and strictly controlled reaction environments, which significantly limit scalability and production cost. In addition, nanoparticle aggregation and poor dispersion after synthesis often necessitate surface modification or post-treatment, particularly for effective integration with CNCs. These additional steps can complicate processing and reduce overall efficiency. Nevertheless, the method remains one of the most powerful routes for producing highly uniform magnetic nanoparticles for CNC-based nanocomposites [11,182,183,184].

### 4.3. Hydrothermal Method

Hydrothermal synthesis is widely recognized as a tunable and environmentally benign approach for fabricating MCNC nanocomposites. The process occurs in a water-based system under controlled temperature and pressure, enabling the in situ nucleation and growth of Fe_3_O_4_ nanoparticles on CNC templates without the need for external reducing or stabilizing agents. This approach promotes uniform nanoparticle deposition, strong interfacial bonding, and high crystallinity of the magnetic domains within the cellulose matrix [181].

For example, Soliman et al. reported a one-pot hydrothermal synthesis of Fe_3_O_4_–CNC nanocomposites using ferric and ferrous chlorides with urea as an in situ hydrolyzing agent. The resulting material exhibited superparamagnetic behavior and enhanced adsorption efficiency of doxycycline, attributed to the synergistic interaction between magnetite nanoparticle and surface functional groups on CNCs [181]. Similarly, Vu et al. (2023) developed an AgFe_3_O_4_@CNC nanocomposite catalyst capable of rapidly reducing 4-nitrophenol and dye pollutants, demonstrating efficient catalytic performance and magnetic recyclability [185].

Recent studies have also explored hydrothermally synthesized magnetic nanostructures with tailored morphologies and magnetic properties for applications in electromagnetic shielding, catalysis, and separation technologies [186,187]. The hydrothermal approach allows control over nanoparticle size and morphology—from spherical particles to hierarchical structures—by adjusting reaction temperature, precursor ratios, and solvent composition.

Despite its advantages, hydrothermal synthesis presents several practical limitations. This process often requires elevated pressures and relatively long reaction times, which reduce throughput and limit large-scale production. In addition, strict control of reaction conditions is often necessary to achieve consistent particle size and morphology, which can further complicate reproducibility. However, compared to other methods, hydrothermal synthesis offers lower toxicity, better control over crystallinity, and is highly compatible nanocellulose matrices, making it a promising and relatively sustainable method for producing multifunctional MCNC nanocomposites for environmental, catalytic, and electromagnetic applications [185,187].

### 4.4. Microemulsion Techniques

Microemulsion techniques provide a highly tunable approach for synthesizing MCNC nanocomposites by using nanoscale colloidal droplets as confined nanoreactors for iron oxide formation. In this method, water-in-oil (W/O) or oil-in-water (O/W) microemulsions are stabilized by surfactants such as Tween 80, Span 80, or sodium dodecyl sulfate. Iron salts (typically FeCl_2_ and FeCl_3_) are dissolved in the aqueous phase, while CNCs are incorporated into the microemulsion environment to promote uniform nucleation and growth of iron oxide nanoparticles within micellar cores. The addition of an alkaline agent, such as NH_4_OH, triggers in situ precipitation of iron oxide, producing nanoparticles that remain well dispersed and anchored to CNC surfaces through hydrogen bonding and electrostatic interactions [188].

In this context, Salehirozveh et al. (2024) demonstrated that controlling the size of water droplets in W/O micelles enables precise regulation of nanoparticle morphology, producing spherical maghemite (γ-Fe_2_O_3_) nanoparticles with sizes of 10–25 nm and strong superparamagnetic behavior [188]. Similarly, microemulsion-derived nanocellulose composites containing Fe_3_O_4_ nanoparticles (10–15 nm) have shown high structural uniformity and enhanced adsorption performance, achieving copper adsorption capacities of up to 149 mg g^−1^ and saturation magnetization of 35.9 emu g^−1^ [189]. In these systems, CNCs act not only as structural support but also as stabilizing agents that improve nanoparticle dispersion and provide functional groups for adsorption and catalytic interactions.

Despite its advantages in controlling nanoparticle size and dispersibility, the microemulsion approach presents several limitations related to surfactant optimization, reagent cost, and scalability. Excessive CNC loading can destabilize micelles and promote nanoparticle aggregation, while surfactant residues and organic solvents often require careful removal to ensure material purity, particularly for biomedical or catalytic applications. Nevertheless, microemulsion synthesis remains a powerful strategy for producing highly uniform magnetic nanocellulose composites compared to many conventional methods, provided that reaction parameters are carefully optimized [188,189].

### 4.5. Ultrasonic Irradiation Synthesis

Ultrasonic irradiation has emerged as an effective method for synthesizing MCNC nanocomposites by promoting rapid nanoparticle nucleation and dispersion through acoustic cavitation. The collapse of cavitation bubbles generates localized high temperatures and pressures (up to ~5000 K and ~1000 atm), facilitating the reduction in metal precursors and the deposition of iron oxide nanoparticles onto CNC surfaces. In a typical process, CNC suspensions containing Fe^2+^/Fe^3+^ salts are subjected to ultrasonic waves, enabling homogeneous nucleation and uniform nanoparticle growth along the CNC framework [190].

Ultrasonic-assisted synthesis often produces CNC@Fe_3_O_4_ nanocomposites with narrow particle size distributions (<50 nm), improved magnetic properties, and enhanced nanoparticle dispersion compared with conventional co-precipitation methods. For example, ultrasonication treatment of bacterial cellulose films functionalized with Fe_3_O_4_ nanoparticles resulted in improved nanoparticle distribution and enhanced mechanical flexibility due to cavitation-induced mixing and reduced magnetic clustering [191].

In addition to structural improvements, ultrasonically synthesized MCNC composites have shown promising performance in antimicrobial and environmental applications. Improved nanoparticle dispersion increases surface accessibility and adsorption capacity, while sonication-induced reactive oxygen species may contribute to antibacterial activity. However, excessive sonication can damage cellulose chains, reduce crystallinity, and weaken magnetic coupling. Therefore, careful control of ultrasonic power (typically 200–800 W), frequency (20–40 kHz), and exposure time is required to maintain structural integrity and optimize composite performance [190].

Despite these limitations, ultrasonic irradiation offers several advantages, including rapid synthesis, relatively low energy requirements, and improved nanoparticle dispersion. These characteristics make ultrasonic irradiation a promising approach for producing well-dispersed MCNC nanocomposites, particularly compared to conventional methods, for applications in antimicrobial materials, pollutant adsorption, and environmental remediation [178,192,193,194].

### 4.6. Microwave-Assisted Synthesis

Microwave-assisted synthesis has emerged as a rapid and energy-efficient approach for producing MCNC nanocomposites. This method relies on volumetric microwave heating, which enables uniform energy distribution and accelerated reaction kinetics. Typically, CNCs are dispersed in aqueous or mixed solvent systems containing iron precursors, such as FeCl_2_ or FeCl_3_, and microwave irradiation promotes homogeneous nucleation and in situ growth of magnetic nanoparticles directly on the CNC surface [195].

Microwave irradiation has also been widely explored for rapid CNC production and functionalization. For example, Amoroso et al. (2020) demonstrated that a microwave-assisted ammonium persulfate (APS) process reduced CNC preparation time from 16 h to 90 min while maintaining the cellulose Iβ crystalline structure and producing uniform nanocrystals. Similarly, Romero et al. reported microwave-assisted extraction of CNCs from almond shells, yielding materials with improved crystallinity and purity due to enhanced solvent penetration through fibers while expediting lignin and hemicellulose removal [195,196].

When applied to nanocomposite systems, microwave-assisted methods enable the rapid deposition of nanoparticles such as Fe_3_O_4_, Ag, or CuO, onto CNC matrices, forming multifunctional materials with improved catalytic and adsorption performance. These processes often produce monodisperse nanoparticles (typically <20–30 nm) with enhanced dispersion and strong interfacial integration within the cellulose framework. Such nanocomposites have been explored in water purification systems, catalytic materials, and hybrid hydrogel adsorbents with enhanced surface area and pollutant removal capacity [7].

Despite its advantages—including short reaction times, improved reproducibility, and reduced energy consumption—microwave-assisted synthesis presents several limitations, including limited penetration depth, reactor pressure constraints (below 400 psi), and difficulties in scaling to larger volumes. Metallic substrates can also interfere with electromagnetic field distribution, requiring careful reactor design and process optimization [195]. Nevertheless, microwave-assisted synthesis remains a promising strategy for producing high-performance, sustainable MCNC nanocomposites, particularly compared to conventional heating methods, through rapid and sustainable processing routes [197,198,199].

### 4.7. Other Synthesis Methods

In addition to conventional synthetic routes, several alternative strategies have been explored to produce MCNC nanocomposites with tailored functionalities. One important approach is in situ synthesis, where magnetic nanoparticles are generated directly within cellulose structures. For example, a one-pot solvothermal method using ferric chloride as both a hydrolytic agent for CNC formation and a precursor for Fe_3_O_4_ growth produced uniformly distributed nanoparticles (30 nm) embedded in CNC matrices with a magnetization of approximately 22 emu g^−1^ [200]. Similarly, wood-derived holocellulose–Fe_3_O_4_ hybrids prepared through in situ growth have demonstrated high heavy-metal adsorption capacity and improved nanoparticle dispersion compared with conventional coating methods [200,201].

In contrast to in situ strategies, chemical crosslinking strategies have also been used to enhance the structural stability and recyclability of MCNC nanocomposites. Bifunctional crosslinkers such as glutaraldehyde or N, N′-methylenebisacrylamide enable covalent bonding between CNC hydroxyl groups and nanoparticle coatings, forming robust composite frameworks. For instance, glutaraldehyde-crosslinked magnetic chitosan systems achieved up to 98% Cr(VI) removal with good reusability over multiple cycles, while CNC incorporation in crosslinked biopolymer films has been shown to significantly improve tensile strength and thermal stability [202,203].

On the other hand, simpler physical coating methods involve attaching pre-synthesized Fe_3_O_4_ nanoparticles onto CNC surfaces through electrostatic or van der Waals interactions, often assisted by ultrasonic dispersion. Although these approaches allow straightforward fabrication and tunable nanoparticle loading, weaker interfacial interactions can lead to particle detachment during repeated use [204]. In contrast, magnetic-field-assisted assembly enables controlled alignment of Fe_3_O_4_-decorated CNCs into anisotropic structures. For example, aligned CNC–Fe_3_O_4_ films have demonstrated high orientation order parameters (S = 0.98) and tunable optical anisotropy under low magnetic fields (<150 mT), producing transparent and magnetically responsive materials [205].

Overall, these emerging strategies broaden the design space for MCNC nanocomposites by enabling customizable architectures and tunable magnetic, optical, and adsorption properties. However, challenges persist: in situ and crosslinking approaches may require complex optimization, physical coating can suffer from limited stability, and magnetic-field-assisted methods face scalability limitations. Continued development and comparative evaluation of these techniques will be essential for advancing multifunctional MCNC materials for environmental, biomedical, and sensing applications [18,22,178,193,205].

### 4.8. Critical Comparison of MCNC Synthesis Methods

Although co-precipitation is widely used due to its simplicity and scalability, it often produces nanoparticles with broad size distributions and limited reproducibility in magnetic properties. These challenges can be mitigated to some extent by optimizing reaction kinetics, including careful control of pH, precursor concentration, and mixing conditions, as well as by modifying CNC surfaces to enhance nucleation and dispersion [177,178,179,206]. In comparison, thermal decomposition and microemulsion techniques offer greater control over particle size and uniformity, though they involve higher costs and more complex processing. Their compatibility with CNCs can be improved through post-synthesis surface modification or ligand exchange strategies to promote better dispersion and interfacial interactions [182,183,184,199,207]. Hydrothermal synthesis represents an intermediate approach, providing reasonable control over crystallinity and morphology, but it is often limited by longer reaction times and scalability constraints. These issues can be addressed through process optimization, such as tuning temperature–time profiles or employing microwave-assisted hydrothermal methods to enhance efficiency [181,185,187].

Overall, no single synthesis method is universally ideal; instead, the choice should be guided by the specific application requirements. Co-precipitation is particularly suitable for large-scale and cost-sensitive applications, whereas thermal decomposition and microemulsion methods are better suited for systems requiring precise structural control. Hydrothermal and hybrid strategies offer a balanced alternative, especially when both performance and scalability are important considerations. Table 3 summarizes a comparison of various MCNC synthesis methods, including their strengths, limitations, and potential solutions.

## 5. Properties of MCNC Nanocomposites and Characterization Techniques

MCNC nanocomposites have attracted growing interest due to the combination of CNC structural properties with the magnetic responsiveness of magnetic nanoparticles. These hybrid materials integrate the mechanical strength, anisotropic morphology, and biocompatibility of CNCs with the magnetic functionality of nanoparticles such as magnetite (Fe_3_O_4_) or maghemite (γ-Fe_2_O_3_). As a result MCNCs exhibit tunable structural, magnetic, and surface properties that support applications in catalysis, environmental remediation, biomedicine, and smart materials [18].

Understanding MCNC performance requires characterization of both the inorganic magnetic domains and the organic nanocellulose framework. Key properties include nanoparticle size and distribution, magnetic response to external fields, and surface chemistry that governs colloidal stability and functionality. These features are typically evaluated using complementary microscopy, spectroscopy, diffraction, and magnetometry techniques.

### 5.1. Morphological and Structural Analysis

Morphological and structural properties are critical parameters for understanding the behavior and performance of nanomaterials and nanocomposites. Key features such as particle size, shape, nanoparticle distribution, crystallinity, and overall composite architecture are commonly evaluated using several complementary characterization techniques.

Transmission electron microscopy (TEM) and scanning electron microscopy (SEM) are widely used to examine the size, morphology, and spatial distribution of nanoparticles within nanocomposite systems [174]. TEM imaging allows direct visualization of nanoscale structures and provides accurate measurements of particle dimensions and shape. In magnetic cellulose nanocrystal (MCNC) systems, magnetic nanoparticles (MNPs) embedded within the CNC matrix typically range from 1 to 100 nm in diameter. The CNCs themselves generally maintain rod-like geometries with diameters of approximately 3–20 nm and lengths of 100–500 nm, depending on the cellulose source and extraction method [22]. SEM is often used to complement TEM observations by providing information about surface morphology, particle aggregation, and the overall structural features of the composite materials.

The particle size and aspect ratio strongly influence the magnetic behavior of nanoparticles. For instance, magnetic nanoparticles smaller than ~25 nm typically exhibit superparamagnetism, where remanent magnetization disappears after removal of the external magnetic field, enabling strong magnetic responsiveness and good redispersibility for biomedical applications such as magnetic hyperthermia and targeted drug delivery [208].

Atomic force microscopy (AFM) is frequently employed to further analyze nanoscale surface topography and structural features. AFM provides three-dimensional information about the surface architecture of nanostructured materials and can be used to evaluate surface roughness, particle dispersion, and local morphological variations within composite systems [209].

The crystalline structure and phase composition of the nanoparticles are typically analyzed using X-ray diffraction (XRD) [175]. XRD patterns provide important structural information, including crystal phase identification, lattice structure, and the degree of crystallinity of the magnetic nanoparticles embedded within the composite matrix. This technique is particularly useful for confirming the formation of crystalline magnetite or related iron oxide phases and for evaluating structural stability within MCNC-based materials. In addition, small-angle X-ray scattering (SAXS) is commonly used to investigate nanoscale structural organization and particle size distribution in complex nanocomposite systems [210]. SAXS analysis provides insights into internal structural ordering, interparticle spacing, and the hierarchical architecture of nanoparticle–polymer networks.

### 5.2. Magnetic Behavior

Magnetic cellulose nanocrystal (MCNC) nanocomposites exhibit tunable magnetic behavior governed by nanoparticle size, crystallinity, and magnetic nanoparticle (MNP) loading. In most CNC-templated systems, iron oxide nanoparticles smaller than ~25 nm display superparamagnetism [208], which is typically verified through magnetic hysteresis measurements obtained using vibrating sample magnetometry (VSM) or the more sensitive superconducting quantum interference device (SQUID) magnetometry.

A key parameter describing magnetic performance is saturation magnetization (Ms), which reflects the maximum magnetization achieved under an applied field and is directly measured from VSM or SQUID magnetization curves [175,211]. Experimental results consistently show that Ms increases with increasing magnetic nanoparticle content, confirming that magnetization scales with the embedded magnetic phase fraction. For instance, Fe_3_O_4_-based MCNC systems typically exhibit Ms values in the range of 25–50 emu g^−1^,while higher values (up to ~70 emu g^−1^) have been observed in core–shell structures [212]. These trends, extracted from field-dependent magnetization measurements, demonstrate enhanced magnetic responsiveness and suitability for rapid magnetic separation.

**Figure 6:**
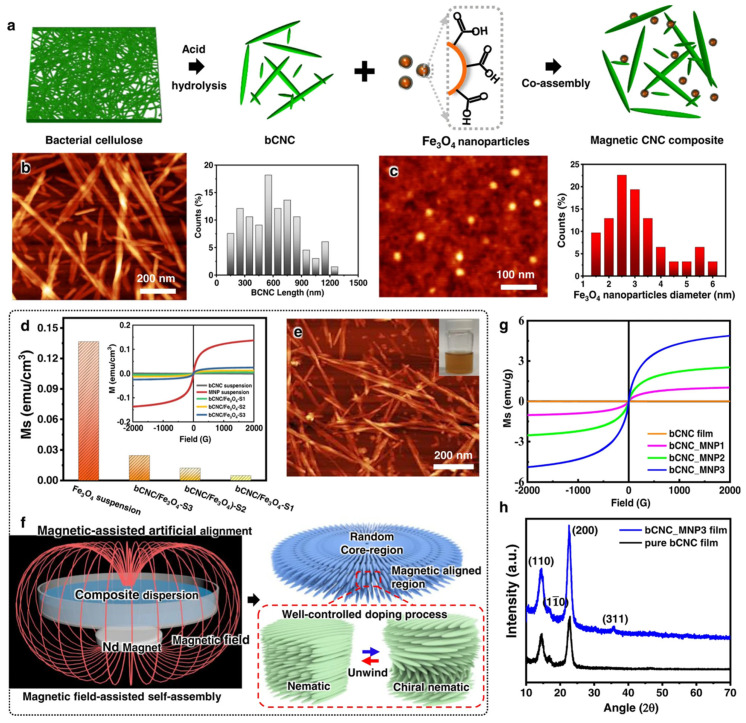
Magnetic-assisted co-assembly of cellulose nanocrystals and Fe_3_O_4_ nanoparticles and their properties, (**a**) Fabrication pathway for bCNC/Fe_3_O_4_ hybrids; (**b**) AFM image and length distribution of bCNCs; (**c**) AFM image and diameter distribution of Fe_3_O_4_ nanoparticles; (**d**) VSM data showing Ms of Fe_3_O_4_ and bCNC/Fe_3_O_4_ hybrids; (**e**) AFM of bCNC-Fe_3_O_4_ nanostructures; (**f**) Film-formation schematic under a 150 mT field; (**g**) Magnetization curves for bCNC film and bCNC_MNP1-3 films; (**h**) XRD peaks confirming Fe_3_O_4_ (311) and bCNC (110)/(200) planes. The figure is reproduced from Xiofang Zhang with copyright permission from Nature Communications CC BY 4.0 2022 [205].

Another important magnetic parameter, coercivity, is also derived from hysteresis loops measured by VSM or SQUID. MCNC nanocomposites generally exhibit low coercivity values (15–40 Oe), consistent with single-domain superparamagnetic systems [206,212]. This low coercivity indicates minimal energy loss during magnetization–demagnetization cycles and ensures reversible magnetic behavior, which is advantageous for applications such as targeted delivery and magnetic hyperthermia. At a more fundamental level, Mössbauer spectroscopy complements these measurements by probing the local iron environment, enabling identification of oxidation states, spin configurations, and magnetic phases that govern the observed coercivity and superparamagnetic response [213]. Figure 6 shows the magnetization characteristics and microstructural details of Fe_3_O_4_-decorated bacterial cellulose nanocrystals (bCNCs) prepared through magnetic-assisted co-assembly. The hysteresis loops confirm the superparamagnetic response with low coercivity and stable magnetic reversibility across bCNC_MNP1–3 films. The magnetization (Ms) increased proportionally with Fe_3_O_4_ content, from 0.96 emu g^−1^ for bCNC_MNP1 to 4.68 emu g^−1^ for bCNC_MNP3, validating the compositional control of magnetic responsiveness. The schematic (Figure 6) illustrates the role of a 150 mT magnetic field in directing uniaxial nematic ordering during film formation, yielding uniform magnetic alignment and orientation-dependent anisotropy [205].

In addition to bulk magnetic measurements, magnetic force microscopy (MFM) provides spatially resolved insight into magnetic domain distribution and alignment at the nanoscale [214]. This technique is particularly useful in structured systems such as films, where the application of an external magnetic field during fabrication induces magnetic alignment, leading to anisotropic organization of nanoparticles and orientation-dependent magnetic properties. MFM imaging directly visualizes these aligned domains, linking nanoscale structure to macroscopic magnetic behavior.

Beyond static magnetic properties, MCNC nanocomposites also exhibit efficient magnetothermal conversion under alternating magnetic fields, a behavior typically correlated with magnetization dynamics measured by VSM/SQUID. This behavior, governed by particle size and magnetic interactions, facilitates applications such as magnetic hyperthermia, where high specific absorption rate (SAR) values and consistent thermal stability can be attained. Recent investigations on cellulose-based magnetic nanocomposites further confirm efficient magnetothermal conversion under alternating magnetic fields. For example, a pectin–cellulose Fe_3_O_4_ hydrogel designed for in vitro hyperthermia exhibited SAR values up to 126 W g^−1^ and Ms ≈ 49 emu g^−1^, demonstrating strong magnetic functionality, thermal resilience, and size-dependent heat generation efficiency [215].

Overall, combining bulk magnetic measurements with nanoscale characterization techniques offers a thorough understanding of how superparamagnetism, saturation magnetization, coercivity, and magnetic alignment originate from the structural and compositional characteristics of MCNC nanocomposites.

### 5.3. Surface Chemistry and Colloidal Stability

Magnetic cellulose nanocrystal (MCNC) nanocomposites possess a high surface-to-volume ratio, providing abundant active sites for chemical functionalization and interfacial interactions. CNCs, rich in hydroxyl or carboxyl groups, facilitate anchoring of magnetic nanoparticles and subsequent modification with polymeric, metallic, or biomolecular ligands. This design flexibility enhances reactivity, improves colloidal stability, and enables specific surface functions, such as bioconjugation or catalytic activation. The combination of high surface area and surface tunability supports MCNCs’ roles in adsorption, drug immobilization, and photocatalysis [58].

Surface functionalization plays a central role in tailoring MCNC properties, and its success is typically verified through combined Fourier transform infrared (FTIR), X-ray photoelectron spectroscopy (XPS), and nuclear magnetic resonance (NMR) analyses. FTIR detects the formation of new chemical bonds following modifications such as silanization or polymer grafting, while Raman spectroscopy provides complementary structural information, particularly useful for assessing changes in crystallinity and molecular interactions. XPS offers quantitative analysis of elemental composition and chemical states within the top few nanometers of the surface, confirming the presence and distribution of functional groups [216]. A key advantage of XPS is its ability to provide quantitative surface composition with minimal sample preparation. It can also detect subtle changes in chemical bonding, making it ideal for studying surface modification and functionalization, with some instruments offering spatial mapping capabilities [217,218]. NMR spectroscopy, including diffusion-ordered spectroscopy (DOSY), provides additional insight into surface chemistry by revealing changes in chemical shifts, molecular interactions, and nanoparticle dispersion, thereby enabling detailed evaluation of functionalization efficiency and surface composition [219].

The physicochemical stability of MCNCs in suspension is strongly influenced by their surface chemistry and is commonly assessed using zeta potential measurements and dynamic light scattering (DLS) [175]. The presence of negatively charged functional groups on CNC surfaces generates electrostatic repulsion, which is reflected in high absolute zeta potential values, indicating good colloidal stability [65,220]. DLS measurements provide hydrodynamic size distributions and reveal aggregation behavior in different media, offering insight into dispersion quality and stability over time, although particle sizes may be overestimated due to hydration layers [221,222]. Surface functionalization further enhances stability by introducing steric hindrance and improving compatibility with aqueous and biological environments, thereby minimizing aggregation and preserving functional performance.

In addition, UV–Vis spectrophotometry is frequently used to monitor nanoparticle formation, dispersion, and concentration by analyzing changes in absorption spectra [223]. Variations in peak position and intensity provide information on particle size, shape, and interactions with the surrounding medium, enabling real-time assessment of colloidal stability and surface modifications. Together, these complementary techniques establish a direct relationship between surface area, functional groups, surface functionalization, and colloidal stability, providing a comprehensive understanding of how surface characteristics govern the reactivity, dispersion, and application performance of MCNC nanocomposites.

### 5.4. Complementary Characterization Techniques

The detailed study of magnetic cellulose nanocrystal (MCNC) nanocomposites often requires a multi-technique approach to fully capture their structural, compositional, and functional properties. Mass spectrometry (MSp) provides valuable insights into nanoparticle composition and surface chemistry [224]. For example, liquid chromatography-coupled MSp can reveal the nature and distribution of ligands, while matrix-assisted laser desorption/ionization (MALDI)-MSp identifies surface-bound capping agents and their size distributions [225,226]. Inductively coupled plasma MSp (ICP-MS) complements these analyses by quantifying elemental composition and detecting impurities [7], offering both qualitative and quantitative information when combined with other techniques. Although MSp can be limited by analyte fragmentation or challenges with thermally labile nanoparticles, it remains critical for confirming chemical structure and surface modification [225,226].

Surface area and porosity are typically assessed through Brunauer–Emmett–Teller (BET) analysis [227], which provides insights into the availability of reactive sites and supports correlation with adsorption, catalytic, or drug-loading capabilities. Thermal stability and compositional integrity are examined using thermal analysis techniques such as thermogravimetric analysis (TGA) and differential scanning calorimetry (DSC) [174], which track weight loss, decomposition, and phase transitions, providing complementary information on the stability of MCNCs under various conditions.

Advanced structural and magnetic characterization methods are increasingly employed to probe nanoscale features and interactions. In situ transmission electron microscopy (TEM) allows direct visualization of nanoparticle growth, distribution, and morphology under dynamic conditions [228], while nitrogen-vacancy (NV) diamond magnetometry enables high-sensitivity mapping of magnetic fields at the nanoscale, revealing local magnetic heterogeneity in composite architectures [229]. Energy-dispersive X-ray spectroscopy (EDS) paired with electron microscopy offers rapid elemental identification and spatial mapping, further confirming uniform Fe_3_O_4_ distribution in MCNCs [131].

Integrating techniques like mass spectrometry, BET, thermal analysis, advanced microscopy, and NV-diamond magnetometry provides a comprehensive understanding of MCNCs, linking composition, surface functionalization, and structure to functional performance. This multi-technique approach guides the rational design of next-generation magnetic cellulose nanocomposites for biomedical, environmental, and catalytic applications.

A comparative overview of representative studies reporting magnetic cellulose nanocomposites are shown in Supporting Information Table S2. It summarizes various synthesis methods—such as co-precipitation, hydrothermal, thermal decomposition, microemulsion, ultrasonic irradiation, microwave-assisted approaches, and others—together with the key physicochemical properties and intended applications of each system reported in the literature [59,200,204,230,231,232,233,234,235,236,237,238,239,240,241,242,243,244,245,246,247,248,249,250,251,252,253,254,255,256,257,258,259,260,261,262,263,264,265,266,267,268,269,270,271,272,273,274,275,276,277,278,279,280,281,282,283,284,285,286,287]. The comparative framework highlights the diversity of magnetic–cellulose hybrid design strategies and reveals correlations between synthesis approach, structural features, and functional performance, offering valuable insight for the rational engineering of next-generation magnetic cellulose nanocomposites.

## 6. Applications of Magnetic Cellulose Nanocrystal (MCNC) Composites

### 6.1. Environmental and Water Treatment Applications

Water pollution caused by heavy metals, dyes, oils, pharmaceuticals, and other organic and inorganic contaminants remains a major environmental and public health concern [288]. Conventional treatment technologies often face limitations in cost, selectivity, operational efficiency, and secondary waste generation. In this context, magnetic cellulose nanocrystals (MCNCs) have emerged as promising materials for environmental remediation and water treatment.

The potential of MCNC composites stems from several key features. CNCs provide a high specific surface area and abundant functional groups enabling strong interactions with diverse pollutants. Their surface chemistry can be tailored for selective adsorption, while embedded magnetic nanoparticles enable rapid magnetic separation and reuse.

Because of these advantages, MCNC-based composites have been widely explored for several environmental remediation applications, including heavy-metal ion adsorption, dye removal, oil-water separation via Pickering emulsions, and other pollutant removal processes. The following sections summarize key advances in each of these areas.

#### 6.1.1. Heavy Metal Ion Adsorption

Heavy metal contamination in aquatic environments poses a severe threat to human health and ecosystems, prompting extensive research into sustainable and efficient adsorbents for water purification. Among various biopolymer-based materials, cellulose nanocrystals (CNCs) and their magnetic counterparts have attracted significant attention due to their high surface area, abundance of reactive functional groups, renewability, and the added benefit of magnetic separability.

The integration of magnetic nanoparticles, such as Fe_3_O_4_, Fe_2_O_3_, or metallic Fe with CNCs not only enables facile recovery of the adsorbent using an external magnetic field but also enhances adsorption performance through synergistic interactions between the cellulose matrix and the magnetic phase. These magnetic CNC composites, along with related cellulose derivatives such as nanofibrillated cellulose (CNF) and carboxymethyl cellulose (CMC), have demonstrated promising performance for the removal of heavy metal ions, including Pb(II), Cr(VI), Cu(II), and Cd(II), from contaminated water [289,290,291].

In a recent study, Xiong et al. (2025) developed an amino-functionalized magnetic cellulose nanocrystal adsorbent (Fe_3_O_4_@CNC@TEPA) for efficient and selective removal of Cr(VI) from complex electroplating effluents [289]. The material was synthesized by grafting tetraethylenepentamine (TEPA) onto Fe_3_O_4_–CNC using epichlorohydrin as a crosslinker. The positively charged amino groups enable rapid and selective Cr(VI) adsorption even in the presence of competing ions. Characterization and density functional theory (DFT) analysis revealed that electrostatic attraction, redox reactions, and complexation contributed to high adsorption performance. In contrast to this single-target adsorption strategy, hybrid nanocomposite architectures have also been explored for emerging organic pollutants by Pooresmaeil et al. They synthesized a CNC-based nanocomposite (CNCs/MOF(Zn-Co)/MG) by decorating a Zn–Co bimetallic metal–organic framework (MOF) onto cellulose nanocrystals combined with magnetic graphene (MG) oxide for amoxicillin (AMX) removal from water [290]. The material exhibited a magnetic saturation of 22.79 emu g^−1^, and a mean pore diameter of ~6.19 nm, achieving 57.22% removal efficiency under optimal conditions (100 mg/L AMX, pH 7, 5 h, 60 mg adsorbent). Adsorption followed the Freundlich isotherm and pseudo-first-order kinetics, and the nanocomposite maintained acceptable removal efficiency over five reuse cycles [290].

Expanding further into mineral–biopolymer hybrid systems, Zou et al. (2025) reported a magnetic bentonite–nanocellulose crystal composite (CMB) for the removal of rare earth elements, particularly La(III), from wastewater [291]. The integration of nanocellulose onto magnetic bentonite increased the BET surface area from 54.96 to 114.07 m^2^ g^−1^ and significantly enhanced adsorption performance, achieving a maximum La(III) removal of 97.52% under optimal conditions (pH 6, 303 K, 0.9 g/L adsorbent, 360 min). Adsorption followed the Langmuir isotherm and pseudo-second-order kinetics, indicating a chemisorption process influenced by intraparticle and liquid film diffusion, and the composite showed excellent reusability, maintaining 68.18% removal after seven sorption–desorption cycles [291]. Similarly, extending the application scope to organic dye pollutants, Fındık (2025) developed a magnetic nanocomposite (m-CNC-Z) combining zeolite and cellulose nanocrystals for the removal of cationic dyes, methylene blue (MB) and methyl violet 2B (MV-2B) [292]. The material exhibited removal efficiencies of 70.6% for MB and 81.9% for MV-2B under optimal conditions, with adsorption following pseudo-second-order kinetics and the Langmuir isotherm, and thermodynamic analysis indicated spontaneous and exothermic processes [292].

From a broader perspective on alternative iron-based remediation systems, Verma et al. (2025) reviewed the use of pristine and modified zero-valent iron (ZVI) systems, including nanoscale ZVI, doped variants, bio-stabilized composites, and ZVI supported on materials like MXene and nanocellulose, for the remediation of toxic metal ions such as As, Hg, Cr, and Ni from water [293]. The study highlighted that modified ZVI composites offer enhanced stability, selectivity, and reusability, though challenges such as nanoparticle passivation, limited field data, and byproduct toxicity remained, emphasizing the need for further research on sustainable and scalable groundwater treatment [293].

Earlier work also demonstrated the potential of MCNC nanocomposites for heavy metal remediation. For example, Mahlaule-Glory et al. (2024) synthesized magnetic cellulose nanocrystal derived from maize stalks for Pb(II) removal from wastewater, achieving approximately 97% removal under optimized conditions (60 mg adsorbent, 10 ppm Pb(II), pH 6, 60 °C, 5 min) with an adsorption capacity of 47 mg g^−1^, with the MCNC showing reusability for four cycles and effective, though lower, removal in real wastewater samples (53%) [294]. Similarly, Jagirani et al. (2021) developed a solid-phase microextraction (SPME) method using magnetic cellulose nanoparticles (Cell-MNPs) for selective and sensitive extraction of trace Pb(II) from environmental samples [295]. The Cell-MNPs demonstrated a limit of detection of 8.9 µg/L, and were successfully applied to Pb(II) extraction from water and tea samples [295].

Soliman et al. (2023) synthesized magnetic cellulose nanocrystals through a one-pot hydrothermal process using microcrystalline cellulose and Fe_3_O_4_ precursors, resulting in particles sized below 400 nm (TEM) and 20 nm (DLS) [181]. Subsequent modification with chloroacetic acid, chlorosulfonic acid, or iodobenzene introduced carboxylate, sulfonate, and phenyl functionalities, which improved doxycycline hyclate adsorption by minimizing electrostatic repulsion, although the treatments slightly reduced the composite’s crystallinity and thermal stability. In addition to magnetic CNC, other cellulose derivatives, such as CNF [296], carboxymethyl cellulose [297], and carboxylated cellulose [298], have also been combined with magnetic nanoparticles the adsorption of various metal ions [277,299].

#### 6.1.2. Dye Removal

A promising approach to combat water pollution is the integration of hydrogels with nanomaterials to selectively remove pollutants and microorganisms. Hydrogels are hydrophilic polymer networks capable of retaining large volumes of water, offering high porosity, extensive surface area, and tunable chemical functionality for targeted pollutant capture [300,301]. In particular, magnetic nanoparticles and magnetic cellulose nanocrystal (MCNC) composites have been widely used into hydrogels to produce magnetically separable systems for efficient dye removal from water [300,302,303].

**Figure 7:**
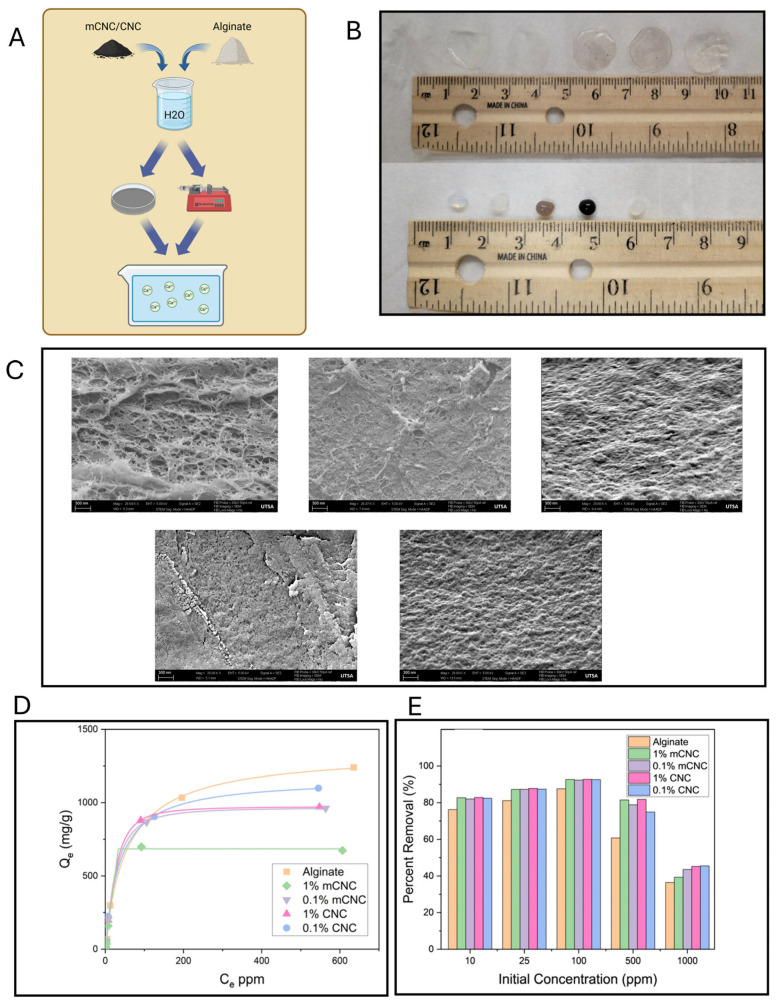
(**A**) Scheme for the sample preparation of hydrogels; (**B**) (left to right, **top** row) thin films of hydrogels loaded with 0.1% CNC, 1% CNC, 0.1% mCNC, 1% mCNC, and pure alginate loaded hydrogel, and (**bottom** row) hydrogel beads (in the same order as thin films); (**C**) Cross-sectional SEM images of the dried hydrogels. (left to right; **top**) pure hydrogel, hydrogel loaded with 0.1% CNC, with 1% CNC, (left to right; **bottom**) hydrogel loaded with 0.1% mCNC, 1% mCNC. (**D**) Equilibrium adsorption capacity; (**E**) percent removal of MB for the alginate hydrogel. Reprinted (adapted) with permission from Moss et al., ACS Appl. Eng. Mater., 2025. Copyright © 2025 American Chemical Society [300].

In a recent study, Moss et al. (2025) developed magnetically responsive alginate hydrogels reinforced with MCNCs for sustainable water purification (Figure 7) [300]. Figure 7A shows the schematic representation of the hydrogel preparation process, while Figure 7B illustrates thin-film hydrogels and hydrogel beads loaded with CNCs or MCNCs at varying concentrations. Cross-sectional SEM images (Figure 7C) reveal that increasing CNC or MCNC content leads to reduced pore size. The MCNC–alginate hydrogels exhibited superparamagnetic behavior (9.85 emu/g at 1% MCNC loading) and enhanced mechanical strength compared to pure alginate. Although pure alginate demonstrated the highest methylene blue adsorption capacity (1357 mg/g) (Figure 7D), MCNC-loaded hydrogels achieved superior removal efficiency at intermediate concentrations, likely due to improved diffusion and enhanced surface interactions (Figure 7E). Overall, these MCNC-alginate hydrogels exhibited magnetic recoverability, mechanical robustness, and biobased sustainability, making them promising candidates for advanced water purification applications [300].

Extending this strategy toward hybrid systems, Peighambardoust et al. (2025) synthesized a magnetic nanocomposite hydrogel by grafting acrylamide onto carboxymethyl cellulose (CMC) and incorporating biochar (CL) and magnetic biochar (CL–Fe_3_O_4_) to enhance dye adsorption efficiency [304]. The resulting material demonstrated high removal efficiencies for methylene blue (MB) and methyl violet (MV), reaching up to 95% under optimal conditions. Adsorption followed the Langmuir isotherm and pseudo-second-order kinetics, indicating monolayer adsorption and chemisorption mechanisms. Thermodynamic analysis further revealed that the process was spontaneous and exothermic, confirming that the CMC-g-poly(AAm)/CL–Fe_3_O_4_ hydrogel is a promising candidate for wastewater treatment and dye removal applications [304].

In comparison to these recent systems, earlier work by Wu et al. (2021) synthesized a magnetic polysaccharide composite hydrogel composed of sodium alginate (SA) and CMC embedded with in situ Fe_3_O_4_ nanoparticles for efficient heavy metal ion removal [303]. The hydrogel exhibited maximum adsorption capacities of 71.83 mg/g for Mn(II), 89.49 mg/g for Pb(II), and 105.93 mg/g for Cu(II), with adsorption governed by ion exchange and chemisorption mechanisms [303]. Furthermore, Moharrami et al. (2020) developed a sustainable hydrogel nanocomposite by integrating magnetite-functionalized cellulose nanocrystals, derived from sugar beet pulp, into a starch-g-AMPS-co-AA (2-acrylamido-2-methylpropanesulfonic acid–co–acrylic acid) hydrogel matrix [305]. In this system, Fe_3_O_4_ nanoparticles were anchored onto cellulose nanocrystals to form MCNCs, which acted as nanofillers to enhance adsorption efficiency. The resulting nanocomposite demonstrated exceptionally high adsorption capacities for crystal violet (2500 mg/g) and methylene blue (1428.6 mg/g), attributed to strong electrostatic interactions and the abundance of active sites. In addition, the material exhibited excellent reusability and dye selectivity, positioning it as an effective and eco-friendly adsorbent for wastewater purification [305].

Following a comparable MCNC-based reinforcement approach, Singh et al. (2022) synthesized a magnetic hydrogel nanocomposite by embedding Fe_3_O_4_ nanoparticles within a cellulose nanocrystal (CNC)–polyacrylamide matrix to improve both adsorption performance and magnetic recoverability [306]. The composite displayed strong structural stability, superparamagnetic behavior, and high surface activity, enabling efficient removal of heavy metal ions such as Pb(II) and Cd(II) from aqueous solutions [306].

Beyond CNC-based systems, other cellulose derivatives—including cellulose nanofibrils (CNF), carboxymethyl cellulose, and carboxylated cellulose—functionalized with magnetic nanoparticles have also been widely studied for dye removal [307,308,309,310,311,312,313].

#### 6.1.3. Pickering Emulsions and Oil–Water Separation

Oil–water separation is critical for environmental protection, particularly in the treatment of industrial wastewater and remediation of oil spills. Conventional separation methods often suffer from limitations such as low efficiency, membrane fouling, and poor recyclability. In this context, hydrophilic/oleophobic magnetic cellulose-based membranes have gained increasing attention due to their renewable nature, magnetic responsiveness, and reusability. In addition, MCNCs can act as effective stabilizers for Pickering emulsions, forming robust interfacial barriers that enable selective, efficient, and magnetically recoverable oil–water separation [174,175,176].

For example, Hasan et al. (2021) synthesized superparamagnetic Fe_3_O_4_-coated cellulose nanocrystals (CNCs) via a one-step coprecipitation method, systematically varying the CNC:Fe_3_O_4_ ratio to optimize magnetic and emulsifying properties [174]. The resulting MCNCs effectively stabilized castor oil–water Pickering emulsions, exhibiting high saturation magnetizations (56–60 emu/g), and enabling magnetically controlled demulsification and nanoparticle recovery. These magnetic CNCs show promising applications in magnetically driven separation and oil recovery processes [174]. Extending this approach toward multiphase systems, in a follow-up study, they developed a novel castor oil/water/ethanol Pickering emulsion stabilized by Fe_3_O_4_-coated cellulose nanocrystals and lignin-coated Fe_3_O_4_ nanoparticles [175]. This system enabled magnetically controlled demulsification for ethanol extraction, with tunable emulsion stability, efficient phase separation, and excellent nanoparticle recyclability over multiple cycles [175].

Furthermore, Mikhaylov et al. (2021) investigated oil-in-water Pickering emulsions stabilized by magnetite/CNC hybrids, demonstrating that emulsions containing 37–83 wt% magnetite exhibited superior stability due to coordination interactions between CNCs and magnetite within the electrical double layer [314]. These emulsions showed enhanced viscosity, pronounced thixotropic behavior, and improved Cr(VI) adsorption capacity compared to systems stabilized by CNCs or Fe_3_O_4_ alone [314].

More recently, with a focus on practical separation performance, Amiri et al. (2024) synthesized recyclable MCNCs from cotton for the efficient demulsification of water-in-crude oil emulsions [270]. The MCNCs achieved 100% demulsification efficiency at 50 °C without a magnet and 90% at 20 °C under an applied magnetic field, outperforming standalone Fe_3_O_4_ nanoparticles. The materials also maintained performance over four reuse cycles. Mechanistic analysis indicated that MCNCs reduced interfacial tension between oil and water, enabling rapid separation through combined chemical and magnetic effects [270].

Parallel to these particle-stabilized systems, membrane-based strategies have also emerged, as demonstrated by Yao et al. (2025), who developed a photo-induced antifouling Janus micro/nano-paper based on multiscale cellulose and lignin nanoparticles for highly efficient oil–water emulsion separation [315]. The membrane featured a “hydrophilic–amphiphilic–hydrophobic” gradient structure, along with a stable granular network and optimized porosity, achieving separation efficiencies exceeding 98–99%. Its excellent antifouling properties and durability over multiple cycles underscore its potential for oil spill remediation and fluid transport applications [315].

Overall, across these studies, CNCs and other cellulose derivatives functionalized with magnetic nanoparticles consistently demonstrate strong performance on stabilizing Pickering emulsions, enabling magnetic demulsification, and facilitating efficient oil–water separation, although system design (emulsion vs. membrane) and composition significantly influence separation efficiency and recyclability [316,317,318,319].

#### 6.1.4. Other Environmental Applications: Bio-Adsorbents and Flocculants

MCNC composites have demonstrated significant potential across a range of environmental remediation applications. In particular, they serve as efficient adsorbents for arsenic and phosphate removal, contributing to the mitigation of toxic metal contamination and nutrient pollution from agricultural runoff [320,321]. As an early example of such systems, Yu et al. (2013) synthesized cellulose@Fe_2_O_3_ nanocomposites via a one-step green co-precipitation method in a NaOH–thiourea–urea system, where cellulose served as a low-cost template for nanoparticle growth [321]. The resulting composites exhibited uniform Fe_2_O_3_ nanoparticle dispersion and strong magnetic responsiveness, enabling facile separation. Notably, they achieved high arsenic adsorption capacities of 23.16 mg g^−1^ for As(III) and 32.11 mg g^−1^ for As(V), outperforming many previously reported magnetic adsorbents [321]. Extending this templating strategy toward nutrient removal, Liang et al. (2017) prepared mesoporous α-Fe_2_O_3_ using CNCs as a template and evaluated its phosphate adsorption performance [320]. The material exhibited a well-defined mesoporous structure with a specific surface area of 106.9 m^2^ g^−1^ and a pore volume of 0.4984 cm^3^ g^−1^. Adsorption studies indicated that phosphate removal followed the Freundlich isotherm and pseudo-second-order kinetic model, with performance influenced by parameters such as initial concentration, contact time, pH, and ionic strength [320].

Beyond adsorption-based approaches,, MCNC-based systems have also shown promise as magnetic flocculants for wastewater treatment, enabling rapid aggregation and magnetic separation of suspended solids while maintaining reusability [322,323]. In parallel with these developments, when integrated with photocatalytic materials such as TiO_2_, ZnO, or g-C_3_N_4_, magnetic CNCs serve as effective catalyst supports for the visible-light-driven degradation of dyes and pharmaceutical contaminants [324]. Similarly, from a biosorption perspective, biopolymer–magnetite hybrid systems based on CNCs function as magnetically separable biosorbents, facilitating rapid adsorption and straightforward recovery of a wide range of pollutants from aqueous environments [325].

### 6.2. Catalysis and Green Chemistry

MCNCs have emerged as versatile materials in catalysis and green chemistry due to their unique combination of high surface area, abundant functional groups, and facile magnetic separation. These attributes make them excellent support and, in some cases, active components in catalytic systems, enabling efficient transformations while promoting catalyst recovery, recyclability, and overall process sustainability.

#### 6.2.1. Heterogeneous Catalysis

MCNC-based materials have gained significant attention as supports for heterogeneous catalysis, as their surface functionality enables the effective anchoring of metal nanoparticles while their magnetic properties facilitate straightforward recovery and reuse [287,326,327,328,329]. This combination enhances catalytic efficiency and selectivity while reducing downstream processing requirements.

For example, Khalilzadeh (2020) developed a Fe_3_O_4_@cellulose nanocrystal/Cu nanocomposite sensor using a green synthesis route with *Petasites hybridus* leaf extract as a reducing and stabilizing agent [287]. The sensor demonstrated excellent electrochemical performance, with a wide linear detection range (0.05–600 μM), a low detection limit (0.01 μM), and reliable quantification of venlafaxine in complex matrices such as urine, water, and pharmaceutical samples [287].

Similarly, Pandya et al. (2024) used a Fe_3_O_4_@microcrystalline cellulose nanocatalyst for the synthesis of 2,3′-biindole derivatives, highlighting its efficiency, cost-effectiveness, and reusability [329]. The magnetic catalyst enabled easy separation, simplified purification, and achieved high yields of 78–93%, making the process both practical and sustainable. The magnetic catalyst enabled facile separation and simplified purification, demonstrating strong alignment with green chemistry principles, including improved atom economy and reduced environmental impact [329].

Moving toward noble metal-supported systems with enhanced activity, Xu et al. (2022) further advanced this field by developing recyclable palladium (Pd)-based catalysts through the immobilization of Pd onto melamine-formaldehyde (MF)-modified and dopamine-coated CNC, integrated with magnetic nanoparticle clusters [330]. These catalysts exhibited high activity in the reduction of 4-nitrophenol (4-NP) with kinetics well described by the Langmuir–Hinshelwood model. Notably, the optimized catalyst (MNP2-Pd-MC) maintained high catalytic performance over seven reuse cycles, demonstrating the effectiveness of magnetic templating strategies in designing durable nanocatalysts [330].

In the context of environmental catalysis, Zhan et al. (2018) synthesized MnFe_2_O_4_/CNC nanocomposites for the degradation of methylene blue (MB) [331]. The enhanced performance was attributed to increased surface area, reduced particle size, and a lower band gap, which collectively promoted the generation of hydroxyl radicals from H_2_O_2_. Importantly, the MnFe_2_O_4_/CNC composites—particularly those with 20 wt% CNC—achieved 99% MB degradation, outperforming bare MnFe_2_O_4_ by over 60%. In addition, their strong magnetic properties enabled excellent recyclability, highlighting their potential for treating environmental pollutants [331].

**Figure 8:**
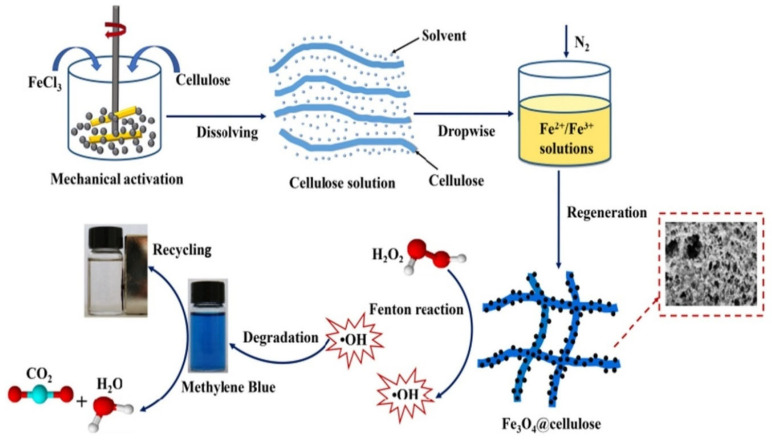
Graphical representation of the preparation of Fe_3_O_4_@cellulose nanoconjugates and their catalytic activity. Reproduced from Lu et al., Nanomaterials 2019, licensed under CC BY 4.0 [332].

Similarly, focusing on Fenton-type catalytic systems, Lu et al. (2019) developed a stable Fe_3_O_4_@cellulose heterogeneous Fenton catalyst via a novel in situ chemical co-precipitation method. By using mechanically activated (MA) + FeCl_3_-pretreated cellulose (MAFCC)—which was more soluble and uniformly distributed than standard MA cellulose—they provided abundant reactive sites for the in situ growth of Fe_3_O_4_ nanoparticles [332]. Consequently, the resulting Fe_3_O_4_@MAFCC nanocomposite exhibited superior catalytic activity for the degradation of methylene blue compared to both Fe_3_O_4_@MAC and bare Fe_3_O_4_ nanoparticles (Figure 8). Moreover, the strong interaction between MAFCC and Fe_3_O_4_ ensured structural stability and excellent reusability across ten cycles, offering a green approach to fabricating efficient catalysts for organic pollutant treatment [332].

Further advancing toward hybrid core–shell catalytic architectures,, Zhang et al. (2020) reported a facile synthesis for cellulose nanocrystal (CNC)-supported magnetic CuFe_2_O_4_@Ag@ZIF-8 nanospheres, featuring a paramagnetic CuFe_2_O_4_@Ag core and a porous ZIF-8 (Zeolitic Imidazolate Framework-8) shell [328]. Due to the synergistic integration of ZIF-8 and Ag components, these nanocomposites demonstrated significantly higher catalytic activity than CuFe_2_O_4_@Ag alone. Ultimately, this approach provides a robust new strategy for fabricating CNC-supported magnetic core–shell catalysts with broad applicability in heterogeneous catalysis, biocatalysis, and environmental remediation [328].

#### 6.2.2. Enzyme Immobilization

MCNCs have emerged as promising support for enzyme immobilization, offering enhanced stability, ease of separation, and excellent reusability. Their high surface area, abundant functional groups, and tunable magnetic properties facilitate efficient enzyme attachment, thereby improving overall catalytic performance and operational efficiency [280,333,334,335,336].

In this context, Yang et al. (2025) synthesized a novel magnetic nanocellulose carrier via low-eutectic solvent treatment, amine modification, and metal hybridization to immobilize ω-transaminase [334]. Compared to the free enzyme, the immobilized variant exhibited enhanced catalytic stability, expanded optimal operating conditions (pH 9.0 and 30 °C), superior thermal stability, and retained over 80% activity after ten reuse cycles. Similarly, targeting industrial applications, Yu et al. (2025) derived magnetic cellulose microparticles (MC@Fe_3_O_4_) from inexpensive filter paper fibers to immobilize laccase [333]. After optimizing the system using a Box–Behnken design, the immobilized laccase demonstrated exceptional pH, thermal, and storage stability, successfully sustaining bisphenol A (BPA) degradation across 8 recovery cycles [334].

In the context of food safety, Zhang et al. (2024) developed a multifunctional magnetic dopamine (DA) and polyethyleneimine (PEI) CNC nanocarrier (DA/PEI@Fe_3_O_4_/CNCs) to immobilize aldo-keto reductase for the efficient degradation of patulin (PAT) in fruit products [335]. This composite achieved near-complete detoxification of patulin (PAT) in pear juice (98%) and buffer solutions (100%) while resisting proteolysis and preserving juice quality. Earlier studies further support the versatility of MCNC platforms, as demonstrated by Ariaeenejad et al. (2021), who utilized CNCs isolated from sugar beet pulp, functionalizing them with magnetite nanoparticles and DA to covalently immobilize hydrolytic enzyme cocktails (DA/Fe_3_O_4_NPs@CNCs) [337]. This platform effectively suppressed enzyme leaching, shifted the optimum pH toward alkaline conditions, and enabled easy recovery, with over 50% activity retained after 10 cycles. Finally, demonstrating the broad versatility of these supports, Cao et al. (2014) prepared a biocompatible MCNC composite via co-precipitation and electrostatic self-assembly to immobilize papain through formaldehyde activation [336]. The MCNC-supported papain exhibited superior solvent tolerance and maintained over 90% relative activity at elevated temperatures (50–70 °C), confirming MCNCs as highly effective platforms for enzyme immobilization [336].

### 6.3. Biological and Biomedical Applications

With the rapid advancement of nanotechnology, MCNCs have emerged as an exciting class of bio-nanomaterials. They combine the natural advantages of cellulose—such as biocompatibility, biodegradability, and sustainability—with the magnetic properties of nanoparticles like Fe_3_O_4_ or γ-Fe_2_O_3_. This unique combination has opened promising opportunities in biomedical science, where precise control, targeted action, and easy recovery are essential. The abundant hydroxyl groups on CNCs provide versatile sites for chemical modification, enabling the attachment of drugs, enzymes, or targeting molecules, while the magnetic core enables external magnetic control, imaging, and separation [338,339]. Consequently, MCNCs have demonstrated strong potential across a variety of applications, including targeted drug delivery, magnetic hyperthermia for cancer treatment, tissue engineering, and biosensing. Their multifunctional and responsive nature makes them ideal candidates for future theranostic systems that integrate diagnosis and therapy within an eco-friendly, cellulose-based platform.

#### 6.3.1. Drug Delivery

**Figure 9:**
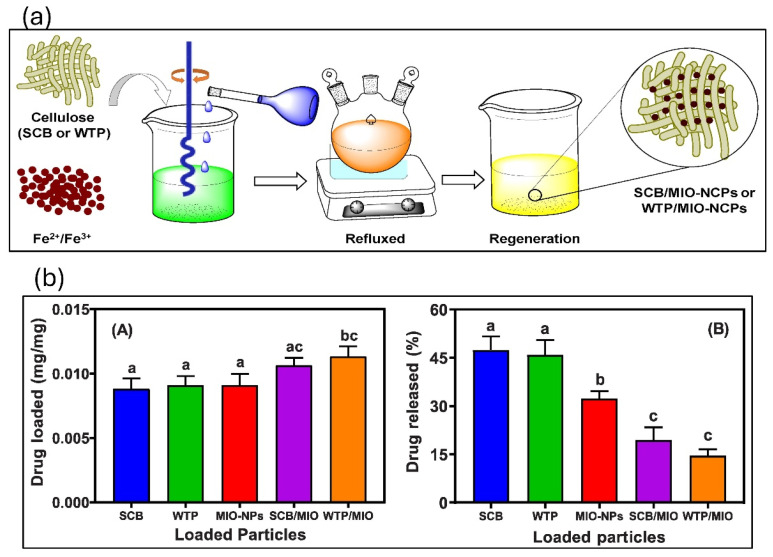
(**a**) Preparation of polymer-supported magnetic iron oxide nanoparticles incorporated into cellulose matrices; (**b**) Amount of drug-loading (**A**) drug-releasing (**B**) of cellulose (SCB and WTP), MIO-NPs, SCB/MIO-NCPs, and WTP/MIO-NCPs. Similar letters above bars indicate no significant differences at *p*-value ≤ 0.05. Reproduced from Naznin et al., Pharmaceutics, 2023, licensed under CC BY 4.0 [339].

Both CNCs [8,340,341] and magnetic nanoparticles [342,343] are well established in drug delivery. By combining these materials, MCNC composites offer a highly effective platform for controlled, magnetically targeted therapeutic release [344]. For example, Naznin et al. (2023) developed magnetic iron oxide nanoparticle (MIO-NP)-incorporated nanocomposite particles (NCPs) via an in situ co-precipitation method, utilizing waste tissue paper (WTP) and sugarcane bagasse (SCB) as sustainable cellulose sources [339]. Structural analyses (FESEM, XRD) revealed irregularly spherical, agglomerated particles (10–12 nm crystallite size), while VSM confirmed their paramagnetic nature. The composites exhibited low antioxidant activity but significantly enhanced swelling capacities, over twice that of the pure cellulose samples. When loaded with metronidazole, the WTP/MIO-NCPs achieved the highest loading efficiency and the most sustained release profile among the tested materials (Figure 9). highlighting the viability of waste-derived biomass for cost-effective, magnetic-assisted drug delivery [339].

Similarly, focusing on emulsion-based delivery systems, Low et al. (2019) formulated Fe_3_O_4_@cellulose nanocrystal (MCNC)-stabilized Pickering emulsions for the magnetically triggered delivery of curcumin (CUR) [345]. These emulsions achieved a remarkable loading efficiency (~99%) and released over 50% of the drug within four days under an external magnetic field. In vitro studies confirmed significant growth inhibition of human colon cancer cells and reduced 3-D multicellular spheroid volume, all while remaining non-toxic to brine shrimp [345]. Extending MCNC applications to hydrogel-based delivery platforms, Supramaniam et al. (2018) incorporated rice husk-derived MCNCs into alginate hydrogel beads [346]. Using ibuprofen as a model drug, the structurally enhanced beads demonstrated excellent magnetic responsiveness and controlled release behavior, fitting well with Korsmeyer-Peppas and Peppas-Sahlin models for sustained delivery [346].

#### 6.3.2. Magnetic Hyperthermia Treatment

Magnetic nanoparticles have attracted significant attention for magnetic imaging-guided hyperthermia, where localized heating under an alternating magnetic field is used to selectively destroy tumor cells [347,348,349]. Conjugating these nanoparticles with CNCs provides a highly biocompatible and biodegradable matrix with functional surface sites for targeting molecules [350].

Recent studies highlight the efficiency of this approach. For example, Hasan et al. (2026) demonstrated that magnetic cellulose nanocrystal (MCNC) composites synthesized from sulfated CNC and TEMPO-oxidized CNC at various CNC-to-magnetite loadings are promising biocompatible platforms for magnetic hyperthermia, where the sulfated CNC-based composite (S-CNC/Fe_3_O_4_, 1:2) exhibited the highest specific absorption rate (649 W/g-Fe_3_O_4_) under a 30.4 kA.m^−1^ magnetic field, indicating superior heating efficiency [206]. The study further establishes that interfacial chemistry and Fe_3_O_4_ loading critically govern magnetic heating performance, making these MCNC systems highly suitable for hyperthermia applications [206].

In comparison, hydrogel-based magnetic systems also demonstrate effective hyperthermia performance, as reported by Ahmadpour et al. (2024), who synthesized a novel superparamagnetic nanobiocomposite using a cross-linked pectin–cellulose hydrogel matrix magnetized with Fe_3_O_4_ nanoparticles [215]. Displaying excellent thermal stability and a saturation magnetization of 48.8 emu/g, the composite achieved a high specific absorption rate (SAR) of 126 W g^−1^ at just 0.5 mg mL^−1^ under alternating magnetic fields [215]. Similarly, multifunctional hybrid systems have been explored to enhance therapeutic performance, as demonstrated by Eivazzadeh-Keihan et al. (2022), who developed a potent magnetic nanobiocomposite by cross-linking carboxymethyl cellulose (CMC) with epichlorohydrin, modified with silk fibroin (SF), halloysite nanotubes (HNTs), and in situ Fe_3_O_4_ [351]. The material was highly biocompatible with healthy cells but demonstrated targeted anticancer activity against BT549 breast cancer cells, achieving a maximum SAR of 67 W g^−1^ (at 1 mg mL^−1^ under a 400 kHz field) [351].

Earlier studies further established the feasibility of magnetically responsive hydrogel systems, as Wang et al. (2017) developed an injectable, thermally contractible hydrogel composed of hydroxypropyl methylcellulose (HPMC), polyvinyl alcohol (PVA), and Fe_3_O_4_ to safely ablate tumors in vivo while protecting surrounding healthy tissues during induction heating [350].

#### 6.3.3. Tissue Engineering

Traditional tissue engineering materials often face limitations in terms of degradation and biofunctionality [352]. In contrast, MCNCs provide a renewable, mechanically robust alternative that supports cell adhesion while enabling magnetic field-guided cell alignment and magneto-mechanical stimulation [353,354,355,356].

For instance, Pastrana et al. (2016) designed magnetite-reinforced bacterial nanocellulose (Fe_3_O_4_-BNC) nanofibers that exhibited smaller fiber diameters and larger pore areas than pure BNC [357]. The composite demonstrated strong potential for magnetically guided cell targeting, and when coated with PEG, yielded high cell viability (96%) [357]. More recently, advanced fabrication strategies have further improved scaffold precision and functionality, as reported by Iglesias-Mejuto et al. (2024), who utilized a combination of 3D printing and supercritical CO_2_ technologies to fabricate nanostructured cellulose aerogels incorporating superparamagnetic iron oxide nanoparticles [358]. These highly precise, hierarchically porous scaffolds proved safe and hemocompatible in ovo and in vivo, making them ideal for bone tissue engineering [358]. Additionally, electrospinning approaches have been employed to mimic extracellular matrix architectures, as Mousa et al. (2021) fabricated iron-doped cellulose acetate (CA) nanofiber mats to mimic the extracellular matrix (ECM) for bone tissue engineering [359]. The incorporation of just 0.5 wt.% iron acetate produced highly dense, thermally stable nanofibers that rapidly promoted biomineralization and supported strong adhesion of human fetal osteoblast cells [359].

#### 6.3.4. Wound Healing

MCNCs are increasingly being incorporated into hydrogels and smart dressings to create scaffolds that actively support cell migration and tissue regeneration [360]. Their magnetic properties can be leveraged for on-demand drug release or localized hyperthermia to reduce infection rates and accelerate healing.

Highlighting the potential of “smart” responsive dressings, Williams et al. (2019) integrated highly magnetized CoFe_2_O_4_ nanorods onto cellulose fibers to enable the wireless monitoring of wound healing [361]. The resulting magnetic cellulose dressing can respond to temperature changes at the wound site, communicating critical healing data and reducing the need for unnecessary, disruptive dressing changes. Furthermore, focusing on bioactive and antibacterial systems, Moniri et al. (2018) synthesized an eco-friendly Fe_3_O_4_/BNC nanocomposite film using green-synthesized magnetic nanoparticles derived from Aloe vera extract [362]. These non-toxic films promoted human dermal fibroblast healing within 48 h and exhibited strong antibacterial activity against pathogens like *S. aureus*, *S. epidermidis*, and *P. aeruginosa*. The films positively modulated the expression of key healing-related genes (including TGF-B1 and various microRNAs), demonstrating their bioactivity as advanced wound dressings [362].

#### 6.3.5. Bioimaging, Biosensing, and Gene Delivery

Beyond the applications detailed above, MCNCs show significant promise across a broad spectrum of advanced biomedical technologies. In bioimaging, they can serve as highly effective contrast agents for magnetic resonance imaging (MRI), providing enhanced tissue visualization [363,364,365]. In biosensing, their functionalized surfaces allow for the highly sensitive and selective detection of specific analytes [366]. Additionally, the inherent or chemically modified surfaces of MCNCs can exhibit distinct antimicrobial properties to prevent clinical infections. Finally, as magnetic nanocarriers, MCNCs offer a reliable vehicle for gene delivery, enabling the targeted transport of DNA or RNA via external magnetic guidance to improve the precision of genetic therapies [367].

### 6.4. Agricultural Applications: Pesticide Adsorption and Soil Remediation

MCNCs offer promising applications in agriculture due to their biocompatibility, high surface area, and magnetic responsiveness. They serve as excellent carriers for the controlled delivery of fertilizers, pesticides, or plant growth regulators. By enabling precise, targeted release under external magnetic guidance, MCNCs can significantly reduce chemical runoff and environmental contamination [368,369,370]. Furthermore, these nanocomposites can effectively capture and degrade organophosphate and chlorinated pesticides through adsorption, redox, and catalytic mechanisms, offering an eco-friendly approach for removing toxic agrochemicals from contaminated water sources.

Demonstrating their effectiveness in analytical and separation-based agrochemical removal, Yi et al. (2019) developed a magnetic partially carbonized cellulose nanocrystal (MPC-CNC) composite by loading Fe_3_O_4_ nanoparticles onto acid-treated microcrystalline cellulose [371]. Designed for the magnetic solid-phase extraction of triazine and triazole pesticides from water, the optimized UHPLC-MS/MS method demonstrated high sensitivity, excellent recovery rates (73.7–117.1%), and detection limits as low as 2.2–6.1 ng L^−1^, confirming MPC-CNC as a highly effective and reusable adsorbent [371]. Building on this concept, Jafari et al. (2023) synthesized a novel magnetic carbonized cellulose composite functionalized with a MIL-101 metal–organic framework [372]. This material demonstrated excellent sensitivity, linearity, and precision for the solid-phase extraction of organophosphorus pesticides from real food and water samples. More recently, focusing on multifunctional pollutant removal systems, Markeb et al. developed a multifunctional chitosan/carboxymethyl cellulose-supported zero-valent iron nanocomposite (CS@nZVI-CMC NC) with high surface area (127.95 m^2^/g) and nanoscale morphology (~8 nm). This composite demonstrated exceptional simultaneous removal efficiencies for multiple heavy metals (up to 96.9%) and pesticides (up to 98.5%) from water [373].

Beyond water purification, MCNCs can be incorporated into soil amendments to enhance nutrient retention and water-holding capacity. Their magnetic properties allow for easy recovery and recycling from the soil matrix, minimizing waste and improving agricultural sustainability [374,375]. Demonstrating this potential, Bi et al. (2025) developed a polyethyleneimine (PEI) cross-linked magnetic cellulose aerogel (MCA_PEI_) to remediate cadmium (Cd)-polluted soil and mitigate Cd stress on green fertilizers [374]. The MCA_PEI_ exhibited rapid Cd(II) uptake, broad pH adaptability, and a maximum binding capacity of 200.41 mg g^−1^. Under optimal conditions, the easily recoverable magnetic aerogel reduced DTPA-extractable Cd by 89%. After applying and recycling just 1% MCA_PEI_ into the soil, Cd accumulation in the fertilizer decreased significantly, while overall plant growth and soil properties improved, demonstrating a highly sustainable strategy for heavy metal remediation [374].

### 6.5. Energy Storage and Conversion

MCNCs possess great potential for energy storage and conversion applications due to their excellent mechanical properties and tunable conductivity. They are increasingly being used as electrode materials in supercapacitors, lithium-ion batteries, and flexible energy storage devices, where their structural integrity and high surface area significantly enhance performance [376,377].

For instance, demonstrating hybrid energy harvesting–storage concepts, Abdalkarim et al. (2021) developed dipole-responsive, magnetic/solar-driven phase change fiber (PCF) composites reinforced with MCNCs to improve thermal energy storage for agricultural heating [377]. The optimal PCF/MCNC-5% composite exhibited strong magnetic properties (saturation magnetization of 1.3 emu g^−1^) and high latent heat enthalpies (69.2–83.1 J g^−1^), enabling efficient solid–liquid phase change energy storage. Crucially, the material demonstrated high magnetic-to-thermal (32.5%) and solar-accelerated (58.5%) energy storage efficiencies, making it a highly promising material for drying and preserving agricultural products [377].

Beyond energy storage, MCNCs contribute to electromagnetic interference (EMI) shielding, providing lightweight, flexible materials capable of absorbing unwanted electromagnetic radiation [378]. Magnetically conductive composites, such as CNC–Fe_3_O_4_–polymer films, are also utilized in the synthesis of conductive paper [379]. Liu et al. (2015) successfully synthesized well-dispersed Fe_3_O_4_ nanoparticles using a co-precipitation method with CNCs as a template, preventing nanoparticle aggregation and ensuring uniform distribution across the cellulose network [379]. FTIR analysis confirmed that the nanoparticles were immobilized via hydroxyl group interactions. The resulting Fe_3_O_4_/CNC-coated paper exhibited a conductivity of 0.0269 S/m (at a coating amount of 14.75 g m^−2^), indicating strong potential as an anti-static material for packaging [379].

Overall these multifunctional characteristics—including improved thermoelectric charge transport and magnetically induced actuation—make MCNCs a highly versatile platform for advanced electronic and self-healing technologies [377].

### 6.6. Materials and Structural Applications

Magnetic cellulose-based composites hold great promise for structural and advanced-material applications due to their superior mechanical strength and magneto-responsive behavior. They are frequently used in magnetically responsive hydrogels and films that act as actuators or sensors, capable of deliberate deformation under magnetic fields [380,381,382,383,384]. Additionally, smart coatings based on magnetic CNCs offer excellent corrosion resistance, self-cleaning, and antifouling capabilities [385]. When integrated into magnetic paper and food packaging, these composites significantly improve mechanical durability, antimicrobial performance, and barrier properties [386].

#### 6.6.1. Magnetic Paper

Magnetic paper has diverse applications in information storage, electromagnetic shielding, specialty printing, filtration, and anti-counterfeiting security [18,387]. It can be prepared via the direct addition of magnetic particles, in situ synthesis, fiber coating, or cavity loading [388]. Nanocellulose is an ideal substrate for these applications due to its high mechanical strength and excellent processability.

A primary challenge in formulating magnetic paper is balancing the magnetic loading with the paper whiteness, as high ferromagnetic content naturally darkens the material. To overcome this, researchers often employ a “sandwich” structure, where layers of plain nanocellulose encapsulate a central magnetic layer. For example, demonstrating this structural design strategy, Sriplai et al. (2018) prepared bacterial cellulose sheets containing CoFe_2_O_4_ nanoparticles and sandwiched them between ZnO-loaded layers, successfully producing a white magnetic paper with high reflectance, flexibility, and strong anti-counterfeiting potential [389]. Similarly, adopting a multilayer coating approach, Papaparaskeva et al. (2020) utilized a layer structure with cellulose acetate fibrous membranes impregnated with magnetic nanoparticles to achieve clean white coloring alongside controlled magnetic properties [390]. In contrast to layered architectures, earlier work focused on embedded nanostructures, as Li et al. (2013) fabricated transparent magnetic nanopaper by immobilizing Fe_3_O_4_ nanoparticles within a nanofibrillated cellulose (NFC) network [391]. The resulting material combined high transparency, a strong magnetic response, mechanical strength, and flexibility, making it a promising candidate for magneto-optical applications.

#### 6.6.2. Food Packaging

Nanocellulose-based composites are highly promising for food packaging application because they are inherently non-toxic, mechanically robust, and thermally stable [18,380,392]. Applying a weak magnetic field during fabrication can align the nanocellulose fibers within a polymer matrix, improving filler dispersion and creating a more compact, highly oriented structure [393].

Demonstrating this effect, Li et al. (2022) prepared PVA/CNC and PVA/CNF films featuring magnetically aligned nanocellulose [257]. This alignment successfully reduced surface roughness while enhancing the mechanical, optical, and barrier properties of the films. Consequently, these films provided excellent oxygen shielding, extending the freshness of fruits and proving ideal for sustainable food packaging. In another innovative study, Wang et al. (2023) developed cellulose nanofiber–iron oxide–thyme essential oil (TEO–Fe_3_O_4_–CNF) aerogels featuring a micro-nano porous structure designed for the controlled release of TEO [386]. Fabricated via in situ mineralization and freeze-drying under a magnetic field, the aerogels exhibited excellent antibacterial activity, sustained TEO release, and high retention rates, ultimately extending the shelf life of perishable foods while preserving their sensory qualities [386].

### 6.7. Optical Application

Magnetic nanomaterials possessing both strong magnetic and optical properties have gained significant attention for their use in sensors, optical converters, modulators, and various photonic applications [394]. In these composite films, magnetic strength and transparency heavily depend on the concentration of Fe_3_O_4_ nanoparticles. However, excessive Fe_3_O_4_ loading frequently causes particle clustering, which drastically lowers transparency and limits overall performance.

To overcome this limitation, it is essential to select a suitable substrate that facilitates the even dispersion of nanoparticles [395]. Cellulose has been widely explored as a green, low-cost option for this purpose. In this context, Zhang et al. (2021) synthesized magnetic films made from bamboo-derived nanocellulose and Fe_3_O_4_ [396]. The cellulose matrix enabled a highly uniform nanoparticle distribution, resulting in a film that exhibited both a strong magnetic response and a high light transmittance of up to 94%. Overall, by combining sustainability with top-tier functional performance, such films hold immense promise for the next generation of magneto-optical devices.

## 7. Limitations and Challenges

Although magnetic cellulose nanocrystals (MCNCs) have shown considerable promise in biomedical, environmental, and catalytic applications, several important challenges still limit their large-scale utilization and commercialization. One of the major concerns is the limited understanding of their long-term stability in aquatic and biological environments, especially under different pH levels, ionic strengths, and physiological conditions [397]. While MCNCs are generally regarded as biocompatible materials, excessive loading of magnetic nanoparticles may still lead to cytotoxic effects, oxidative stress, or inflammatory responses in biological systems. In addition, large-scale manufacturing remains challenging due to issues associated with production cost, reproducibility, scalability, and maintaining consistent material quality between batches. Another concern is the possible leaching of iron ions from Fe_3_O_4_-based composites, which could negatively impact both environmental safety and long-term material stability [332,398]. Moreover, photocatalytic applications of MCNCs are often limited by low quantum efficiency and rapid electron–hole recombination [324]. Finally, despite their multifunctional properties, MCNCs must still compete economically with lower-cost adsorbent and magnetic materials, such as magnetic biochar and activated carbon, for widespread industrial adoption.

## 8. Comparison of MCNCs with Competing Functional Materials

MCNCs have been used in a wide range of applications; however, their performance and suitability should be considered within the broader context of emerging functional materials developed for environmental, catalytic, and energy-related applications. Cellulose-based hybrids with metal–organic frameworks (MOFs), MXenes (two-dimensional transition metal carbides/nitrides), magnetic biochars, and graphene are widely reported in the literature [399,400,401,402,403,404,405,406,407,408,409,410,411,412]. These materials often demonstrate comparable or even higher adsorption capacities, enhanced chemical stability, and improved resistance to real-world operating conditions.

In comparison with metal–organic frameworks (MOFs), MCNCs generally exhibit lower specific surface area and adsorption capacity; however, they compensate with excellent biodegradability, high stability in aqueous environments, and significantly lower toxicity, making them more suitable for sustainable and biologically relevant applications. Compared to MXenes and graphene-based materials, MCNCs lack intrinsic electrical conductivity and high charge transport efficiency, but they offer distinct advantages in environmental compatibility, biocompatibility, and facile dispersion in aqueous media without the need for complex surface stabilization. Similarly, while magnetic biochar represents a highly cost-effective and scalable material for large-scale adsorption and environmental remediation, MCNCs provide greater control over surface functionalization, more uniform nanostructure formation, and improved reproducibility in composite fabrication.

Overall, MCNCs represent a unique functional class where sustainability, tunable interfacial chemistry, and magnetic responsiveness are prioritized over extreme physicochemical performance metrics such as very high adsorption capacity or superior electrical conductivity. Consequently, their practical performance is strongly dependent on the specific application, and they should be regarded as complementary materials rather than universally superior alternatives to other advanced functional systems.

## 9. Opportunities and Prospects

While magnetic cellulose-based composites have found applications across diverse fields, the vast majority of reported studies have relied on cellulose derivatives such as cellulose nanofibrils (CNFs), carboxymethyl cellulose (CMC), or bacterial cellulose (BNC). In contrast, magnetic cellulose nanocrystals (MCNCs) remain comparatively underexplored despite offering distinct structural advantages. The highly crystalline and uniform structure of CNCs enables improved rigidity, reproducibility, colloidal stability, and biocompatibility, while abundant surface hydroxyl groups on CNCs allow versatile functionalization and more homogeneous anchoring of magnetic nanoparticles. However, these advantages have not yet been fully translated into practical systems due to several unresolved challenges, including limited control over nanoparticle distribution, insufficient understanding of interfacial bonding mechanisms, and a lack of scalable and reproducible synthesis strategies. Furthermore, the application of MCNCs in emerging technological fields remains largely unexplored. Addressing these gaps requires a shift from descriptive material development toward application-driven design, interfacial engineering, and scalable fabrication approaches, as outlined in the following sections.

### 9.1. Emerging Applications: CO_2_ Management, Acoustics, and Photonics

Beyond established environmental and biomedical uses, MCNCs are emerging as strong candidates for next-generation, sustainable technologies:

- Carbon Capture and Utilization: The robust CNC scaffold can be functionalized (e.g., with amines) to provide abundant CO_2_ adsorption sites, while embedded magnetic nanoparticles enable facile recovery and reuse [413]. For example, amino-modified CNC aerogels have achieved CO_2_ adsorption capacities of ~6 mmol g^−1^ [413]. Furthermore, CNC-templated porous composites doped with metal oxides (e.g., CeO_2_) demonstrate combined CO_2_ capture and catalytic conversion potential [414].
- Acoustic Devices: Introducing magnetic functionality broadens the use of cellulose into advanced devices like actuators and electromagnetic shields [415]. In acoustics, researchers have successfully fabricated flexible, lightweight magnetic cellulose membranes that generate sound without external magnets. The robust cellulose matrix provides the necessary structural stiffness and uniform nanoparticle distribution, offering a sustainable alternative to conventional loudspeaker components [416,417].
- Smart Photonics and Optics: The inherent optical activity and chiral self-assembly of CNCs can be coupled with magnetic responsiveness to enable the real-time tuning of light reflection, color, and polarization via external magnetic fields [418]. Integrating luminescent, plasmonic, and magnetic nanostructures within CNC frameworks could pave the way for adaptive sensors, advanced anti-counterfeiting systems, and flexible wearable optics.

### 9.2. Fundamental Interfacial Interactions

Despite the volume of research on MCNC synthesis, the fundamental bonding mechanisms between the cellulose surface and magnetic nanoparticles (whether physical adsorption, electrostatic attraction, coordination bonding, or covalent linkage) remain insufficiently characterized. Elucidating how bare or surface-modified CNCs interact with nanoparticles like Fe_3_O_4_, γ-Fe_2_O_3_, or metallic Fe is crucial for optimizing synthesis routes. Future studies must heavily integrate advanced computational methods—such as density functional theory (DFT), Bader charge analysis, and projected density of states (PDOS) calculations—to probe the electronic structure and charge-transfer behavior at the CNC–nanoparticle interface [419,420]. Combining these simulations with experimental characterization will enable the rational, predictable design of MCNCs with enhanced interfacial bonding.

### 9.3. Synthesis Optimization and Clinical Safety

Finally, translating MCNCs into practical commercial or clinical applications requires overcoming current synthesis and safety limitations. The commonly used co-precipitation method often results in uneven nanoparticle distribution and agglomeration; therefore, developing novel, highly efficient synthesis strategies is essential to produce uniform dispersions. Additionally, while cellulose and magnetic nanoparticles are generally biocompatible, the safety of nanocomposites depends heavily on the nanoparticle dosage and the applied magnetic field strength. For in vivo applications (such as drug delivery, bioimaging, and hyperthermia), future work must rigorously optimize these parameters and tailor surface modifications to ensure strict biocompatibility, prevent unwanted tissue toxicity, and maximize targeted therapeutic efficacy.

## 10. Conclusions

In conclusion, magnetic cellulose nanocrystals (MCNCs) represent a highly versatile and sustainable class of nanocomposites that seamlessly combine the tunable magnetic properties of metal oxide nanoparticles with the excellent biocompatibility and structural uniformity of cellulose. By anchoring magnetic nanoparticles onto the rigid CNC scaffold, researchers can achieve precise control over nanoparticle distribution, interfacial interactions, and magneto-responsive behavior. Consequently, MCNCs have emerged as advanced functional materials with transformative potential across a broad spectrum of fields, spanning environmental remediation, targeted biomedicine, sustainable agriculture, energy storage, and smart optical devices.

Despite significant progress in synthesis, surface modification, and functionalization strategies, several key challenges remain unresolved. In particular, achieving large-scale and reproducible fabrication while preventing nanoparticle agglomeration requires the development of controlled synthesis approaches, such as continuous-flow reactors, microemulsion-assisted methods, or hybrid synthesis strategies. Furthermore, a deeper fundamental understanding of the nanoscale bonding mechanisms at the CNC-magnetic nanoparticle interface is needed, which can be addressed through combined experimental and computational approaches.

Future research should also prioritize evaluating MCNC performance under realistic conditions, including multi-cycle stability and long-term environmental or biological exposure. For biomedical applications, systematic studies on dose-dependent toxicity, biodistribution, and clearance pathways are essential to ensure clinical safety. In parallel, application-specific optimization—such as enhancing CO_2_ adsorption selectivity, improving magnetic recovery efficiency, and integrating MCNCs into scalable device architectures—will be critical for practical implementation.

Ultimately, advancing MCNCs from laboratory-scale research to real-world technologies will require an integrated approach combining scalable synthesis, precise interfacial engineering, and application-driven design.

## Figures and Tables

**Table 1:** Sources, extraction methods, typical size, crystallinity, and yield of CNCs.

Source	Method	Size	Crystallinity (%)	Yield (%)	Ref.
Wood/pulp	Acid hydrolysis (H_2_SO_4_)	100–200 × 3–6	70–83	12–30	[22,65,66]
Cotton	Acid hydrolysis + ultrasound	100–200 × 3–6	65–85	20–30	[22]
Agricultural residues (baggase, straw, husk, banana peel, corn cob, leaves)	Acid hydrolysis (±alkali/bleach)	100–400 × 5–10	55–80	7–35	[22,67,68,69,70,71,72,73,74,75,76,77,78,79,80]
Natural fibers (flax, hemp, jute, sisal, kenaf)	Acid hydrolysis/TEMPO	150–250 × 5–8	70–85	15–28	[81,82,83,84,85,86,87]
Fruit and food waste (peels, husk, pomace)	Green/acid hydrolysis	150–300 × 8–20	65–80	15–30	[69,88,89,90,91]
Coconut/palm residues	Acid hydrolysis (±bleaching)	200–260 × 5–9	68–80	18–22	[81,92,93]
Bacterial cellulose	Enzymatic hydrolysis	100–500 × 10–50	80–90	20–40	[94]
Tunicates/algae	TEMPO/acid hydrolysis	200–1000 × 10–20	>90	10–20	[95,96]
Other biomass (bamboo, switchgrass, shells)	DES/acid/mechanical	20–300 (width)	60–80	10–90	[97,98,99,100,101,102]

**Table 2:** Synthesis method of magnetic NPs along with their strengths and limitations.

Synthesis Method	Strengths	Limitations	Ref.
Ball Milling Method	High efficiency, uniformity in particle size, ability to produce very fine powders, easy process, high yield.	Contamination issues, large size distributions, long processing times.	[155,156]
Laser Evaporation	High production efficiency, low cost, good stability, reliable processing quality	Contamination of product, wide size distribution	[157]
Wire Explosion Method	High productivity clean and safe process, produces spherical NPs with narrow size distribution.	Non-monodispersed particle sizes, presence of aggregates requiring additional processing.	[125]
Coprecipitation	Simple, large quantity	Impurities, time consuming	[158]
Thermal Decomposition	High crystallinity, controlled size, well-defined shape, ability to produce monodispersed NPs.	Product contamination and challenges in achieving smaller particle sizes.	[159]
Microemulsion Synthesis	Good size distribution, crystal shape control, low defect levels, and the ability to synthesize large, high-quality crystals.	High costs and potential contamination of the product due to residual surfactants.	[160]
Hydrothermal/Solvothermal	Ease of synthesis, good control over particle size and morphology, high-quality NPs	Use of toxic reactants, high energy costs, challenges in controlling shape	[161,162]
Sol–gel Method	High purity, homogeneous composition, cost-effective, allows for control of size and shape.	Production of toxic organic solvents, difficulty in controlling morphology.	[145]
Sonochemical Reaction	High yields, cost-effective, reduced environmental impact, fast reaction times.	Potential contamination, requires specific conditions to avoid aggregation.	[163]
Microwave	Simple, time-saving, low energy-consuming, produces monodisperse NPs with good magnetic properties	Microwave reactor required	[164]
Chemical Reduction	Simple, cost-effective, environmentally friendly, produces monodisperse NPs	Challenges in controlling particle size and potential agglomeration	[165,166]
Chemical Vapor Deposition	High purity, uniformity, cost-effective, ability to produce fine coatings.	Potential contamination of the product, wide size distribution of NPs.	[167,168]
Arc Discharge	Simple, low cost, high productive capacity, produces NPs that crystallize by themselves	Difficult to control particle size	[169]
Laser Pyrolysis	Highly localized heating and rapid cooling	Expensive, scalability	[170]
Combustion Synthesis	Fast route to produce nanostructures with high surface area; ability to synthesize various magnetic phases by optimizing combustion parameters.	Contamination of the product; wide size distribution of NPs.	[171,172]
Biological Method	Efficient, clean process, ecofriendly	Poor dispersion of NPs	[173]

**Table 3:** Comparative evaluation of MCNC synthesis methods, including advantages, limitations, and mitigation strategies.

Method	Size Control	Scalability	Cost	Limitations	Potential Solutions	Ref.
Co-precipitation	Low to moderate	High	Low	Broad size distribution; aggregation; limited crystallinity control	Control pH, temperature, and CNC surface modification (e.g., TEMPO oxidation)	[177,178,179,206]
Thermal decomposition	High	Low	High	Higher costs; complex processing	Post-synthesis surface modification; ligand exchange strategies	[182,183,184,199,207]
Microemulsion	High	Low	High	Surfactant residues; difficult purification; low scalability	Surfactant removal (dialysis/solvent exchange); green microemulsion systems	[188,189]
Hydrothermal	Moderate to high	Moderate	Moderate	High pressure/temperature; batch variability	Microwave-assisted hydrothermal optimization for reproducibility	[181,185,187]
Ultrasonic irradiation	Moderate to high	Moderate to high	Moderate	Reduced crystallinity, weak magnetic coupling	Optimize power/time; combine with mild hydrothermal or co-precipitation	[178,192,193,194]
Microwave-assisted synthesis	High	Moderate	Moderate	Hot-spot formation; scale-up challenges	Controlled microwave reactors; stepwise heating; continuous flow systems	[197,198,199]

## Data Availability

No new data were created or analyzed in this study. Data sharing is not applicable to this article.

## Supplementary Materials

The following supporting information can be downloaded at: https://www.mdpi.com/article/10.3390/nano16110645/s1, Table S1: Sources and Extraction Methods of Cellulose Nanocrystals (CNCs); Section S1: Synthesis Routes for Magnetic Nanoparticles; Table S2: Summary of recent studies on magnetic cellulose-based nanocomposites fabricated through various synthesis routes.
