# Supplementary material for: Magnetic Cellulose Nanocrystal Composites: Synthesis, Properties, Applications, and Opportunities
Source: Nanomaterials (Basel). 2026 May 22;16(11):645. doi: 10.3390/nano16110645 (PMC13258494; doi:10.3390/nano16110645)
Supplement: Supplementary file 1 [file nanomaterials-16-00645-s001.zip › nanomaterials-4270058-supplementary.pdf]

Supporting Information

Magnetic Cellulose Nanocrystal Composites: Synthesis, Properties, Applications and Opportunities

Mohammad Jahid Hasan,[1†] Kishore Chand,[2†] Esteban E. Urena-Benavides,[1] and Erick S. Vasquez-Guardado[2,3]*

[1] Department of Biomedical Engineering and Chemical Engineering, The University of Texas at San Antonio, San Antonio, TX 78249, USA; mohammadjahid.hasan@utsa.edu (M.J.H.); esteban.urena-benavides@utsa.edu (E.E.U.-B.)
[2] Department of Chemical and Materials Engineering, University of Dayton, 300 College Park Ave., Dayton, OH 45469, USA; chandk1@udayton.edu
[3] Hanley Sustainability Institute, University of Dayton, 300 College Park, Dayton, OH 45469, USA
* Correspondence: evasquez1@udayton.edu
† These authors contributed equally to this work.

Table of Contents:

Table S1……………………………………………………………………………………... (3)
Section S1………………………………………………………………………………… (7)
Table S2……………………………………………………………………………………..(13)

Table S1. Sources and Extraction Methods of Cellulose Nanocrystals (CNCs)

Source	Extraction / Pretreatment Method	Typical Dimensions (L*W-nm)	Crystallinity (%)	Yield (%)	Reference
Wood (Hardwood / Softwood)	Sulfuric acid hydrolysis	100–200 × 3–5 nm	70–85	12–30	[1]
Cotton	Sulfuric acid hydrolysis (60–70 %, 45 °C)+ ultrasound	100–400 × 3–6	65–85	20–30	[1]
Sugarcane bagasse	Green acid hydrolysis (alkali pretreatment + mild H_2SO_4 / formic acid route)	200–400 × 5–10	72	32	[1]
Wheat straw	Acid hydrolysis (60 %, 45 °C, 45 min)	110–180 × 7–15	57	~20	[1]
Rice straw	H_2SO_4 hydrolysis (after steam explosion + bleaching)	100-300 × 5–10	60–70	7–12	[2]
Corn stover	Oxalic acid hydrolysis (after alkali pretreatment + ultrasonication)	300 × 5–10	70–80	18–25	[3]
Palm fiber waste	Bleaching (acidified $NaClO_2$ + alkali) + H_2SO_4 acid hydrolysis (62 wt %)	200 × 6	68–80	15–22	[4]
Hemp fiber	TEMPO oxidation + acid(2,2,6,6-tetramethylpiperidine-1-oxyl) + H_2SO_4 acid hydrolysis (0.9 M, 50 °C, 15 min)	250 × 7	78–86	20–25	[5]
Jute fiber	Sulfuric acid hydrolysis(after alkali delignification + bleaching pretreatment)	200 × 7	79–83	15–20	[6]
Sisal fiber	Phosphoric acid hydrolysis(85 %, 60 °C, 5 h + water regeneration)	150 × 5 – 8	70–75	18–30	[7]
Date pits	H_2SO_4 hydrolysis(after NaOH delignification + $NaClO_2$ bleaching)	150 × 4	68–72	20–28	[4]
Banana peel	Alkali + bleaching pretreatment followed by mild acid ("enzyme-assisted-like") hydrolysis	200 × 10	65–75	22–29	[8]
Orange peel	Citric acid hydrolysis(80 °C, 150 min) + ethanol/bleach pretreatment	250 × 8	67–75	25	[9]
Coffee husk	Alkali pretreatment + bleaching + H_2SO_4 acid hydrolysis (64 wt %, 45 °C, 45 min)	280 × 10	70	17	[10]
Bacterial cellulose	Enzymatic hydrolysis using cellulase (Trichoderma reesei) after mechanical pretreatment	100–500 × 10–50	80–90	20–40	[11]
Tunicates	TEMPO oxidation (2,2,6,6-tetramethylpiperidine-1-oxyl) + H_2SO_4 acid hydrolysis (64 wt %, 2 h)	200–1000 × 10–20	> 90	10–15	[12]
Algae (Cladophora sp.)	Acid hydrolysis H_2SO_4 acid hydrolysis (60 wt %) with microwave-assisted H_2O_2 bleaching	250 × 12	65–85	12–20	[13]
Trichoderma reesei fungal cellulose	Ezymatic hydrolysis using T. reesei cellulase complex (50 °C, pH 5.0, 12–48 h)	150 × 8	70–80	10–18	[14]

Source	Extraction / Pretreatment Method	Typical Dimensions (L*W-nm)	Crystallinity (%)	Yield (%)	Reference
Softwood pulp	Southern bleached softwood kraft pulp + H_2SO_4 acid hydrolysis (64 wt %, 45 °C, 50 min)	120 × 6	-	25	[15]
Hardwood pulp	Sulfuric acid hydrolysis (60–65 wt %, 45–60 °C, 30–60 min)	200 × 5	72 – 83	20–30	[16]
Bagasse ash fiber	Alkali delignification + bleaching + HCl hydrolysis (2.5 M, 90 °C, 1 h)	180 × 6	70	18	[17]
Oat straw	Oxalic acid hydrolysis (0.3 M, 90 °C, 120 min) after Na OH delignification & bleaching	290 × 5	73	20	[18]
Tea waste	Green acid acid hydrolysis (formic + citric acid, 1:1 v/v, 85 °C, 2 h) after alkali delignification & bleaching	180 × 9	68	14	[19]
Pineapple leaf	H_2SO_4 hydrolysis (55 wt %, 45 °C, 30 min) after NaOH + $NaClO_2$ pretreatment	250 × 6	66	16	[20]
Sugarcane leaves	Enzymatic (T. reesei cellulase, 50 °C, 12 h) + mild HCl hydrolysis (1 M, 80 °C, 1 h) after alkali + bleaching pret reatment	300 × 10	72	22	[21]
Coconut Coir fiber	Green acid (formic + citric, 1:1 v/v, 85 °C, 2 h) after Na OH delignification + $NaClO_2$ bleaching	260 × 9	68	21	[22]
Aloe vera fiber	Citric acid hydrolysis (0.5 M citric acid, 90 °C, 90 min) after ethanol extraction, NaOH (4 %) alkaline treatment and H_2O_2 bleaching	270 × 9	65	15	[23]
Cocoa pod husk	Oxalic acid hydrolysis (5 wt %, 125 °C, 30 min) after So xhlet extraction + NaOH + $NaClO_2$ pretreatment	250 × 6	80	18	[24]
Corn cob	Sulfuric acid hydrolysis (60 wt %, 45 °C, 60 min) after NaOH delignification + bleaching ($NaClO_2$)	240 × 10	75	23	[25]
Oil palm mesocarp	Alkali (4 wt % NaOH, 80 °C, 3 h × 3) + $NaClO_2$ bleaching (80 °C, 4 h × 3) →H_2SO_4 hydrolysis (65 wt %, 45 °C, 45 min)	230 × 4.5	77	24	[26]
Paddy straw	Acid hydrolysis (H_2SO_4/HNO_3, 150–220 °C, 1–5 %) followed by enzymatic saccharification for nanoc ellulose production	180–270 × 4–10	56-76	25-45	[27,28]
Corn cob	Delignification: 10 % NaOH, 100 °C, 1 h → Bleaching: 30 % H_2O_2 (alkaline pH 8–12, 90 °C, 1 h) → Purification: 17.5 % NaOH, 80 °C, 30 min (α-cellulose production) → Hydrolysis: 30 % H_2SO_4, 50 °C, 30 min + mechanical defibrillation using 11,000 rpm blender (15 min) under mild conditions → washing / centrifugat ion → ultrasonication (80 % amplitude, 50 °C, 30 min)	Length > 100 n m, Diameter 6.5–9.4 nm (rod-shap ed CNC, high as pect ratio ≈ 15 – 20)	Cellulose N CC: 69.9 α-cellulose NC C: 65.5	Cellulose basis: 50.1 α-cellulose basis: 65 .3	[29]
Coffee husk	Alkali treatment (4 wt % NaOH, reflux 3 h × 2) → Blea ching (1.7 wt % $NaClO_2$ in acetate buffer, reflux 4 h × 6 cycles) → Acid hydrolysis (64 wt % H_2SO_4, 50 °C, 40 min, 7.5 wt % solid) → neutralization + dialysis + sonic ation (30 min, 750 W)	310 ± 160 × 20 ± 4 nm	≈ 80 ± 2	≈ 41	[30]

Source	Extraction / Pretreatment Method	Typical Dimensions (L*W-nm)	Crystallinity (%)	Yield (%)	Reference
Tomato pomace	Alkali + bleaching + acid hydrolysis + ultrasonication<br><br>Or<br>Alkaline treatment: 2 wt % NaOH (95 °C, 4 h × 4 cycles) to remove lignin and hemicellulose → Bleaching: 1.7 wt % $NaClO_2$ with acetate buffer (80 °C, 6 h × 2) → Acid hydrolysis: 64 wt % H_2SO_4 (45 °C, 30 min) followed by dialysis → ultrasonication (5 min, 750 W) for CNC dispersion	≈ 110 × 104 ± 10	79 (best condition at 45 °C for 30 min)	≈ 19.8	[31]
Barley straw	Alkali pretreatment + enzymatic step + acid hydrolysis<br><br>(1) Chemical route: $NaClO_2$ (0.7 wt %, pH 4) 2 h + 17.5 % NaOH 20 min at RT. (2) Enzymatic route: 5 % NaOH wash + autoclaving (120 °C, 1.5 atm) + pectinase and cellulase (24 h each at 45 °C). → Acid hydrolysis (64 wt % H_2SO_4, 45 °C, 30 min) + dialysis + ultrasonication (5 min)	CNCstraw chem: Irregular/spherical; low reaction ≈ 5 % yield. CNC straw enz: Needle-like (270 ± 40 × 15 ± 5 nm)	≈ 77 – 79 (CNC from enzymatic route higher	≈ 5 (Chem); ≈ 5 (Enz)	[32]
Purple sweet potato peel	Ultrasound-assisted maleic acid hydrolysis<br>or<br>After anthocyanin extraction → amylase treatment (95 °C, 30 min + 5 % enzyme 3 h) → NaOH (10 %, 60 °C, 4 h) alkaline delignification → NaClO bleaching (70 °C, 2 h × 4 cycles) → Ultrasound-assisted maleic acid hydrolysis (75 wt %, 475 W, 60 °C for 1 h + 120 °C for 2.5 h) → dialysis and freeze-drying	60–220 × 10–30 (rod-like CNCs, aspect ratio 25 ± 0.2)	58.3	8.17	[33]
Hazelnut shell	Acid / alkali treatment + ball-milling + ultrasonication<br>Or<br>Alkaline treatment: 3 wt % NaOH (80 °C, 3 h × 2) → Bleaching: 2.7 % $NaClO_2$ in acetate buffer (pH 4.9, 80 °C, 1 h × 3) → Acid hydrolysis: HCl (3 M, 90 °C, 180 min, 1:40 w/v) → dialysis (pH 7) → ultrasonication (300 W, 20 min) → freeze-drying	≈ 157 ± 10 nm (diameter) (spherical CNCs)	69.6 ± 0.1	67.3 ± 2.3	[34]
Jackfruit peel	Alkali + bleaching + acid hydrolysis + rotary evaporation<br>Or<br>Dewaxing (water + ethanol Soxhlet 8 h) → Bleaching (1.5 % $NaClO_2$, pH 3.5, 70 °C, 2 h × 4) → NaOH (alkali treatment, 5 %) → H_2SO_4 hydrolysis (65 %, 37 °C, 1 h, 1:9 g/mL) → neutralization + dialysis (pH 5.5) → ultrasonication (15 min, bath type)	Spherical CNCs ≈ 130 nm (avg.), non-agglomerated soft SCNCs	83.4	7 (SCNC fraction) ($C_6H_{10}O_5$ base)	[35]
Moso bamboo (Phyllostachys pubescens)	Deep eutectic solvent (DES) + microfluidization<br><br>Or<br>Pretreatment: Choline chloride + lactic acid DES (1:9 mol ratio), 120 °C, 3 h, solid:liquid = 1:25 → delignification (94.4 %) → mechanical disintegration (microfluidizer) to CNC / CNF (1 wt %)	Width: 20–80 nm (fibril bundles); length: > 4 µm (CNF aggregate, AFM traced)	60.4 (± 0.8) (raw bamboo = 49.2 %)	≈ 91 % cellulose recovery (post-treatment)	[36]
Switchgrass (Panicum virgatum L.)	Alkali + bleaching + acid hydrolysis + ultrasonication<br>OR<br>Hydrothermal pretreatment (autoclaving 180–200 °C for 30–60 min) → Mechanical fibrillation (supermass collider, 1 wt% % suspension) → dry-casting of lignin-containing cellulose nanofibrils (LCNF). Chitosan (CS) solution (1.5 wt% % in acetic acid) was	6.5 – 47.6 nm (width of individual LCNFs, filamentous structure)	Amorphous to semi-crystalline (cellulose I)	Lignin removal: up to 40 % (recovery of cellulose ≈ 85 %)	[37]

Source	Extraction / Pretreatment Method	Typical Dimensions (L*W-nm)	Crystallinity (%)	Yield (%)	Reference
	used to prepare CS/LCNF films via solution casting and drying at 40 °C for 48 h.				
Tea stalk	Alkali + acid hydrolysis + ultrasonication<br>OR<br>Dewaxing (H_2O_2 + acetic acid 1:1 mole ratio, 60 °C, 24 h) → acid hydrolysis (H_2SO_4 62.2 %, 45 °C, 123 min, 1:15 w/v) → dialysis (pH 6.5-7.0) → ultrasonication (20 min, 1000 W) → freeze-drying	100–300 nm *4–8 nm (rod-like TNCCs)	61.3 (TNCC) (TPCF: 59.5; raw tea stalk: 36.1)	49.9 (optimized RSM yield)	[38]
Pepper plant stem residue (Capsicum annuum L.)	Direct acid hydrolysis (H_2SO_4)<br>Or<br>Alkaline treatment: 4 wt % NaOH (80 °C, 2 h × 3 cycles) → Bleaching: 1.7 wt % $NaClO_2$ in acetate buffer (pH 4.9, 80 °C, 2 h × 3) → Acid hydrolysis: for CNCPs (H_3PO_4 9 M, 100 °C, 2 h) and for CNCSs (H_2SO_4 64 wt %, 50 °C, 30 min) → dialysis → ultrasonication (5 min, 250 W)	CNCPs: 43 ± 15.1 × 20–50 nm CNCSs: 31 ± 8.8 × 15–40 nm (needle-like rods)	CNCPs: 84.78 CNCSs: 85.41	Not quantified (cellulose recovery ≈ 80 % post-bleaching; acid CNC yield ≈ 10–12 %)	[39]
Pine trunk	Acetosolv pulping + bleaching + acid hydrolysis + spray-drying<br><br>Or<br>Acetosolv pulping: acetic acid + 0.3 % HCl (115 °C, 3 h) → Bleaching: 4 % NaOH + 24 % H_2O_2 (50 °C, 150 min) → Hydrolysis: H_2SO_4 62 wt % (44 °C, 90 min, 1:10 w/w) → centrifugation and dialysis (pH 7) → spray-drying	100 – 300 *220 ± 20	67.8 ± 2.1	≈ 9.0 (of microcrystalline cellulose reference)	[40]

Section S1: Synthesis Routes for Magnetic Nanoparticles

Physical method

Physical synthesis methods for MNPs offer unique benefits in terms of purity, scalability, and control over specific particle characteristics. In the physical synthesis of MNPs, the production of NPs primarily relies on mechanical and physical processes. The main physical approaches to MNPs synthesis include top-down and bottom-up techniques. Top-down methods involve breaking down bulk material into nanoscale particles, while bottom-up strategies involve assembling NPs from atomic or molecular units. Each approach has its own pros and cons, and the selection depends on the application.

Ball Milling

Ball milling is a top-down process where bulk materials are mechanically ground into nanoparticles. It is an affordable method that does not require expensive chemicals and produces no hazardous waste. Recent reports indicate that optimizing parameters such as the ball-to-powder ratio, milling time, and speed can enhance the quality of produced MNPs. For example, nanocrystalline magnesium ferrite was synthesized by Bououdina et al. in 2019 via ball milling, yielding particles with the lowest coercivity and remanence, making them suitable for heavy metal removal from wastewater.[41] Processes involved include being simple, cost-effective, environmentally friendly, and suitable for large-scale production, while drawbacks include difficulties in achieving adequate control over particle shape and size.

Laser Ablation

Laser ablation is a bottom-up approach that utilizes a high-power laser to vaporize target materials immersed in a liquid medium, thereby generating NPs. The technique has the advantage that the NPs it produces have high purity and peculiar structures [42]. Recent advances in laser technology have allowed more control over particle size distribution and morphology. For instance, Alonso et al. (2018) reported the synthesis of iron oxide NPs with enhanced magnetic properties via laser ablation [43]. The advantages of these methods are as high purity of produced NPs, the ability to create unique nanostructures, and good control over particle size whereas drawbacks are relatively low production rates, high equipment costs, and energy-intensive processes.

Wire Explosion Method

The wire explosion method involves rapid vaporization of metal wires in a high-current pulse, followed by condensation into NPs in a single step, of offering minimal energy use and eco-friendliness. Song et al. (2013) demonstrated the effectiveness of this technique to produce iron oxide MNPs for arsenic removal from water.[44] Since this method is environmentally friendly, it allows for requirement, minimal energy use, and high productivity. The drawbacks include difficulty in achieving monodispersed particles, limited control over particle morphology, and the potential for particle agglomeration.

Electron Beam Lithography

Electron beam lithography is a highly effective method for patterning nanostructures on substrates. It provides a high degree of control over size, shape, and arrangement that is unattainable with other synthesis methods for magnetic NPs [45]. Recent work has demonstrated the effectiveness of this method in creating patterned arrays of MNPs with homogeneous sizes and shapes. For instance, Wang et al. (2019) used electron beam lithography to fabricate iron oxide NPs with controlled dimensions for magnetic hyperthermia applications [45]. This approach offers several advantages: exceptional control over particle size and shape, the ability to fabricate complex nanostructures, and high reproducibility. Limitations include low throughput, high equipment and operational costs, and substrate-based synthesis.

Gas-Phase Deposition

Physical and chemical methods are chosen depending on the specific deposition technique and the underlying mechanisms. For example, in techniques like CVD and PVD, gas-phase deposition is carried out by forming NPs from gaseous precursors. Such approaches are especially valued for their ability to achieve highly accurate control over particle composition, thereby enabling the production of very pure NPs [46]. Grammatik Poulos et al. (2016) pointed out that gas-phase deposition enables the fabrication of complex nanostructured bimetallic systems with designed magnetic properties.[47] It has several advantages, including high purity, compositional control, and the ability to form intricate nanostructures. At the same time, several disadvantages are associated with this process, including the need for high temperature/vacuum conditions, relatively low production rates, and high energy demands.

Inert Gas Condensation

Inert gas condensation involves evaporating the source metal in an inert gas environment, then rapidly cooling to facilitate NP formation. This technique has produced ultrafine magnetic NPs with narrow size distributions. [48] More recently, it has demonstrated its potential to develop magnetic NPs with enhanced magnetic properties. For instance, Hammad et al. (2020) synthesized iron oxide NPs by inert gas condensation; the resulting magnetic NPs exhibited enhanced magnetic hyperthermia performance for heat generation [48]. The advantages of this technique include the ability to produce ultra-fine NPs with precise size control and well-defined particle composition. However, this method has several drawbacks, including the need for special equipment, an inert atmosphere, and relatively low production rates, as well as the tendency of particles to agglomerate.

Chemical method

Chemical synthesis techniques for magnetic NPs primarily employ a bottom-up approach, enabling the assembly of NPs from their atomic or molecular precursors. The following is a summary of some of the common methods widely recognized to be effective in the synthesis of MNPs. In chemical synthesis, Fe_3O_4 magnetic nanoparticles are produced through controlled bottom-up assembly from ionic precursors. Among these, co-precipitation and hydrothermal/solvothermal processes are the most used because of their simplicity, scalability, and ability to control crystal phase and size. The co-precipitation method, based on alkaline reduction of Fe^{2+} and Fe^{3+} salts at pH 8–14 (Fe^{3+} / Fe^{2+} = 2:1), allows for rapid formation of uniform Fe_3O_4 nanoparticles within

minutes under mild conditions. Meanwhile, hydrothermal synthesis provides better control over morphology and crystallinity at elevated temperature and pressure [49,50].

Spray Pyrolysis

Among the various techniques for preparing MNPs with improved properties, spray pyrolysis is among the most effective methods. This technique involves atomizing the precursor solution into small droplets, which are then conveyed by a gas through heated reactors where the solvent evaporates and particles form. The main advantages of this technique include continuous production, control of particle size distribution, and the ability to synthesize MNPs with enhanced magnetization and photocatalytic properties [8, 27]. Kaya et al. [51] demonstrate that MNPs prepared by spray pyrolysis exhibit superior magnetization and extraordinary photocatalytic degradation performance.

Laser Pyrolysis

Laser pyrolysis is regarded as one of the new synthesis techniques of MNPs. This method is based on heating a gaseous precursor mixture using a CO_2 laser, thereby inducing and sustaining chemical reactions. Its principal advantages over other techniques include extremely high heating and cooling rates, allowing the preparation of small, uniform particles with precisely adjusted properties. Recent studies have demonstrated its effectiveness in synthesizing iron oxide NPs with improved magnetic properties. For instance, this technique has achieved magnetic saturation as high as 70 emu/g [52].

Co-precipitation

Co-precipitation is one of the most widely used methods for synthesizing superparamagnetic iron oxide nanoparticles (SPIONs) with diameters below 50 nm. It involves a chemical reaction in aqueous solution, forming iron hydroxide nuclei that grow into NPs [53–55]. Recent work has shown that co-precipitation is one method that enables the synthesis of stable, recyclable magnetic nanoparticles. For instance, Darwesh et al. co-precipitated Fe_3O_4 MNPs with glutaraldehyde and showed that the particles remained stable for 90 days and retained high activity after 100 cycles of reuse [56]. Besides, Marinin et al. (2023) further optimized the co-precipitation process by varying pH, temperature, and iron salt concentration, thereby enabling better control over particle size and morphology [57].

Thermal Decomposition

Among efficient techniques for synthesizing MNPs, thermal decomposition enables control not only of their size but also of their shape. The decomposition of metal precursors, such as oleates and acetylacetonates, in the temperature range between 150 °C and 300 °C, along with high-boiling organic solvents, has been significantly improved over the last few decades. For instance, Effenberger et al. demonstrated that using molecular oxygen during synthesis resulted in MNPs with extremely thin magnetically dead layers, thereby significantly enhancing their magnetic properties. Recently, Martinez de la Torre and Bennewitz demonstrated the synthesis of manganese oxide NPs via one-pot thermal decomposition but further exemplified the synthesizing of a wide array of metal oxides through this technique [42,58,59].

Microemulsion

The synthesis of MNPs has become versatile using microemulsions, which employ a two-phase system of water and oil, along with a surfactant. This approach offers precise control over nanoparticle size, shape, and surface area, making it highly valuable for the synthesis of various MNPs with customized properties. Marinin et al. (2023) used a microemulsion to synthesize nickel NPs with optimized size and shape, demonstrating the method's wide applicability for fabricating a variety of MNPs [60–62].

Hydrothermal/Solvothermal Method

Hydrothermal treatment has been one of the versatile techniques for MNPs synthesis, and it can be carried out under high pressure above 13790 kPa and at temperatures above 200°C. In this method, excellent control over particle size, shape, and composition may be achieved. Recently, different types of MNPs have been prepared using this technique with their properties tailored towards the desired applications. For example, Abd et al. prepared hematite (α-Fe_2O_3) NPs by hydrothermal methods and observed that, with increasing reaction time, the crystallite size also increased [63]. Similarly, Mora-Cabello et al. demonstrated control over the morphology and colloidal stability of MNPs synthesized via hydrothermal processes [64].

Sol-Gel Method

Sol-gel is generally recognized as one of the most versatile approaches for preparing MNPs and metal oxide NPs. The advantages are low-temperature processing, possibility of control of reaction kinetics, and flexibility in compositions that can be prepared. Generally, the method utilizes hydrolysis and condensation of molecular precursors in an aqueous solution to form a sol which eventually transforms into a gel [65]. For example, Shabelskaya et al. (2023) synthesized Co(II) ferrite NPs using a simple sol-gel technique, resulting in increased surface area and good magnetic properties for a wide range of applications.[66] Hu et al. (2024) have also utilised an eco-friendly coconut water-assisted sol-gel method to synthesize magnetite NPs, further extending the approach's versatility and environmental benefits.[67]

Polyol Method

The polyol technique is the most versatile approach for MNP synthesis, as it provides controlled size and shape. Indeed, this synthesis employs polyols such as propylene glycol or polyethylene glycol, which limit particle growth while maintaining high crystallinity [68]. Recent work has extended the application of the polyol technique for the preparation of bimetallic clusters and nanocrystalline alloys, with potential applications in wastewater treatment. In 2021, Wang et al. prepared iron carbide NPs using a modified polyol method, thereby expanding their usability in complex biomedical applications [69]. Although the polyol method offers excellent control over particle characteristics, it is sensitive to the type of metal being reduced. [70–72].

Non-Thermal Plasma Methods

Non-thermal plasma methods have emerged as a promising, eco-friendly alternative technique for synthesizing MNPs. Offering low gas temperature and high electron energy compared to conventional high-

temperature plasma methods, they are considered more eco-friendly. Recently, the versatility of non-thermal plasma in producing various nanomaterials, including metal NPs and metal oxides, has been demonstrated. In 2024, Ma et al. reported the successful synthesis of highly purified titanium nanocrystals via a capacitively coupled plasma route, demonstrating the potential of this technique for developing new nanostructures. While non-thermal plasma methods are very promising, scalability and cost-effectiveness will need to be further explored for large-scale MNP production [73].

Biological synthesis method

The biological synthesis of metal nanomaterials and MNPs has been recognized as a feasible, environmentally friendly alternative to physical and chemical methods of nanoparticle synthesis. It is based on the use of living organisms plants, microorganisms, their extracts, fungi, viruses, bacteria, and actinomycetes —with potential for preparing NPs with specific properties and applications. This methodology offers several advantages, including low cost, biocompatibility, and scalability. It also suffers from drawbacks such as poor NP dispersion and an inability to fully understand the underlying mechanism [74,75].

Biological sources for the synthesis of metal nanomaterials and MNPs are quite varied. Microorganisms, such as bacteria, fungi, and actinomycetes, have been shown to produce NPs both intracellularly and extracellularly. Plant-based synthesis using plant leaves, stems, roots, and seeds, and their extracts/exudates, has attracted considerable interest due to its simplicity and eco-friendliness. Although the mechanism of NPs formation is yet to be fully realized, some suggested pathways include the activity of nitrate reductase, shuttle electron transport via quinones, and mixed mechanisms [42,42,76].

MNPs biologically synthesized have applications in several fields, including biomedical applications, catalysis, and photocatalysis. The most challenging areas of biological synthesis include improving yield, enhancing dispersion, elucidating the exact mechanisms of formation, and optimizing size control and morphological characteristics.[75,77].

Table S2. Summary of recent studies on magnetic cellulose-based nanocomposites fabricated through various synthesis routes, highlighting their compositional features, characterization outcomes, magnetization behavior, and multifunctional applications in adsorption, catalysis, and biocatalytic systems.

**Composite Type**	**Synthesis Method**	**Main Results**	**Limitations**	**Application**	**Magnetization (emu g^{-1})**	**Ref**
MZeo/Cellulose nanofiber	Co-precipitation method, ultrasonication, and vacuum-drying	Fe_3O_4 NPs well-dispersed; superparamagnetic; 99% RR198 dye removal (pH 2, 45 min); kinetics = PSO	Performance after five cycles, weaker magnetism, acidic pH needed	Dye adsorption (real/synthetic waters)	48 (bare Fe_3O_4; lower in composite)	[78]
Fe_3O_4 @SA–CMC–CuNP nanocomposite	Co-precipitation and sonication-assisted in situ loading.	CuNPs (11.3 wt%, ~10 nm) well-dispersed; BET 168 m^2/g (vs. 142); 95 % 4-NP→4-AP in 1.5 min; k=0.359/min; Ea=18.5 kJ/mol; >90 % reusable 5 cycles	Slight Cu leach, small activity loss after cycles	4-NP reduction, wastewater catalysis	43.5 (Fe_3O_4=55.1; Fe_3O_4@SA–CMC=40)	[79]
Fe_3O_4@Cellulose (Pennisetum straw)	Acid hydrolysis and co-precipitation.	81 % cellulose; Fe_3O_4 (10–20 nm) uniform; surface area 1.9→56.5 m²/g; TC q_{max}=44.9 mg/g (pH 7); superparamagnetic; high stability	Reuse drops after 3 cycles; affected by humic acid/ions; non-selective for antibiotics	Tetracycline removal, green adsorbent from waste straw	32.8 (1.2$_{(rem)}$, 22.7$_{(coerc)}$)	[80]
Pineapple peel cellulose/magnetic diatomite (PPC/m-DE) hydrogel	Co-precipitation and ionic-liquid-assisted regeneration with freeze-drying.	m-DE (Fe_3O_4@diatomite) well-dispersed; BET, swelling, & thermal stability ↑; q_{max} = 101.9 mg/g (MB, 30 min, 30 °C); PSO, Langmuir fit; >90% reuse (4x); superparamagnetic	Capacity with excess m-DE or crosslinking; low selectivity for other dyes	Fast MB dye removal, magnetically recoverable	54.7 (m-Fe_3O_4); 5.8 (m-DE); 2.5 (m-DE/PPCH-3)	[81]
Magnetic cellulose powders from Juncus effusus- M-JEPs (Juncus effusus cellulose)	Chemical co-precipitation and calcination.	Fe_3O_4 NPs (10–20 nm) uniform, 64% porosity, 61 m^2/g area; max adsorption q_{max} = 76 (RR195), 99 (RB222) mg/g; pH ~6.5, Langmuir/PSO fits; 90% efficiency, eight cycles.	Fe_3O_4 surface agglomeration; recovery in salt, moderate Ms.	Magnetic dye removal, fast/eco-friendly textile effluent	(composite); baseline Fe_3O_4 = 74	[82]
Modified porous magnetic cellulose/Fe_3O_4 beads (MCFBs)	Sol-gel transition in ionic liquid and grafting.	Well-dispersed Fe_3O_4 (16.7 wt %), 8.2 m²/g surface, MB q_{max}=1187, RhB q_{max}=152 mg/g, PSO/Langmuir fit, 88-82% after five cycles, superparamagnetic.	area after grafting, efficiency in salt, Ms moderate	Fast cationic dye removal with a green, recyclable bio adsorbent.	Fe_3O_4: 88.9, MCFBs: 9.3 (<10 s separation)	[83]
Nanocrystalline jute cellulose/ Fe_3O_4/Ag (NCJC/ Fe_3O_4 /Ag) nanocomposite	Acid hydrolysis, in-situ co-precipitation, and bio-reduction.	75 nm cellulose, 16 nm Fe_3O_4, 24 nm Ag NPs; Fe_3O_4~25%, Ag~28%; stable to 700 °C; 100% Congo Red degradation (8–13 min, $NaBH_4$); antibacterial (zones: 21/17 mm), antioxidant (88%); 80% reuse (3 cycles)	Partial magnetism loss after three cycles; Ag^+ leaching (minimal), moderate Ms.	Catalysis (azo dye), antibacterial, antioxidant, packaging, wound, environmental	8.9→6.7 (paramagnetic, easy recovery)	[84]
Magnetic nanocellulose aerobeads (MABs) from recycled boxboard cellulose fibers – Fe_3O_4 /CNF composite	Mech. nano fibrillation, coprecipitation, silylation, freeze-drying	Porosity 98–99.7 %; density 0.005–0.028 g/cm^3; uniform Fe_3O_4 (~10 nm) + hydrophobic silyl shell; contact angle >148°; absorption up to 279 g/g (oil), selectivity 78–98 %, >66 % reuse (10x); superparamagnetic, <10 s recovery	Needs freeze-drying; reduced oil selects. after Fe_3O_4; low bulk strength at low CNF	Oil/solvent removal; marine/chemical spill sorbent	12.3 (MAB 0.7%)	[85]
Magnetic hydrophobic cellulose aerogels (from refined hardwood pulp fibers)	Silanization and magnetic aerogel fabrication.	Porosity 98.8%, density 0.0167 g/cm^3; WCA 146°, superhydrophobic; oil adsorption (motor 176, silicone 181 g/g); high selectivity (oil/water ~10^3); >50% reuse after 10 cycles; superparamagnetic	Fe_3O_4 slightly reduces oil capacity, freeze-dry step, limited ultrafine vapor uptake	Marine/solvent spill removal; superhydrophobic, magnetically recoverable sorbent	11.2 (superparamagnetic)	[86]
Cellulose-coated magnetic Janus nanoparticles (MJ NPs) with asymmetric surface wettability	Two-step Pickering emulsion and polymer coating.	~120 nm, hydro/hydrophilic ~1.07; int. tension 17.0→10.3 mN/m; dewatering eff. 95% (1 wt% dose, 250 mT), <1 wt% water left, >90% reuse (6x), fast magnetic sep.	~5% decline after six cycles, moderate Fe_3O_4 = slower sep. in viscous mediums	Crude oil/bitumen dewatering; magnetically collectable demulsifier	9.8 (superparamagnetic)	[87]
5-Fluorouracil-loaded magnetic cellulose bionanocomposites (MC/5-F	Isolation of Cellulose from Rice Straw, In-Situ Co-	Fe_3O_4 NP (~11 nm) on cellulose (87 nm); loading 12 %, efficiency 62.5 %; pH/heat-responsive release (45 % at pH 1.2, 86–	Slightly lower efficacy in chip models, limited	Controlled drug delivery and chemo-magneto	34.8 (superparamagnetic)	[88]

Composite Type	Synthesis Method	Main Results	Limitations	Application	Magnetization (emu g^{-1})	Ref
U) based on rice straw cellulose matrix with Fe_3O_4 nanofillers	precipitation, Drug Loading	93 % at pH 7.4, 37–44 °C); improved stability, Ms 34.8 emu/g; enhanced anticancer vs. free 5-FU in 2D/3D models; low hemolysis, good biocompatibility	hypoxic penetration, and needs in vivo validation.	theranostics for colorectal cancer		
Polyporous magnetic cellulose beads (PMCBs) modified with diethylenetriamine (DETA) and lipase-immobilized (CALB-PMCBs-DETA)	Coprecipitation (Fe_3O_4), surface activation, amine functionalization, crosslinking, enzyme immobilization	Stable cellulose after Fe_3O_4/enzyme; optimal immobilization (glutaraldehyde 5%, enzyme 15%, pH 7.5, 6 h); catalytic activity 1.0 U/mg; biodiesel yield 92.3% (2h, 60°C); major FAMEs: linoleic/oleic/eicosenoic acids; >85% yield after four cycles, minor loss after 5; easy magnetic recovery	Minor enzyme leaching after 5 uses, needs t-butanol mixing, sensitive to high methanol ratios	Biodiesel production, recyclable enzyme biocatalyst	18.7 (superparamagnetic, rapid recovery)	[89]
Flexible Fe_3O_4 /cellulose composite film (CFe01–CFe03, 0.04–3 wt % Fe_3O_4)	Hydrothermal synthesis; co-dispersion with cellulose ($BzMe_3NOH$); regeneration (sucrose); hot-pressing	Uniform Fe_3O_4 (243 ± 72 nm), tensile strength 49.4 MPa, flexible/foldable, stable to 290 °C, superparamagnetic (Ms 0.5→5.0→27.2), >500 UPF UV shielding, fast/robust dual UV/magnetic sensor	Agglomeration at mid Fe_3O_4 load, mild thermal degradation above 290 °C, aqueous amine solvent needs recycling	Flexible films for UV/magnetic sensing, green electronics, UV shielding	27.2 (CFe03, 3 wt %) (superparamagnetic)	[90]
Melamine-anchored $MnFe_2O_4$ magnetic cellulose ion-imprinted polymer ($MnFe_2O_4$/Cellulose/Melamine IIP)- $MnFe_2O_4$/Cellulose/Melamine IIP	Ultrasonic-assisted sol–gel co-precipitation followed by surface functionalization and ion-imprinted polymer (IIP) synthesis.	Spherical ~50–70 nm; Ms 15.6 emu/g (paramagnetic); optimal pH 8, 18 mg, 13 min (94–98% Cd removal); q_m 138 mg/g (Freundlich, R^2=0.99); selectivity Cd/Co=5, Cd/Ni=12, Cd/Cu=4, Cd/Pb=3; reuse 95% (3 cycles); <6% mass loss, low Fe/Mn leaching; validated for fish/liver/lettuce (94–96% recovery)	Minor core dissolution in acid, limited to 3 cycles, multi-ion system needs optimization	Cd^{2+} preconcentration from food matrices; rapid, reusable magnetic SPE for trace detection	15.6 (rapid paramagnetic).	[91]
Polyethylenimine-functionalized magnetic cellulose nanofibers (PEI-CNFs@Fe_3O_4) from banana peel	Alkali–bleaching pretreatment and acid hydrolysis, followed by Fe_3O_4 chemical co-precipitation and glutaraldehyde-crosslinked PEI surface grafting.	Uniform Fe_3O_4(10–20 nm); PEI crosslinked surface (confirmed via FTIR/XPS); monolayer adsorption (Langmuir, R^2>0.97); q_{max}: Ni^{2+} 134, Cd^{2+} 174, Cu^{2+} 94 mg/g; chemisorption (pseudo-2nd order); spontaneous, endothermic adsorption; >92% reusability (5 cycles); mechanisms: chelation, pH-dependent electrostatic attraction	Moderate Fe_3O_4 limits Ms (<20), slightly lower Cu^{2+} uptake; multi-cycle stability in real wastewater not tested	Magnetic multi-metal adsorbent from banana peel for effluent remediation	18.9 (fast superparamagnetic)	[92]
Fe_3O_4@CMCCOS-EDTA (EDTA-functionalized magnetic chitosan oligosaccharide/carboxymethyl cellulose hybrid)	Fe_3O_4 co-precipitation followed by EDC/NHS-activated EDTA surface grafting.	Uniform ~20 nm Fe_3O_4; EDTA confirmed by strong CO, C–O, Fe–O bands; q_{max} 432.3 mg/g Pb(II) (pH 5, 308 K); fits Langmuir (R^2>0.995) and PSO kinetics; >100% removal <200 mg/L, 54% at 1000 mg/L; >77% adsorption after four cycles; superparamagnetic, fast separation	Magnetization drop after EDTA grafting, partial capacity loss on repeat use, and no multi-ion selectivity study	Efficient Pb(II) removal from water; recyclable magnetic heavy metal adsorbent	Fe_3O_4@CMCCOS: 50.3, Fe_3O_4@CMCCOS-EDTA: 46.8 (superparamagnetic)	[93]
CMC/MGOs (magnetic graphene oxide)	Hummers' GO, Co-precipitation, Plasma Polymerization	Sheet-like, Fe_3O_4 (20–100 nm), 189 mg/g U(VI) uptake, >80% reuse	Some Fe leaching, slow equilibrium	High-capacity, recyclable U(VI) adsorbent	~50 (Fe_3O_4@GO), 45 (CMC-MGO)	[94]
NFC1/NFC2/NFC3 Fe_3O_4–NC comps	Coating, co-precipitation, and formaldehyde cross-linking.	6–25 nm; Pb/Cu (3150/2150), Cd (800 µmol/g); fast adsorption; exothermic biosorption, 100 % removal in seawater (Pb/Cu); high thermal stability	Slight loss in multi-ion, Fe_3O_4 leach under acid, further reusability needed	Efficient eco-remediation, magnetic separation	Fe_3O_4 ≈ 50; comp. ≈ 45, rapid separation	[95]
CNC@$CoFe_2O_4$ hybrid	Co-precipitation	Uniform NP dispersion; ferromagnetism; transparent, flexible films/fibers (~20 emu/g, 8 °C hyperthermia rise)	Some agglomeration, limited magnetic loading (7 %)	Magneto-responsive films/fibers, hyperthermia, shielding	20 (CNC-inorganic); 40 (bulk NP)	[96]
CNC-PCA/Fe_3O_4	Thermal decomposition/grafting	Superparamagnetic, Ms = 52.2, high r_1/r_2, dual MRI contrast, low toxicity	Slight magnetic moment drop after coating, needs in vivo validation	Dual-mode MRI contrast,	52.2 (CNC-PCA/Fe_3O_4), 60 (bare)	[97]

Composite Type	Synthesis Method	Main Results	Limitations	Application	Magnetization (emu g^{-1})	Ref
				biocompatible nanocomposite		
Fe_3O_4@cellulose composite	Co-precipitation	Uniform 20 nm NPs, 95% bacterial capture, rapid separation, variable Ms (3–25)	Lower BET vs bare NPs, non-selective adsorption, untested scaling	Capture/isolation of cellulose-degrading bacteria	3.3–24.9 (ratio-dependent); superparamagnetic	[98]
MBNC (Magnetic Bacterial Nanocellulose)	In situ co-precipitation	Biocompatible, PEG-coated, robust hydrogel, cell recruitment, paramagnetic, no DNA damage	Magnetization not quantified, requires tuning, and has limited in vivo data	Neurovascular stent coatings, tissue regeneration	Paramagnetic (qualitative)	[99]
NCA/OA/Fe_3O_4 aerogel	High-speed mixing	Low-density, high surface area, hydrophobic, excellent organic adsorption, magnetic, recyclable	Fragile after cycles, not all oils tested, magnetization not quantified	Magnetic oil/solvent adsorbent, spill cleanup	10–25 (typical 0.5 wt% % Fe_3O_4)	[100]
Cellulose maleate derivatives cross-linked with divinylbenzene and impregnated with silica-coated Fe_3O_4 nanoparticles (CMD/Fe_3O_4@SiO_2 hydrophobic polymer)	Cross-linking/impregnation	Ultra-fast, high-capacity superhydrophobic oil adsorption, magnetic recyclable, >80% reuse	Efficiency declines after cycles, with slight hysteresis	Oil/solvent removal, magnetic adsorbent	20 (CMD/Fe_3O_4@SiO_2), 30 (Fe_3O_4@SiO_2).	[101]
Fe_3O_4/TiO_2@cellulose aerogel	Co-precipitation/sol-gel	Highly porous, superhydrophobic, rapid oil uptake (28×weight), selective absorption, reusability	Magnetization not reported, needs quant. magnetics, mechanical test for deployment	Selective oil spill removal, magnetic recovery	Not reported	[102]
(Magnetic Cellulose–PEI Crosslinked Composite) – Cellulose crosslinked with polyethyleneimine and Fe_3O_4 nanoparticles (MNPs)-MCPEI (Cellulose–PEI/MNP)	Crosslinking (glutaraldehyde)	330 mg/g capacity for RB5 dye, 99.8 % removal, high anionic selectivity, 74.9 % reuse	Low BET, reduced magnetic response, slight reuse loss	Dye adsorption, magnetic wastewater treatment	3.3 (MCPEI) vs 67.1 (bare MNP)	[103]
FSIL@CP (Fe_3O_4@SiO_2-IL/PEI spheres)	Multi-step grafting/etching	Ultra-high adsorption (1299 mg/g CR, 1068 mg/g MB), rapid selectivity, >95% reuse in 3 cycles, fast magnetic separation (30 s)	Reduced magnetism vs. bare Fe_3O_4, site oxidation on reuse	Magnetic, functionalized adsorbent for dye removal	374.4 (superparamagnetic)	[104]
Magnetic Nanocellulose Composites (MNCCs) – (CNC/CNF/BNC–Fe_3O_4/$CoFe_2O_4$)	In situ/ex situ co-precipitation, sol-gel, hydrothermal, green	Superior dye/metal uptake (Cd^{2+} 344–401 mg/g); rapid, superparamagnetic recovery; biocompatible; mechanical reinforcement	Stability, aggregation, unstandardized synthesis, toxicity	Dye/metal adsorption, catalysis, biomedical, sensing, tissue engineering	25–90 (Fe_3O_4); >100 ($CoFe_2O_4$)	[105]
PVA/CNC-MF, PVA/CNF-MF	Magnetic alignment (weak mT field)	Dense aligned structure, 75–80% ↑ strength, 8× barrier ↑, shelf life ↑ 6 days, hydrophobic, semi-Ra	Partial alignment (weak field), non-magnetic matrix, elasticity	Food packaging, fruit preservation	None (diamagnetic alignment only)	[106]
CNF/Fe_3O_4@ZnO/OTMS–AS aerogel	PAA-assisted loading, silane crosslink, freeze-dry	Superhydrophobic (WCA 157.5°), strong mechanics (96.95 kPa), oil uptake (12–41.9 g/g), >90% reusability, easy magnetic recovery	Slight drop in magnetism (7.34→3.82), strength loss if overloaded, hydrophobic aging	Oil separation, wastewater, eco-cleanup	3.82 (composite) vs 7.34 (bare)	[107]
(Polyethyleneimine-Grafted Cellulose Nanofiber Magnetic Nanocomposite) – PEI-CNF-MNP (Fe_3O_4@SiO_2-PEI-CNF)	Amide coupling, sol-gel, surface amination	q_{max} Pb^{2+} 324.7, Cu^{2+} 501.2, Co^{2+} 393.6 mg/g, rapid recovery (<90 s); superparamagnetic; durable >10 cycles	Magnetization drop (56.8→18.7), aggregation, adsorption aging	Heavy metal removal, rapid aqueous purification	56.8 (Fe_3O_4), 18.7 (composite)	[108]
(Magnetic Grass Nanocellulose) –MGNC (Cyperus grass NC/Fe_3O_4)	Purification, hydrolysis, co-precipitation	Ce(III) removal q_{max} 353 mg/g, 89% removal, R^2>0.99 (Langmuir, PSO), 50–95% seed recovery, negligible aquatic toxicity	Low Ms (0.18), slow separation at large scale, affected by Zn^{2+} co-ions	Rare-earth and heavy metal wastewater, plant-safe bio removal	0.18; Hci=13.15 Oe (ferro)	[109]
Ag–Fe_3O_4@Cels (MCels)	Cellulose sol–gel, in situ Ag–Fe_3O_4 redox/coprecipitation	4-NP reduction in 5 min, >90% activity after nine cycles, mag. separation <10 s, superparamagnetic, mesoporous	Surface loss (inc. with NP density), 10% activity loss (9 uses), <200 °C	Green catalyst, wastewater, fast separable	23.6–45.1 (dose-dependent)	[110]

**Composite Type**	**Synthesis Method**	**Main Results**	**Limitations**	**Application**	**Magnetization (emu g^{-1})**	**Ref**
(Magnetic Cellulose Nanofiber/Polydopamine–Silver Nanocomposite)-MCNF/PDA–Ag	Hydrothermal Fe_3O_4, dopamine PDA coat, in situ AgNPs	Fast reduction (MB, MO, 4-NP), 99.9% MB removal in 10 min, high reusability, 99% E. coli inactivation	Ag/PDA reduces Ms (9.4 emu/g), activity dip at low pH, long-term disinfection data needed.	Multifunctional nano catalyst, wastewater, antimicrobial	13.6 → 11.1 → 9.4	[111]
CS–ONC/IONP Magnetic Patch.	TEMPO oxidation, $NaBH_4$ reduction, solvent casting, and drug loading.	>12% DOX release (1 h, 50 mT), 65% in 28 d, 79.6% melanoma cell death, >42 °C hyperthermia, superparamagnetic films	Lower strength at high IONP, only in vitro, biostability needs in vivo testing	Topical, magnetically triggered chemotherapy + hyperthermia	IONP: 77.7; Film: 9.2–23.3	
NC–Fe_3O_4 Magnetic Nanocrystalline Cellulose.	co-precipitation	83.7% AZT removal (pH 3, 3 h), q_{max}=175.9 mg/g, Freundlich & PSO fit; selective A375 cytotoxicity (~50%), HaCat biocompatibility (~80%)	Ms not quantified; only qualitative, weak at T>500 °C	Antibiotic adsorption, dual anticancer/biocompatibility	Retains mag. up to 400 °C (no values)	[112]
mCNFAP/PMAA – TEMPO-oxidized Arenga pinnata cellulose nanofiber modified with Fe_3O_4 and grafted poly(methacrylic acid) - mCNFAP/PMAA	Alkaline extraction, TEMPO oxidation, co-precipitation, and PMAA grafting.	q_{max} (Langmuir) = 588.2 mg/g (MB); batch: 201; column: 264.7 mg/g; 75% capacity (15 uses); Ms = 15.5	~25% capacity drop (15 cycles); flow-rate limits; only MB dye tested	Rapid and reusable cationic dye adsorption in water; superparamagnetic separation	15.5 (superparamagnetic)	[113]
Fe_3O_4@CNF (60–83 wt%)	Co-precipitation: hot-pressing	Ms = 38.5 (60%)→68.5 emu/g (83%); σDC = 1.7×10^{-10}–1.9×10^{-8} $(\Omega\ cm)^{-1}$; superparamagnetic; flexible, high Ra	Minor conductivity drop >71% Fe_3O_4; surface roughness ↑ at higher Fe_3O_4; tuning needed for device stability	Magneto-electronic semiconductors, sensors, and flexible devices	38.5–68.5 (superparamagnetic)	[114]
CNC/Fe_3O_4 Hybrid	One-pot:hydrolysis solvothermal	33.3 wt% Fe_3O_4; spherical 30 nm NPs on CNCs; Ms = 22.0 (1:75–1:100); superparamagnetic; highly uniform; ~385 °C stability	Carbonization at extended heating; minor α-Fe_2O_3 byproduct; long (30 h) synthesis	Biocatalysis, adsorption, magnetically recoverable biomedical	11.2–22.1 (superparamagnetic)	[115]
CNF/Fe_3O_4/polymer microparticles	Pickering emulsion, polymerization	2.7–3 µm core–shell spheres; Fe_3O_4 core (1.5 wt%), dense CNF shell; Ms ≈ 1.1; rapid magnetic collection; MB adsorption = 0.68 mg g^{-1} (pH 7).	Low Fe_3O_4 limits Ms; tested only for MB dye; needs >10 % Fe_3O_4 for practical Ms	Magnetically recoverable adsorbent; biocompatible; potential MRI/biosensing	1.1 (paramagnetic, stable)	[116]
CNF-C/PEI/MBTex aerogel	Liquid-phase exfoliation, co-precipitation, hydrothermal, cross-linking, CVD hydrophilization	WCA = 151°, density = 38 mg cm^{-3}, rapid magnetic response, oil adsorption = 24.6–77.8 g g^{-1}, >90 % recovery after 20 cycles, >90 % anti-mold	~10 % loss after acid/solvent; low Fe_3O_4 limits Ms; no kinetics fitted	Robust, eco-friendly oil–water separator; magnetic/anti-mold; industrial waste remediation	1.7 (superparamagnetic)	[117]
(Porcine liver esterase (PLE) immobilized on magnetic nanocrystalline cellulose) -PLE@MNCC	Acid hydrolysis, co-precipitation, immobilization	Uniform 9.3 nm Fe_3O_4 on rods; enzyme loading = 803 mg g^{-1}, 45.2 % activity rec.; 83.3 % patulin removal (48 h, 25 mg m/L); 8 cycles → ~50 % active	Enzyme leaching after 8 reuses; reduced rate > 55 °C	Magnetically recoverable enzyme for juice detox, high biocompatibility	MNCC: 37.5; PLE@MNCC: 9.36 (superparamagnetic)	[118]
Fe_3O_4@APC	Sugar beet pulp cellulose, NaOH/HCl activation, Fe_3O_4 NP incorporation	Q_m iodine: APC = 349.2, 10% = 553.2, 20% = 844.6 mg/g; 94.9–98.2% removal, 6 cycles <16% loss; Langmuir/Pseudo-second-order best fit	Slight iron loss after six cycles, not tested for radioactive iodine	Highly efficient magnetic iodine adsorbent for water treatment	53.8 (Fe_3O_4), 32.7 (20 %@APC,superparamagnetic)	[119]
MGCNF Membrane	In situ-co-precipitation, membrane formation	Particle 9–14 nm, Ms = 38.5–68.5, superparamagnetic, catalyzes PMS AOP, RhB removal = 94.9% (300 min), flexible, reusable	Mild RhB degradation rate, PMS activation limited by Fe^{3+}/Fe^{2+} cycling	Dye removal and sulfate radical AOPs; flexible, reusable filter media	38.5–68.5 (superparamagnetic)	[120]
CNC–Fe_3O_4 (CF)	Alkali pretreatment, acid hydrolysis, co-precipitation, suspension blending, and	<50 nm core–shell NPs, Ms ≈ 40 (superparamagnetic), 100 % DE @ 50 °C (no magnet), 90 % DE @ 20 °C (magnet), IFT reduced to 22 mN/m, reusable 4×	~20 % DE drop after four cycles, temp/stirring dependent	Recyclable chemical and magnetic demulsifier for	≈ 40 (superparamagnetic)	[121]

**Composite Type**	**Synthesis Method**	**Main Results**	**Limitations**	**Application**	**Magnetization (emu g^{-1})**	**Ref**
	magnetic separation.			crude oil dewatering		
CNF/Fe_3O_4/FS/AS Aerogel	Aqueous-phase assembly, dual silane surface modification, freeze-casting, freeze-drying, and thermal annealing.	Lamellar/branch structure, 30–40 µm pores, Fe_3O_4 uniformly attached; WCA = 151.9°, sliding < 10°; 10–21.1 g/g oil uptake; >89% retention 10 cycles; 15.13 Ms, fully elastic, minimal mass loss	Slight mass loss after multiple cycles; ultra-light oils may lower efficiency	Magnetically recoverable, eco-friendly oil–water separation, environmental cleanups	15.13 (stable after 50 cycles)	[122]
BC/AA/MN@Fe_3O_4 (Bamboo nanocellulose/acrylic acid/montmorillonite/Fe_3O_4)	TEMPO oxidation, acrylic acid grafting, co-precipitation, and freeze-drying assembly.	Mesoporous, highly selective for MG over MO (97.3% selectivity, 2282.3 mg/g q_{max} for MG), broad pH (4–10), >53.4% reuse after 5 cycles	Ms drop after functionalization (13.32), slow equilibrium (24 h), not for anionics	Selective cationic dye removal, recyclable, magnetic water treatment	13.32 (composite)	[123]
BC/Fe_3O_4/PAA Ferrogel	In situ co-precipitation, in situ acrylic acid radical polymerization	BC: 35±5 nm, Fe_3O_4: 25±3 nm; stretchable (1424%); self-healing (~95% strength); Ms = 49.53 for dried nanocellulose; responsive, GF: 0.7–1.6 (strain 10–120%)	Reduced Ms in hydrated gel, swelling equilibrium (7 h), max performance at 1 wt%	Flexible, healable, magneto-strain sensor for soft robotics and wearables	49.53(magnetic nanocellulose, lower for gel)	[124]
Magnetic porous cellulose molecularly imprinted polymer (MPCMIP)	Solvothermal synthesis, surface molecular imprinting through N-isopropylacrylamide polymer grafting	High porosity, 13.6 m²/g (vs. 6.9 for MIP); selective bisphenol recognition (q_{max} 14–16 mg/g); >96% retained after eight cycles; fast equilibrium (<1 hr); 4.4× higher selectivity, 4× mass transfer over standard MIP; broad pH (1–10), temp (15–40 °C); effective magnetic separation despite reduced Ms	Ms reduced by coating, lower q_{max} vs. some MOF/biocarbon; selectivity limited to bisphenols.	Food/environmental bisphenol enrichment; rapid sample prep for HPLC/UPLC	Not specified	[125]
CNC/$CoFe_2O_4$ film	Evaporation-induced self-assembly (EISA)	Crack-free lamellar films; 20% $CoFe_2O_4$: Ms = 12.96, onset degradation 220.4 °C (vs. 165.1), ε' = 12 (vs. 10), strong absorbance; glucose (process aid)	Lower magnetization than pure ferrite, mostly proof-of-concept work	Magnetoelectric/electronic materials, green magnetic films	12.96 (20 wt% filler)	[126]
Fe_3O_4/cellulose-NH_2/P(NIPAM-co-DMAEA) (cisplatin nanocarrier)	co-precipitation, RAFT polymerization of P(NIPAM-co-DMAEA)	Dual pH/thermal sensitivity, Ms = 15, high cisplatin loading (EE% 74%, q_{max} 999 mg/g), LCST 38°C, 240 nm size, 84% release at pH 5.5/40°C/72 h, Fickian diffusion, stronger selectivity index vs. free cisplatin	Lower Ms due to polymer and bio coatings, only in vitro data, multiplex drug loading not developed	On-demand drug delivery, targeted cancer therapy, magnetic imaging	15 (final hybrid)	[127]
CNCs/N6@Fe_3O_4–CT	Acid hydrolysis, co-precipitation onto CNCs/N6 mixture, and chitosan encapsulation	Highly porous, superparamagnetic (Ms = 26.90), IeP pH 7.9, swelling ratio 168.24 g/g, initial 98% salt rejection, high flux at low pressure, broad pH stability, green and recyclable	Decline in rejection after two h due to swelling; flux and selectivity need long-term optimization	Wastewater/microplastics removal, air filtration, sustainable membranes	26.90	[128]
PU/CNCs/MNPs	Solvent casting, sonication, and compression molding.	Increased storage modulus (glass/rubbery), higher Tg, tough, synergistic CNC+MNP reinforcement, rapid shape recovery (<15s AMF, 40 °C), high fixity (Rf >98%), non-toxic and hemocompatible, stable to 5 cycles	Lower crystallinity, color change, reduced strain at break (higher NPs)	Smart actuators, soft robotics, biomedical shape-memory/trigger devices	3–5 (fraction; absolute Ms not specified)	[129]
MCF (bacterial cellulose)	Co-precipitation, freeze-drying, and pyrolysis.	3D nanofiber, meso/macroporous (222.3 m^2/g), uniform Fe_3O_4, q_{max} = 43.6 mg/g (batch), 67 mg/g (column), 93.2% removal, rapid kinetics (30 min), pseudo-2nd order, Sips/multilayer, efficient, magnetic separation	Moderate q_{max} vs. alternatives, column Y 22–33%, regeneration not demonstrated	Fast magnetic adsorbent for diclofenac, potential for other pharmaceuticals	80.0	[130]
Magnetic nanocellulose (β-galactosidase)	Acid/alkaline hydrolysis, co-precipitation, sonication, and batch adsorption.	~75 nm, 15–16 m^2/g, Ms = 14 (alkaline), 18.6 (acidic); 93–98% immobilization (100 mg/g); optimal pH 4.5–6.5, temp ↑ after immobilization, 1.2–1.6× longer half-life, >30 cycles: >50% hydrolysis, >50% hydrolysis in milk/whey (24h continuous)	Ms falls after immobilization, minor catalytic reduction	Recyclable supports for milk/whey lactose hydrolysis, industrial biocatalysis	7–18	[131]

Composite Type	Synthesis Method	Main Results	Limitations	Application	Magnetization (emu g^{-1})	Ref
Amino acid-modified Fe_3O_4, γ-Fe_2O_3, $MnFe_2O_4$	Solvothermal synthesis and aqueous-phase amino acid adsorption surface functionalization.	Chiral surface (D/L-glutamic acid); enantiospecific peak shift, reversible, enhanced electron transfer (up to 1.9×)	Chiral effect only with Glu/Asp, diminished at high concentration/recycling	Chiral sensors, interfaces, catalysis	Not specified numerically	[132]
GO–CNW hydrogel	Hummer's method, acid hydrolysis, azidation, and covalent click linkage (nitrene insertion).	High MB uptake (34.5–35.3 mg/g), two-site mechanism, >97% efficiency after 3 cycles	Low BET area, nonmagnetic, minor rate loss	Dye removal, water treatment	Not applicable	[133]
Polystyrene macroporous magnetic polyHIPE (Fe_3O_4–polyHIPE)	One-step HIPE polymerization	High porosity, superhydrophobic (CA > 120°), strong magnetic retrieval, 4.15 g/g oil uptake (gasoline), >10× reuse	Not biodegradable, oil uptake ↓ over time, PS-only	Oil spill cleanup	13.5	[134]
Fe_3O_4–P(NIPAM-AM)/CNC hydrogel	Hydrothermal + self-assembly	1D magnetic chain, vivid color shift (494–720 nm, humidity), reversible, fast polymerization	Fe_3O_4 content low, no mechanical/antibacterial data	Humidity/color sensors	Not listed	[135]
Fe_3O_4@PDA/Au/PDA	One-pot LbL assembly	Au-loaded core-shell, ~10-min nitrophenol reduction, >95% after seven cycles, superparamagnetic	Slower than naked Au, not antibacterial	Recyclable catalyst	28.5 (core 47.9)	[136]
Fe_3O_4/CNC@CNCs photonic film	Co-precipitation + EISA under a weak magnetic field	Pitch tunable by weak field, color maintained, Ms = 4.7 at 13.5 wt% Fe_3O_4	Ms much below pure Fe_3O_4, high Fe_3O_4 disrupts the phase	Magnetically-tunable photonics	4.7	[137]
Fe_3O_4@CNC/Cu	Co-precipitation + green Cu reduction	Well-dispersed Fe_3O_4/Cu NPs, high thermal stability, sensitive electrochemical drug sensor, robust magnetic separation	Moderate Ms, application-specific, not for bulk adsorption	Drug sensing, magnetic separation	10.66	[138]

References

1. Arockiasamy, F.S.; Manoharan, B.; Santhi, V.M.; Prakalathan, K.; Periasamy, D.; Dhandapani, A.; Natarajan, V.; Krishnasamy, S.; Thiagamani, S.M.K.; Ilyas, R.A. Navigating the Nano-World Future: Harnessing Cellulose Nanocrystals from Green Sources for Sustainable Innovation. *Heliyon* **2024**, *11*, e41188, doi:10.1016/j.heliyon.2024.e41188.
2. Kallel, F.; Bettaieb, F.; Khiari, R.; García, A.; Bras, J.; Chaabouni, S.E. Isolation and Structural Characterization of Cellulose Nanocrystals Extracted from Garlic Straw Residues. *Industrial Crops and Products* **2016**, *87*, 287–296, doi:10.1016/j.indcrop.2016.04.060.
3. Han, W.; Xu, H.; Qiu, F.; Liu, J.; Gu, H.; Xue, Z. Effect of Oxalic Acid Treatment on the Oxidation Efficiency of Maize Stover Cellulose and Controllable Preparation of Nanocellulose. *RSC Adv.* **2025**, *15*, 2554–2561, doi:10.1039/D4RA08079C.
4. Raza, M.; Abu-Jdayil, B.; Banat, F.; Al-Marzouqi, A.H. Isolation and Characterization of Cellulose Nanocrystals from Date Palm Waste. *ACS Omega* **2022**, *7*, 25366–25379, doi:10.1021/acsomega.2c02333.
5. Promdontree, P.; Kheolamai, P.; Ounkaew, A.; Narain, R.; Ummartyotin, S. Characterization of Cellulose Fiber Derived from Hemp and Polyvinyl Alcohol-Based Composite Hydrogel as a Scaffold Material. *Polymers (Basel)* **2023**, *15*, 4098, doi:10.3390/polym15204098.
6. Sheikh, S.; Hirogaki, K.; Irie, S.; Nakane, K. Preparation and Characterization of Jute Cellulose Nanocrystals Using Tetrabutylammonium Fluoride and Dimethyl Sulfoxide Solutions. *SPE Polymers* **2025**, *6*, e10168, doi:10.1002/pls2.10168.
7. Pirah, S.; Wang, X.; Javed, M.; Simair, K.; Wang, B.; Sui, X.; Lu, C. Lignocellulose Extraction from Sisal Fiber and Its Use in Green Emulsions: A Novel Method. *Polymers (Basel)* **2022**, *14*, 2299, doi:10.3390/polym14112299.
8. Mishra, S.; Prabhakar, B.; Kharkar, P.S.; Pethe, A.M. Banana Peel Waste: An Emerging Cellulosic Material to Extract Nanocrystalline Cellulose. *ACS Omega* **2023**, *8*, 1140–1145, doi:10.1021/acsomega.2c06571.
9. Bigi, F.; Maurizzi, E.; Haghighi, H.; Siesler, H.W.; Licciardello, F.; Pulvirenti, A. Waste Orange Peels as a Source of Cellulose Nanocrystals and Their Use for the Development of Nanocomposite Films. *Foods* **2023**, *12*, 960, doi:10.3390/foods12050960.
10. Nyaruai, C.; Ollengo, D.M.; Muthakia, G. Iridescent Patterns Production from Solid Film Cellulose Nanocrystals Prepared from Coffee Husks. *Univers. J. Carbon Res.* **2024**, 107–123, doi:10.37256/ujcr.2120244851.
11. Claro, A.M.; Dias, I.K.R.; Fontes, M. de L.; Colturato, V.M.M.; Lima, L.R.; Sávio, L.B.; Berto, G.L.; Arantes, V.; Barud, H. da S. Bacterial Cellulose Nanocrystals Obtained through Enzymatic and Acidic Routes: A Comparative Study of Their Main Properties and in Vitro Biological Responses. *Carbohydrate Research* **2024**, *539*, 109104, doi:10.1016/j.carres.2024.109104.
12. Dunlop, M.J.; Clemons, C.; Reiner, R.; Sabo, R.; Agarwal, U.P.; Bissessur, R.; Sojoudiasli, H.; Carreau, P.J.; Acharya, B. Towards the Scalable Isolation of Cellulose Nanocrystals from Tunicates. *Sci Rep* **2020**, *10*, 19090, doi:10.1038/s41598-020-76144-9.
13. Plianwong, S.; Sirirak, T. Cellulose Nanocrystals from Marine Algae *Cladophora Glomerata* by Using Microwave-Assisted Extraction. *International Journal of Biological Macromolecules* **2024**, *260*, 129422, doi:10.1016/j.ijbiomac.2024.129422.
14. Zielińska, D.; Szentner, K.; Waśkiewicz, A.; Borysiak, S. Production of Nanocellulose by Enzymatic Treatment for Application in Polymer Composites. *Materials (Basel)* **2021**, *14*, 2124, doi:10.3390/ma14092124.

15. Hasan, M.J.; Johnson, A.E.; Ureña-Benavides, E.E. “Greener” Chemical Modification of Cellulose Nanocrystals via Oxa-Michael Addition with N-Benzylmaleimide. *Curr. Res. Green Sustain. Chem.* **2021**, *4*, 100081, doi:10.1016/j.crgsc.2021.100081.
16. Lin, K.-H.; Enomae, T.; Chang, F.-C. Cellulose Nanocrystal Isolation from Hardwood Pulp Using Various Hydrolysis Conditions. *Molecules* **2019**, *24*, 3724, doi:10.3390/molecules24203724.
17. Birgani, S.A.; Talaeipour, M.; Hemmasi, A.H.; Bazyar, B.; Larijani, K. Production of Nanocrystalline Cellulose from Bleached Soda Bagasse Pulp. *BioRes* **2021**, *16*, 7817–7829, doi:10.15376/biores.16.4.7817-7829.
18. Babaei-Ghazvini, A.; Patel, R.; Vafakish, B.; McAlpine, S.; Acharya, B. One-Pot Catalytic Isolation of Cellulose Nanocrystals from Agricultural Biomass - Oat Hull, Wheat Straw, and Flax Straw: Physicochemical Characterization. *Bioresour Technol* **2025**, *424*, 132271, doi:10.1016/j.biortech.2025.132271.
19. Wang, L.; Li, Y.; Ye, L.; Zhi, C.; Zhang, T.; Miao, M. Unveiling Structure and Performance of Tea-Derived Cellulose Nanocrystals. *International Journal of Biological Macromolecules* **2024**, *270*, 132117, doi:10.1016/j.ijbiomac.2024.132117.
20. da Costa, F.A.T.; Dufresne, A.; Song, T.; Parra, D.F. Exploring Acid Hydrolysis Conditions and Extended Mechanical Processing for Producing Cellulose Nanocrystal and Nanofibrils from Pineapple Leaf Fibers. *International Journal of Biological Macromolecules* **2025**, *306*, 141755, doi:10.1016/j.ijbiomac.2025.141755.
21. Hassan, Md.M.; Rahman, Md.M.; Ghos, B.C.; Hossain, Md.I.; Amin, Md.A.; Zuhanee, Md.K.A. Extraction, and Characterization of CNC from Waste Sugarcane Leaf Sheath as a Reinforcement of Multifunctional Bio-Nanocomposite Material: A Waste to Wealth Approach. *Carbon Trends* **2024**, *17*, 100400, doi:10.1016/j.cartre.2024.100400.
22. Islam, Md.H.; Hosna Ara, M.; Khan, M.A.; Naime, J.; Khan, Md.A.R.; Rahman, Md.L.; Ruhane, T.A. Preparation of Cellulose Nanocrystals Biofilm from Coconut Coir as an Alternative Source of Food Packaging Material. *ACS Omega* **2025**, *10*, 8960–8970, doi:10.1021/acsomega.4c06400.
23. Juárez Méndez, M.E.; Palma Ramírez, D.; García Zaleta, D.S.; Neri Espinoza, K.A.; López Benítez, A.; del Ángel López, D.; Morales García, S.S.; Willcock, H. A Strategy Towards the Valorization of Aloe Vera Rinds to Obtain Crystalline Cellulose: Pretreatment Effects and Elemental Analysis. *Polymers (Basel)* **2025**, *17*, 553, doi:10.3390/polym17040553.
24. Zambrano-Mite, L.F.; Villasana, Y.; Bejarano, M.L.; Luciani, C.; Niebieskikwiat, D.; Álvarez, W.; Cueva, D.F.; Aguilera-Pesantes, D.; Orejuela-Escobar, L.M. Optimization of Microfibrillated Cellulose Isolation from Cocoa Pod Husk via Mild Oxalic Acid Hydrolysis: A Response Surface Methodology Approach. *Heliyon* **2023**, *9*, e17258, doi:10.1016/j.heliyon.2023.e17258.
25. Sadare, O.O.; Mabunda, N.; Ikegwu, U.M.; Keitemoge, M.K.; Daramola, M.O.; Moothi, K. Parametric Optimization of the Production of Cellulose Nanocrystals (CNCs) from South African Corncobs via an Empirical Modelling Approach. *Scientific Reports* **2022**, *12*, 18665, doi:10.1038/s41598-022-22865-y.
26. Chieng, B.W.; Lee, S.H.; Ibrahim, N.A.; Then, Y.Y.; Loo, Y.Y. Isolation and Characterization of Cellulose Nanocrystals from Oil Palm Mesocarp Fiber. *Polymers* **2017**, *9*, 355, doi:10.3390/polym9080355.
27. Paul, S.; Dutta, A. Challenges and Opportunities of Lignocellulosic Biomass for Anaerobic Digestion. *Resources, Conservation and Recycling* **2018**, *130*, 164–174, doi:10.1016/j.resconrec.2017.12.005.

28. Ramos, M.; Laveriano, E.; San Sebastián, L.; Perez, M.; Jiménez, A.; Lamuela-Raventos, R.M.; Garrigós, M.C.; Vallverdú-Queralt, A. Rice Straw as a Valuable Source of Cellulose and Polyphenols: Applications in the Food Industry. *Trends in Food Science & Technology* **2023**, *131*, 14–27, doi:10.1016/j.tifs.2022.11.020.
29. Sartika, D.; Firmansyah, A.P.; Junais, I.; Arnata, I.W.; Fahma, F.; Firmanda, A. High Yield Production of Nanocrystalline Cellulose from Corn Cob through a Chemical-Mechanical Treatment under Mild Conditions. *International Journal of Biological Macromolecules* **2023**, *240*, 124327, doi:10.1016/j.ijbiomac.2023.124327.
30. Collazo-Bigliardi, S.; Ortega-Toro, R.; Chiralt Boix, A. Isolation and Characterisation of Microcrystalline Cellulose and Cellulose Nanocrystals from Coffee Husk and Comparative Study with Rice Husk. *Carbohydrate Polymers* **2018**, *191*, 205–215, doi:10.1016/j.carbpol.2018.03.022.
31. Moradi, E.; Fathi, M. Production of Cellulose Nanocrystals from Tomato Pomace as a Food Waste and Their Application for Stabilizing of Pickering Emulsions. *Bioactive Carbohydrates and Dietary Fibre* **2023**, *30*, 100378, doi:10.1016/j.bcdf.2023.100378.
32. Fortunati, E.; Benincasa, P.; Balestra, G.M.; Luzi, F.; Mazzaglia, A.; Del Buono, D.; Puglia, D.; Torre, L. Revalorization of Barley Straw and Husk as Precursors for Cellulose Nanocrystals Extraction and Their Effect on PVA_CH Nanocomposites. *Industrial Crops and Products* **2016**, *92*, 201–217, doi:10.1016/j.indcrop.2016.07.047.
33. Zhu, S.; Sun, H.; Mu, T.; Li, Q.; Richel, A. Preparation of Cellulose Nanocrystals from Purple Sweet Potato Peels by Ultrasound-Assisted Maleic Acid Hydrolysis. *Food Chemistry* **2023**, *403*, 134496, doi:10.1016/j.foodchem.2022.134496.
34. Ebrahimi, R.; Fathi, M.; Ghoddusi, H.B. Nanoencapsulation of Oregano Essential Oil Using Cellulose Nanocrystals Extracted from Hazelnut Shell to Enhance Shelf Life of Fruits: Case Study: Pears. *International Journal of Biological Macromolecules* **2023**, *242*, 124704, doi:10.1016/j.ijbiomac.2023.124704.
35. Trilokesh, C.; Uppuluri, K.B. Isolation and Characterization of Cellulose Nanocrystals from Jackfruit Peel. *Scientific Reports* **2019**, *9*, 16709, doi:10.1038/s41598-019-53412-x.
36. Liu, Q.; Yuan, T.; Fu, Q.; Bai, Y.; Peng, F.; Yao, C. Choline Chloride-Lactic Acid Deep Eutectic Solvent for Delignification and Nanocellulose Production of Moso Bamboo. *Cellulose* **2019**, *26*, 9447–9462, doi:10.1007/s10570-019-02726-0.
37. Xu, K.; Li, Q.; Xie, L.; Shi, Z.; Su, G.; Harper, D.; Tang, Z.; Zhou, J.; Du, G.; Wang, S. Novel Flexible, Strong, Thermal-Stable, and High-Barrier Switchgrass-Based Lignin-Containing Cellulose Nanofibrils/Chitosan Biocomposites for Food Packaging. *Industrial Crops and Products* **2022**, *179*, 114661, doi:10.1016/j.indcrop.2022.114661.
38. Guo, Y.; Zhang, Y.; Zheng, D.; Li, M.; Yue, J. Isolation and Characterization of Nanocellulose Crystals via Acid Hydrolysis from Agricultural Waste-Tea Stalk. *International Journal of Biological Macromolecules* **2020**, *163*, 927–933, doi:10.1016/j.ijbiomac.2020.07.009.
39. Bahloul, A.; Semlali, F.-Z.; Oumam, M.; Hannache, H.; Kassab, Z.; El Achaby, M. Starch Bio-Nanocomposites Based on Phosphorylated and Sulphated Cellulose Nanocrystals Extracted from Pepper Plant Residue: Effect of Surface Functionality on Property Improvements. *Cellulose* **2023**, *30*, 5051–5070, doi:10.1007/s10570-023-05199-4.
40. Ditzel, F.I.; Prestes, E.; Carvalho, B.M.; Demiate, I.M.; Pinheiro, L.A. Nanocrystalline Cellulose Extracted from Pine Wood and Corncob. *Carbohydrate Polymers* **2017**, *157*, 1577–1585, doi:10.1016/j.carbpol.2016.11.036.
41. Bououdina, M.; Alwqyan, T.S.; Khezami, L.; Al-Najar, B.; Shaikh, M.N.; Gill, R.; Modwi, A.; Taha, K.K.; Lemine, O.M. Fabrication and Characterization of Nanostructured MgO·Fe2O3

Composite by Mechanical Milling as Efficient Adsorbent of Heavy Metals. *Journal of Alloys and Compounds* **2019**, *772*, 1030–1039, doi:10.1016/j.jallcom.2018.09.010.
42. Ali, A.; Shah, T.; Ullah, R.; Zhou, P.; Guo, M.; Ovais, M.; Tan, Z.; Rui, Y. Review on Recent Progress in Magnetic Nanoparticles: Synthesis, Characterization, and Diverse Applications. *Front. Chem.* **2021**, *9*, doi:10.3389/fchem.2021.629054.
43. Alonso, J.; Barandiarán, J.M.; Fernández Barquín, L.; García-Arribas, A. Chapter 1 - Magnetic Nanoparticles, Synthesis, Properties, and Applications. In *Magnetic Nanostructured Materials*; El-Gendy, A.A., Barandiarán, J.M., Hadimani, R.L., Eds.; Micro and Nano Technologies; Elsevier, 2018; pp. 1–40 ISBN 978-0-12-813904-2.
44. Song, K.; Kim, W.; Suh, C.-Y.; Shin, D.; Ko, K.-S.; Ha, K. Magnetic Iron Oxide Nanoparticles Prepared by Electrical Wire Explosion for Arsenic Removal. *Powder Technology* **2013**, *246*, 572–574, doi:10.1016/j.powtec.2013.06.023.
45. Wang, Y.; Pan, J.-A.; Wu, H.; Talapin, D.V. Direct Wavelength-Selective Optical and Electron-Beam Lithography of Functional Inorganic Nanomaterials. *ACS Nano* **2019**, *13*, 13917–13931, doi:10.1021/acsnano.9b05491.
46. Reddy, D.H.K.; Lee, S.-M. Application of Magnetic Chitosan Composites for the Removal of Toxic Metal and Dyes from Aqueous Solutions. *Advances in Colloid and Interface Science* **2013**, *201–202*, 68–93, doi:10.1016/j.cis.2013.10.002.
47. Grammatikopoulos, P.; Steinhauer, S.; Vernieres, J.; Singh, V.; Sowwan, M. Nanoparticle Design by Gas-Phase Synthesis. *Advances in Physics: X* **2016**, *1*, 81–100, doi:10.1080/23746149.2016.1142829.
48. Hammad, M.; Hardt, S.; Mues, B.; Salamon, S.; Landers, J.; Slabu, I.; Wende, H.; Schulz, C.; Wiggers, H. Gas-Phase Synthesis of Iron Oxide Nanoparticles for Improved Magnetic Hyperthermia Performance. *Journal of Alloys and Compounds* **2020**, *824*, 153814, doi:10.1016/j.jallcom.2020.153814.
49. Gorylewski, D.; Tyszczuk-Rotko, K. From Synthesis to Sensing: The Insight into the Properties of Fe3O4 Magnetic Nanoparticles and Their Surface Modification Strategies in Voltammetric Trace Determination of Heavy Metal Ions. *Molecules* **2025**, *30*, 3796, doi:10.3390/molecules30183796.
50. Stiufiuc, G.F.; Stiufiuc, R.I. Magnetic Nanoparticles: Synthesis, Characterization, and Their Use in Biomedical Field. *Applied Sciences* **2024**, *14*, 1623, doi:10.3390/app14041623.
51. Majidi, S.; Zeinali Sehrig, F.; Farkhani, S.M.; Soleymani Goloujeh, M.; Akbarzadeh, A. Current Methods for Synthesis of Magnetic Nanoparticles. *Artificial Cells, Nanomedicine, and Biotechnology* **2016**, *44*, 722–734, doi:10.3109/21691401.2014.982802.
52. García, M.A.; Bouzas, V.; Costo, R.; Veintemillas, S.; Morales, P.; García-Hernández, M.; Alexandrescu, R.; Morjan, I.; Gasco, P. Magnetic Properties of Fe Oxide Nanoparticles Produced by Laser Pyrolysis for Biomedical Applications. *AIP Conference Proceedings* **2010**, *1275*, 26–29, doi:10.1063/1.3505076.
53. Jahid Hasan, M.; Westphal, E.; Chen, P.; Saini, A.; Chu, I.-W.; J. Watzman, S.; Ureña-Benavides, E.; S. Vasquez, E. Adsorptive Properties and On-Demand Magnetic Response of lignin@Fe 3 O 4 Nanoparticles at Castor Oil–Water Interfaces. *RSC Adv.* **2023**, *13*, 2768–2779, doi:10.1039/D2RA07952F.
54. Schemberg, J.; Abbassi, A.E.; Lindenbauer, A.; Chen, L.-Y.; Grodrian, A.; Nakos, X.; Apte, G.; Khan, N.; Kraupner, A.; Nguyen, T.-H.; et al. Synthesis of Biocompatible Superparamagnetic Iron Oxide Nanoparticles (SPION) under Different Microfluidic Regimes. *ACS Appl. Mater. Interfaces* **2022**, *14*, 48011–48028, doi:10.1021/acsami.2c13156.

55. Wroblewski, C.; Volford, T.; Martos, B.; Samoluk, J.; Martos, P. High Yield Synthesis and Application of Magnetite Nanoparticles (Fe3O4). *Magnetochemistry* **2020**, *6*, 22, doi:10.3390/magnetochemistry6020022.
56. Gutierrez, F.V.; Lima, I.S.; De Falco, A.; Ereias, B.M.; Baffa, O.; Diego de Abreu Lima, C.; Morais Sinimbu, L.I.; de la Presa, P.; Luz-Lima, C.; Damasceno Felix Araujo, J.F. The Effect of Temperature on the Synthesis of Magnetite Nanoparticles by the Coprecipitation Method. *Heliyon* **2024**, *10*, e25781, doi:10.1016/j.heliyon.2024.e25781.
57. Yusuf, M.S.; Sutriyo; Rahmasari, R. Synthesis Processing Condition Optimization of Citrate Stabilized Superparamagnetic Iron Oxide Nanoparticles Using Direct Co-Precipitation Method. *Biomedical and Pharmacology Journal* **2021**, *14*, 1533–1542.
58. Martinez de la Torre, C.; Bennewitz, M.F. Manganese Oxide Nanoparticle Synthesis by Thermal Decomposition of Manganese(II) Acetylacetonate. *J Vis Exp* **2020**, doi:10.3791/61572.
59. Unni, M.; Uhl, A.; Savliwala, S.; Savitzky, B.H.; Dhavalikar, R.; Garraud, N.; Arnold, D.P.; Kourkoutis, L.F.; Andrew, J.; Rinaldi, C. Thermal Decomposition Synthesis of Iron Oxide Nanoparticles with Diminished Magnetic Dead Layer by Controlled Addition of Oxygen. *ACS Nano* **2017**, *11*, 2284–2303, doi:10.1021/acsnano.7b00609.
60. Alkilany, A.M.; Murphy, C.J. Toxicity and Cellular Uptake of Gold Nanoparticles: What We Have Learned so Far? *J Nanopart Res* **2010**, *12*, 2313–2333, doi:10.1007/s11051-010-9911-8.
61. Chen, S.; Xu, Y.; He, X.; Su, Y.; Yang, J.; Chen, W.; Tan, H. Microemulsion Synthesis of Nanosized Calcium Sulfate Hemihydrate and Its Morphology Control by Different Surfactants. *ACS Omega* **2019**, *4*, 9552–9556, doi:10.1021/acsomega.9b00797.
62. Rehman, Z.U.; Nawaz, M.; Ullah, H.; Uddin, I.; Shad, S.; Eldin, E.; Alshgari, R.A.; Bahajjaj, A.A.A.; Arifeen, W.U.; Javed, M.S. Synthesis and Characterization of Ni Nanoparticles via the Microemulsion Technique and Its Applications for Energy Storage Devices. *Materials* **2023**, *16*, 325, doi:10.3390/ma16010325.
63. Abd, M.A.; Abd, A.M. Study a Structural and Magnetic Properties of a Hematite (αFe2O3) Nanoparticles Synthesized by Hydrothermal Method. *AIP Conference Proceedings* **2024**, *3219*, 060009, doi:10.1063/5.0236250.
64. Mora-Cabello, R.; Fuentes-Ríos, D.; Gago, L.; Cabeza, L.; Moscoso, A.; Melguizo, C.; Prados, J.; Sarabia, F.; López-Romero, J.M. Magnetic Nanoparticles with On-Site Azide and Alkyne Functionalized Polymer Coating in a Single Step through a Solvothermal Process. *Pharmaceutics* **2024**, *16*, 1226, doi:10.3390/pharmaceutics16091226.
65. Dudchenko, N.; Pawar, S.; Perelshtein, I.; Fixler, D. Magnetite Nanoparticles: Synthesis and Applications in Optics and Nanophotonics. *Materials (Basel)* **2022**, *15*, 2601, doi:10.3390/ma15072601.
66. Shabelskaya, N.; Sulima, S.; Sulima, E.; Medennikov, O.; Kulikova, M.; Kolesnikova, T.; Sushkova, S. Study of the Possibility of Using Sol–Gel Technology to Obtain Magnetic Nanoparticles Based on Transition Metal Ferrites. *Gels* **2023**, *9*, 217, doi:10.3390/gels9030217.
67. Jesus, J.; Regadas, J.; Costa, B.; Carvalho, J.; Pádua, A.; Henriques, C.; Soares, P.I.P.; Gavinho, S.; Valente, M.A.; Graça, M.P.F.; et al. Green Sol–Gel Synthesis of Iron Oxide Nanoparticles for Magnetic Hyperthermia Applications. *Pharmaceutics* **2024**, *16*, 1578, doi:10.3390/pharmaceutics16121578.
68. Iacovita, C.; Fizeșan, I.; Pop, A.; Scorus, L.; Dudric, R.; Stiufiuc, G.; Vedeanu, N.; Tetean, R.; Loghin, F.; Stiufiuc, R.; et al. In Vitro Intracellular Hyperthermia of Iron Oxide Magnetic Nanoparticles, Synthesized at High Temperature by a Polyol Process. *Pharmaceutics* **2020**, *12*, 424, doi:10.3390/pharmaceutics12050424.

69. Wang, J.; Hou, Y. Iron Carbide Nanostructures: An Emerging Material for Tumor Theranostics. *Acc. Mater. Res.* **2022**, *3*, 89–99, doi:10.1021/accountsmr.0c00018.
70. Baričić, M.; Nuñez, J.M.; Aguirre, M.H.; Hrabovsky, D.; Seydou, M.; Meneghini, C.; Peddis, D.; Ammar, S. Advancements in Polyol Synthesis: Expanding Chemical Horizons and Néel Temperature Tuning of CoO Nanoparticles. *Sci Rep* **2024**, *14*, 12529, doi:10.1038/s41598-024-54892-2.
71. Kustov, L.; Vikanova, K. Synthesis of Metal Nanoparticles under Microwave Irradiation: Get Much with Less Energy. *Metals* **2023**, *13*, 1714, doi:10.3390/met13101714.
72. Wang, S.; Hou, Y. New Types of Magnetic Nanoparticles for Stimuli-Responsive Theranostic Nanoplatforms. *Advanced Science* **2024**, *11*, 2305459, doi:10.1002/advs.202305459.
73. Tu, Q.; Poerschke, D.L.; Kortshagen, U.R. Nonthermal Plasma Synthesis of Metallic Ti Nanocrystals. *Nanomaterials* **2024**, *14*, 264, doi:10.3390/nano14030264.
74. Ahmadi, S.; Fazilati, M.; Nazem, H.; Mousavi, S.M. Green Synthesis of Magnetic Nanoparticles Using Satureja Hortensis Essential Oil toward Superior Antibacterial/Fungal and Anticancer Performance. *BioMed Research International* **2021**, *2021*, 8822645, doi:10.1155/2021/8822645.
75. Chand, K.; Cao, D.; Eldin Fouad, D.; Hussain Shah, A.; Qadeer Dayo, A.; Zhu, K.; Nazim Lakhan, M.; Mehdi, G.; Dong, S. Green Synthesis, Characterization and Photocatalytic Application of Silver Nanoparticles Synthesized by Various Plant Extracts. *Arabian Journal of Chemistry* **2020**, *13*, 8248–8261, doi:10.1016/j.arabjc.2020.01.009.
76. Kulkarni, D.; Sherkar, R.; Shirsathe, C.; Sonwane, R.; Varpe, N.; Shelke, S.; More, M.P.; Pardeshi, S.R.; Dhaneshwar, G.; Junnuthula, V.; et al. Biofabrication of Nanoparticles: Sources, Synthesis, and Biomedical Applications. *Front. Bioeng. Biotechnol.* **2023**, *11*, doi:10.3389/fbioe.2023.1159193.
77. Bhardwaj, B.; Singh, P.; Kumar, A.; Kumar, S.; Budhwar, V. Eco-Friendly Greener Synthesis of Nanoparticles. *Adv Pharm Bull* **2020**, *10*, 566–576, doi:10.34172/apb.2020.067.
78. Salari, N.; M.A.Tehrani, R.; Motamedi, M. Zeolite Modification with Cellulose Nanofiber/Magnetic Nanoparticles for the Elimination of Reactive Red 198. *International Journal of Biological Macromolecules* **2021**, *176*, 342–351, doi:10.1016/j.ijbiomac.2021.01.219.
79. Helmiyati, H.; Yunarti, R.T.; Dini, F.W. Magnetic Alginate–Carboxymethyl Cellulose to Immobilize Copper Nanoparticles as a Green and Sustainable Catalyst for 4-Nitrophenol Reduction. *Heliyon* **2023**, *9*, e14111, doi:10.1016/j.heliyon.2023.e14111.
80. Sun, J.; Cui, L.; Gao, Y.; He, Y.; Liu, H.; Huang, Z. Environmental Application of Magnetic Cellulose Derived from *Pennisetum Sinese* Roxb for Efficient Tetracycline Removal. *Carbohydrate Polymers* **2021**, *251*, 117004, doi:10.1016/j.carbpol.2020.117004.
81. Dai, H.; Huang, Y.; Zhang, Y.; Zhang, H.; Huang, H. Green and Facile Fabrication of Pineapple Peel Cellulose/Magnetic Diatomite Hydrogels in Ionic Liquid for Methylene Blue Adsorption. *Cellulose* **2019**, *26*, 3825–3844, doi:10.1007/s10570-019-02283-6.
82. Zhou, S.; Xia, L.; Fu, Z.; Zhang, C.; Duan, X.; Zhang, S.; Wang, Y.; Ding, C.; Liu, X.; Xu, W. Purification of Dye-Contaminated Ethanol-Water Mixture Using Magnetic Cellulose Powders Derived from Agricultural Waste Biomass. *Carbohydrate Polymers* **2021**, *258*, 117690, doi:10.1016/j.carbpol.2021.117690.
83. Li, B.; Zhang, Q.; Pan, Y.; Li, Y.; Huang, Z.; Li, M.; Xiao, H. Functionalized Porous Magnetic Cellulose/Fe3O4 Beads Prepared from Ionic Liquid for Removal of Dyes from Aqueous Solution. *International Journal of Biological Macromolecules* **2020**, *163*, 309–316, doi:10.1016/j.ijbiomac.2020.06.280.

84. Rabbi, M.A.; Rahman, M.M.; Minami, H.; Yamashita, N.; Habib, M.R.; Ahmad, H. Magnetically Responsive Antibacterial Nanocrystalline Jute Cellulose Nanocomposites with Moderate Catalytic Activity. *Carbohydrate Polymers* **2021**, *251*, 117024, doi:10.1016/j.carbpol.2020.117024.
85. Karzar Jeddi, M.; Laitinen, O.; Liimatainen, H. Magnetic Superabsorbents Based on Nanocellulose Aerobeads for Selective Removal of Oils and Organic Solvents. *Materials & Design* **2019**, *183*, 108115, doi:10.1016/j.matdes.2019.108115.
86. He, X.; Chen, T.; Jiang, T.; Wang, C.; Luan, Y.; Liu, P.; Liu, Z. Preparation and Adsorption Properties of Magnetic Hydrophobic Cellulose Aerogels Based on Refined Fibers. *Carbohydrate Polymers* **2021**, *260*, 117790, doi:10.1016/j.carbpol.2021.117790.
87. He, X.; Liu, Q.; Xu, Z. Cellulose-Coated Magnetic Janus Nanoparticles for Dewatering of Crude Oil Emulsions. *Chemical Engineering Science* **2021**, *230*, 116215, doi:10.1016/j.ces.2020.116215.
88. Yusefi, M.; Lee-Kiun, M.S.; Shameli, K.; Teow, S.-Y.; Ali, R.R.; Siew, K.-K.; Chan, H.-Y.; Wong, M.M.-T.; Lim, W.-L.; Kuča, K. 5-Fluorouracil Loaded Magnetic Cellulose Bionanocomposites for Potential Colorectal Cancer Treatment. *Carbohydrate Polymers* **2021**, *273*, 118523, doi:10.1016/j.carbpol.2021.118523.
89. Zhang, H.; Liu, T.; Zhu, Y.; Hong, L.; Li, T.; Wang, X.; Fu, Y. Lipases Immobilized on the Modified Polyporous Magnetic Cellulose Support as an Efficient and Recyclable Catalyst for Biodiesel Production from *Yellow Horn* Seed Oil. *Renewable Energy* **2020**, *145*, 1246–1254, doi:10.1016/j.renene.2019.06.031.
90. Yang, W.; Tian, H.; Liao, J.; Wang, Y.; Liu, L.; Zhang, L.; Lu, A. Flexible and Strong Fe3O4/Cellulose Composite Film as Magnetic and UV Sensor. *Applied Surface Science* **2020**, *507*, 145092, doi:10.1016/j.apsusc.2019.145092.
91. Hassan Amini, M.; Alijani, H.; Hossein Beyki, M. Toxic Cadmium Selective Sequestration from Food Samples Using Melamine Anchored Magnetic Cellulose by Surface Imprinting Route. *Food Chemistry* **2022**, *396*, 133688, doi:10.1016/j.foodchem.2022.133688.
92. Guo, Z.; Yang, R.; Yang, F.; Sun, L.; Li, Y.; Xu, J. Fabrication of Polyethylenimine Functionalized Magnetic Cellulose Nanofibers for the Sorption of Ni(II), Cu(II) and Cd(II) in Single-Component and Multi-Component Systems. *International Journal of Biological Macromolecules* **2021**, *184*, 68–78, doi:10.1016/j.ijbiomac.2021.06.041.
93. Lian, Z.; Li, Y.; Xian, H.; Ouyang, X.; Lu, Y.; Peng, X.; Hu, D. EDTA-Functionalized Magnetic Chitosan Oligosaccharide and Carboxymethyl Cellulose Nanocomposite: Synthesis, Characterization, and Pb(II) Adsorption Performance. *International Journal of Biological Macromolecules* **2020**, *165*, 591–600, doi:10.1016/j.ijbiomac.2020.09.156.
94. Zong, P.; Cao, D.; Cheng, Y.; Wang, S.; Zhang, J.; Guo, Z.; Hayat, T.; Alharbi, N.S.; He, C. Carboxymethyl Cellulose Supported Magnetic Graphene Oxide Composites by Plasma Induced Technique and Their Highly Efficient Removal of Uranium Ions. *Cellulose* **2019**, *26*, 4039–4060, doi:10.1007/s10570-019-02358-4.
95. El Nemr, A.; El-Assal, A.A.M.; El Sikaily, A.; Mahmoud, M.E.; Amira, M.F.; Ragab, S. New Magnetic Cellulose Nanobiocomposites for Cu(II), Cd(II) and Pb(II) Ions Removal: Kinetics, Thermodynamics and Analytical Evaluation. *Nanotechnol. Environ. Eng.* **2021**, *6*, 42, doi:10.1007/s41204-021-00138-9.
96. Nypelö, T.; Rodriguez-Abreu, C.; Rivas, J.; Dickey, M.D.; Rojas, O.J. Magneto-Responsive Hybrid Materials Based on Cellulose Nanocrystals. *Cellulose* **2014**, *21*, 2557–2566, doi:10.1007/s10570-014-0307-2.

97. Torkashvand, N.; Sarlak, N. Fabrication of a Dual T1 and T2 Contrast Agent for Magnetic Resonance Imaging Using Cellulose Nanocrystals/Fe3O4 Nanocomposite. *European Polymer Journal* **2019**, *118*, 128–136, doi:10.1016/j.eurpolymj.2019.05.048.
98. Zhao, X.; Li, H.; Ding, A.; Zhou, G.; Sun, Y.; Zhang, D. Preparing and Characterizing Fe3O4@cellulose Nanocomposites for Effective Isolation of Cellulose-Decomposing Microorganisms. *Materials Letters* **2016**, *163*, 154–157, doi:10.1016/j.matlet.2015.10.061.
99. Echeverry-Rendon, M.; Reece, L.M.; Pastrana, F.; Arias, S.L.; Shetty, A.R.; Pavón, J.J.; Allain, J.P. Bacterial Nanocellulose Magnetically Functionalized for Neuro-Endovascular Treatment. *Macromolecular Bioscience* **2017**, *17*, 1600382, doi:10.1002/mabi.201600382.
100. Gu, H.; Zhou, X.; Lyu, S.; Pan, D.; Dong, M.; Wu, S.; Ding, T.; Wei, X.; Seok, I.; Wei, S.; et al. Magnetic Nanocellulose-Magnetite Aerogel for Easy Oil Adsorption. *Journal of Colloid and Interface Science* **2020**, *560*, 849–856, doi:10.1016/j.jcis.2019.10.084.
101. Omidi, A.; Firoozbakht, F.; Zali Boeini, H.; Varma, R.S. Magnetic Hydrophobic Cellulose Nanocomposites for Efficient Removal of Oil Spills and Organic Solvents. *Energy Fuels* **2024**, *38*, 23367–23376, doi:10.1021/acs.energyfuels.4c04232.
102. Chin, S.F.; Binti Romainor, A.N.; Pang, S.C. Fabrication of Hydrophobic and Magnetic Cellulose Aerogel with High Oil Absorption Capacity. *Materials Letters* **2014**, *115*, 241–243, doi:10.1016/j.matlet.2013.10.061.
103. Nordin, A.H.; Wong, S.; Ngadi, N.; Mohammad Zainol, M.; Abd Latif, N.A.F.; Nabgan, W. Surface Functionalization of Cellulose with Polyethyleneimine and Magnetic Nanoparticles for Efficient Removal of Anionic Dye in Wastewater. *Journal of Environmental Chemical Engineering* **2021**, *9*, 104639, doi:10.1016/j.jece.2020.104639.
104. Ling, C.; Yimin, D.; Qi, L.; Chengqian, F.; Zhiheng, W.; Yaqi, L.; Ling, C.; Bo, L.; Yue-Fei, Z.; Yan, L.; et al. Novel High-Efficiency Adsorbent Consisting of Magnetic Cellulose-Based Ionic Liquid for Removal of Anionic Dyes. *Journal of Molecular Liquids* **2022**, *353*, 118723, doi:10.1016/j.molliq.2022.118723.
105. Shaker, K.; Jabbar, M.; Awais, H.; Abbas, A.; Nordin, A.H.; Ilyas, R.A.; Khoo, P.S.; Suryanegara, L. Synthesis, Properties, and Environmental Applications of Magnetic Nanocellulose Composites. *Journal of Environmental Chemical Engineering* **2025**, *13*, 119494, doi:10.1016/j.jece.2025.119494.
106. Li, L.; Wang, W.; Sun, J.; Chen, Z.; Ma, Q.; Ke, H.; Yang, J. Improved Properties of Polyvinyl Alcohol Films Blended with Aligned Nanocellulose Particles Induced by a Magnetic Field. *Food Packaging and Shelf Life* **2022**, *34*, 100985, doi:10.1016/j.fpsl.2022.100985.
107. Shi, L.; Yu, W.; Wang, E.; Han, W.; Miao, Y.; Liang, Y.; Chen, Y.; Zhang, W.; Zhang, R.; Huang, J. Designing Magnetic and Superhydrophobic Cellulose Nanofibers Based-Aerogel for Efficient Oil Water Separation. *Journal of Polymer Science* **2024**, *62*, 1853–1863, doi:10.1002/pol.20230871.
108. Sathasivam, T.; Kang Brian, L.; Andersen, I.M.; Ru Tan, H.; Zhang, Z.; Wu, T.; Hong Lau, H.; Zhu, Q.; Kai, D. Green Nanocellulose/PEI-Grafted Magnetic Nanoparticles for Effective Removal of Heavy Metal Ions. *Chemistry – An Asian Journal* **2023**, *18*, e202300842, doi:10.1002/asia.202300842.
109. Shahnaz, T.; Vishnu Priyan, V.; Jayakumar, A.; Narayanasamy, S. Magnetic Nanocellulose from *Cyperus Rotundas* Grass in the Absorptive Removal of Rare Earth Element Cerium (III): Toxicity Studies and Interpretation. *Chemosphere* **2022**, *287*, 131912, doi:10.1016/j.chemosphere.2021.131912.
110. Wang, G.; Li, F.; Li, L.; Zhao, J.; Ruan, X.; Ding, W.; Cai, J.; Lu, A.; Pei, Y. In Situ Synthesis of Ag–Fe3O4 Nanoparticles Immobilized on Pure Cellulose Microspheres as Recyclable and Biodegradable Catalysts. *ACS Omega* **2020**, *5*, 8839–8846, doi:10.1021/acsomega.0c00437.

111. Wang, G.; Yang, F.; Huang, W.; Zhou, Y.; Huang, R.; Yang, Q.; Yan, B. Recyclable Mussel-Inspired Magnetic Nanocellulose@Polydopamine–Ag Nanocatalyst for Efficient Degradation of Refractory Organic Pollutants and Bacterial Disinfection. *ACS Appl. Mater. Interfaces* **2022**, *14*, 52359–52369, doi:10.1021/acsami.2c13915.
112. da Rosa Salles, T.; da Silva Bruckamann, F.; Viana, A.R.; Krause, L.M.F.; Mortari, S.R.; Rhoden, C.R.B. Magnetic Nanocrystalline Cellulose: Azithromycin Adsorption and In Vitro Biological Activity Against Melanoma Cells. *J Polym Environ* **2022**, *30*, 2695–2713, doi:10.1007/s10924-022-02388-3.
113. Doğan, D.; Mohamed Ariff, A.H.; Leman, Z.; Metin, A.Ü. Magnetic Cellulose-Based Composite as a New Effectively Reusable Biosorbent for Cationic Dye Removal: Batch and Lab-Scale Column Studies. *Cellulose* **2023**, *30*, 7003–7029, doi:10.1007/s10570-023-05282-w.
114. Mustapić, M.; Bafti, A.; Glumac, Z.; Pavić, L.; Skoko, Ž.; Šegota, S.; Klaser, T.; Nedeljković, R.; Masud, M.K.; Alothman, A.A.; et al. Magnetic Nanocellulose: Influence of Structural Features on Conductivity and Magnetic Properties. *Cellulose* **2023**, *30*, 1149–1169, doi:10.1007/s10570-022-04956-1.
115. Kang, J.; Hu, C.; Liu, X.; Zhou, H.; Lin, X.; Gu, J. One-Pot Synthesis of Magnetic Nanocellulose/Fe3O4 Hybrids Using FeCl3 as Cellulose Hydrolytic Medium and Fe3O4 Precursor. *ACS Sustainable Chem. Eng.* **2024**, *12*, 5917–5926, doi:10.1021/acssuschemeng.3c08582.
116. Fujisawa, S.; Kaku, Y.; Kimura, S.; Saito, T. Magnetically Collectable Nanocellulose-Coated Polymer Microparticles by Emulsion Templating. *Langmuir* **2020**, *36*, 9235–9240, doi:10.1021/acs.langmuir.0c01533.
117. Tang, R.; Xu, S.; Hu, Y.; Wang, J.; Lu, C.; Wang, L.; Zhou, Z.; Liao, D.; Zhang, H.; Tong, Z. Multifunctional Nano-Cellulose Aerogel for Efficient Oil–Water Separation: Vital Roles of Magnetic Exfoliated Bentonite and Polyethyleneimine. *Separation and Purification Technology* **2023**, *314*, 123557, doi:10.1016/j.seppur.2023.123557.
118. Liu, X.; Gao, L.; Li, S.; Wang, L.; Cai, R.; Yue, T.; Yuan, Y.; Zhao, X.; Wang, Z. Cellulose-Based Magnetic Nanomaterials Immobilized Esterases as a Reusable and Effective Detoxification Agent for Patulin in Apple Juice. *Food Control* **2024**, *160*, 110381, doi:10.1016/j.foodcont.2024.110381.
119. Emam, H.E.; El-Shahat, M.; Abdelhameed, R.M. Iodine Removal Efficiently from Wastewater by Magnetic Fe3O4 Incorporated within Activated Porous Cellulose. *Industrial Crops and Products* **2023**, *193*, 116201, doi:10.1016/j.indcrop.2022.116201.
120. Amiralian, N.; Mustapic, M.; Hossain, Md.S.A.; Wang, C.; Konarova, M.; Tang, J.; Na, J.; Khan, A.; Rowan, A. Magnetic Nanocellulose: A Potential Material for Removal of Dye from Water. *Journal of Hazardous Materials* **2020**, *394*, 122571, doi:10.1016/j.jhazmat.2020.122571.
121. Amiri, Z.; Halladj, R.; Shekarriz, M.; Rashidi, A. Synthesis and Application of Recyclable Magnetic Cellulose Nanocrystals for Effective Demulsification of Water in Crude Oil Emulsions. *Environmental Pollution* **2024**, *342*, 123042, doi:10.1016/j.envpol.2023.123042.
122. Liang, Y.; Liu, F.; Wang, E.; Miao, Y.; Han, W.; Chen, Y.; Zhang, W.; Li, L.; Huang, J. Preparation of Highly Elastic Superhydrophobic CNF/Fe3O4 Based Materials Modified in Aqueous Phase for Oil-Water Separation. *International Journal of Biological Macromolecules* **2024**, *265*, 130807, doi:10.1016/j.ijbiomac.2024.130807.
123. Zhang, X.; Qiu, C.; Li, F.; Zhang, X.; Li, M.-C.; Xie, J.; de Hoop, C.F.; Qi, J.; Huang, X. Magnetic Nanocellulose-Based Adsorbent for Highly Selective Removal of Malachite Green from Mixed Dye Solution. *International Journal of Biological Macromolecules* **2023**, *253*, 126752, doi:10.1016/j.ijbiomac.2023.126752.

124. Heidarian, P.; Kouzani, A.Z. A Self-Healing Magneto-Responsive Nanocellulose Ferrogel and Flexible Soft Strain Sensor. *International Journal of Biological Macromolecules* **2023**, *234*, 123822, doi:10.1016/j.ijbiomac.2023.123822.
125. Wen, Z.; Gao, D.; Lin, J.; Li, S.; Zhang, K.; Xia, Z.; Wang, D. Magnetic Porous Cellulose Surface-Imprinted Polymers Synthetized with Assistance of Deep Eutectic Solvent for Specific Recognition and Purification of Bisphenols. *International Journal of Biological Macromolecules* **2022**, *216*, 374–387, doi:10.1016/j.ijbiomac.2022.06.187.
126. Lizundia, E.; Maceiras, A.; Vilas, J.L.; Martins, P.; Lanceros-Mendez, S. Magnetic Cellulose Nanocrystal Nanocomposites for the Development of Green Functional Materials. *Carbohydrate Polymers* **2017**, *175*, 425–432, doi:10.1016/j.carbpol.2017.08.024.
127. Ghasemi, S.; Soltanimehr, H.; Rastegari, B.; Farjadian, F. Multi-Responsive Nanocarrier Based on P(NIPAM-*Co*-DMAEA) Grafted Magnetic Cellulose for Controlled and Targeted Drug Release. *International Journal of Biological Macromolecules* **2025**, *328*, 147417, doi:10.1016/j.ijbiomac.2025.147417.
128. Suter, E.; Rutto, H.L.; Mkhize, I.G. Biodegradable Waste-Derived Cellulose/Nylon-6-Coated Iron-Oxide Nanocomposite Encapsulated with Chitosan for Enhanced Wastewater Treatment. *Cleaner Chemical Engineering* **2025**, *11*, 100190, doi:10.1016/j.clce.2025.100190.
129. Calvo-Correas, T.; Shirole, A.; Alonso-Varona, A.; Palomares, T.; Weder, C.; Corcuera, M.A.; Eceiza, A. Impact of the Combined Use of Magnetite Nanoparticles and Cellulose Nanocrystals on the Shape-Memory Behavior of Hybrid Polyurethane Bionanocomposites. *Biomacromolecules* **2020**, *21*, 2032–2042, doi:10.1021/acs.biomac.9b01764.
130. Ieamviteevanich, P.; Daneshvar, E.; Eshaq, G.; Puro, L.; Mongkolthanaruk, W.; Pinitsoontorn, S.; Bhatnagar, A. Synthesis and Characterization of a Magnetic Carbon Nanofiber Derived from Bacterial Cellulose for the Removal of Diclofenac from Water. *ACS Omega* **2022**, *7*, 7572–7584, doi:10.1021/acsomega.1c06022.
131. Gennari, A.; Mobayed, F.H.; Da Rolt Nervis, B.; Benvenutti, E.V.; Nicolodi, S.; da Silveira, N.P.; Volpato, G.; Volken de Souza, C.F. Immobilization of β-Galactosidases on Magnetic Nanocellulose: Textural, Morphological, Magnetic, and Catalytic Properties. *Biomacromolecules* **2019**, *20*, 2315–2326, doi:10.1021/acs.biomac.9b00285.
132. Chen, J.; Pang, M.; Yang, M.; Gao, F.; Zhang, B.; Zang, L.; Li, Z.; Guo, P. Chiral Effect on the Electrochemistry of Magnetic Ferrite Colloidal Nanocrystal Assembly Modified by Amino Acids. *Langmuir* **2024**, *40*, 15171–15177, doi:10.1021/acs.langmuir.4c01525.
133. Beyranvand, N.S.; Samiey, B.; Tehrani, A.D.; Soleimani, K. Graphene Oxide–Cellulose Nanowhisker Hydrogel Nanocomposite as a Novel Adsorbent for Methylene Blue. *J. Chem. Eng. Data* **2019**, *64*, 5558–5570, doi:10.1021/acs.jced.9b00655.
134. Recio-Colmenares, C.L.; Ortíz-Rios, D.; Pelayo-Vázquez, J.B.; Moreno-Medrano, E.D.; Arratia-Quijada, J.; Torres-Lubian, J.R.; Huerta-Marcial, S.T.; Mota-Morales, J.D.; Pérez-García, M.G. Polystyrene Macroporous Magnetic Nanocomposites Synthesized through Deep Eutectic Solvent-in-Oil High Internal Phase Emulsions and Fe3O4 Nanoparticles for Oil Sorption. *ACS Omega* **2022**, *7*, 21763–21774, doi:10.1021/acsomega.2c01836.
135. Sun, J.; Liu, J.; Tan, J.; Liu, H.; Jia, J.; Wang, C. Magnetic Self-Assembled Fe3O4 Colloidal Nanocrystals in Structural Color Hydrogels for Environmental Humidity Monitoring. *ACS Appl. Nano Mater.* **2024**, *7*, 21556–21564, doi:10.1021/acsanm.4c03486.
136. Zhang, J.; Fang, Q.; Duan, J.; Xu, H.; Xu, H.; Xuan, S. Magnetically Separable Nanocatalyst with the Fe3O4 Core and Polydopamine-Sandwiched Au Nanocrystal Shell. *Langmuir* **2018**, *34*, 4298–4306, doi:10.1021/acs.langmuir.8b00302.

137. Chen, T.; Zhao, Q.; Meng, X.; Li, Y.; Peng, H.; Whittaker, A.K.; Zhu, S. Ultrasensitive Magnetic Tuning of Optical Properties of Films of Cholesteric Cellulose Nanocrystals. *ACS Nano* **2020**, *14*, 9440–9448, doi:10.1021/acsnano.0c00506.
138. Khalilzadeh, M.A.; Tajik, S.; Beitollahi, H.; Venditti, R.A. Green Synthesis of Magnetic Nanocomposite with Iron Oxide Deposited on Cellulose Nanocrystals with Copper (Fe3O4@CNC/Cu): Investigation of Catalytic Activity for the Development of a Venlafaxine Electrochemical Sensor. *Ind. Eng. Chem. Res.* **2020**, *59*, 4219–4228, doi:10.1021/acs.iecr.9b06214.
